# Global estimation of bankfull river discharge reveals distinct flood recurrences across climate zones

**DOI:** 10.1038/s41467-026-76433-3

**Published:** 2026-08-18

**Authors:** Yinxue Liu, Michel Wortmann, Laurence Hawker, Jeffrey Neal, Jiabo Yin, Marcus Suassuna Santos, Bailey Anderson, Richard Boothroyd, Andrew Nicholas, Greg Sambrook Smith, Philip J. Ashworth, Hannah Cloke, Solomon Gebrechorkos, Julian Leyland, Boen Zhang, Ellie Vahidi, Helen Griffith, Pauline Delorme, Stuart James McLelland, Daniel R. Parsons, Stephen E. Darby, Louise Slater

**Affiliations:** 1 School of Geography and the Environment, University of Oxford, South Parks Road, Oxford, UK; 2 Geography and Environment, Loughborough University, Loughborough, UK; 3 European Centre for Medium-Range Weather Forecasts (ECMWF), Shinfield Park, Reading, UK; 4 School of Geographical Sciences, University of Bristol, University Road, Bristol, UK; 5 State Key Laboratory of Water Resources Engineering and Management, Wuhan University, Wuhan, China; 6 Department of Hydrology, Geological Survey of Brazil – SGB/CPRM, Brasilia, Brazil; 7 WSL Institute for Snow and Avalanche Research SLF, Davos Dorf, Switzerland; 8 Institute for Atmospheric and Climate Science, ETH Zurich, Zurich, Switzerland; 9 Department of Geography and Planning, School of Environmental Sciences, University of Liverpool, Liverpool, UK; 10 Geography, Faculty of Environment, Science and Economy, University of Exeter, Exeter, UK; 11 School of Geography, Earth and Environmental Sciences, University of Birmingham, Birmingham, UK; 12 Department of Earth Science and Environmental Change, University of Illinois, Urbana-Champaign, IL USA; 13 Department of Meteorology, University of Reading, Reading, UK; 14 Department of Geography & Environmental Science, University of Reading, Reading, UK; 15 School of Geography and Environmental Sciences, University of Southampton, Southampton, UK; 16 JBA Consulting, 1 Broughton Park, Old Lane North, Broughton, Skipton, UK; 17 Energy and Environment Institute, University of Hull, Hull, UK; 18 Vice Chancellor’s Office, Loughborough University, Loughborough, UK

**Keywords:** Natural hazards, Hydrology

## Abstract

Bankfull discharge, the maximum flow a river can convey before spilling over its banks, is central to modelling flood risk and understanding river-channel evolution. Global flood inundation models assume a 2-year return period for bankfull conditions, but this assumption remains untested globally. Here we use observations and machine learning to estimate bankfull discharge at ~1-km resolution along a recently developed global river network. We show that the widely used 2-year discharge reasonably approximates bankfull-flow magnitude, but bankfull return periods vary substantially within and across climate zones. Bankfull conditions occur more frequently in tropical and temperate regions (median return periods of 1.5 and 1.8 years; interquartile ranges of 2.5 and 3.2 years, respectively) and less frequently in cold and arid regions (2.8 and 4.3 years; interquartile ranges of 4.8 and 6.0 years). This variation across climate zones provides a basis for improving the representation of channel capacity in global flood models.

## Abstract (web summary)

This study shows that bankfull discharge, the flow a river carries before overflowing its banks, recurs at different rates across climate zones, suggesting that the widely used 2-year assumption may bias flood estimates.

## Introduction

Flooding is the most frequent and costly weather-related hazard, with several million people affected every year^1^. With the changing climate and growing population, river flood risk is projected to increase substantially in many locations, threatening lives, livelihoods, agriculture and infrastructure^2–4^. Global Flood Models (GFMs) are widely used tools for estimating flood hazard and risk and for understanding their future changes under climate change at the global scale^5,6^. To simulate inundation, GFMs must first estimate channel conveyance capacity to initiate flow routing^7^. This river conveyance capacity is often quantified by the maximum flow rate, or bankfull river discharge (Q_BF_), that a river can carry before it spills over its banks. This value is fundamental not only for estimating river bankfull geometry^8,9^ and simulating flood inundation^10,11^, but also for modelling sediment transport^12,13^, investigating river geomorphological evolution^14,15^, estimating river carbon fluxes^16,17^, and understanding riverine habitats and migration^18^.

Due to the lack of global observations, GFMs typically employ a spatially and temporally uniform recurrence interval of 2-year flow to approximate the bankfull discharge^5,11,19^; other models derive channel capacity from empirical hydraulic-geometry relations^20^ or a calibrated recurrence parameter^21^, but apply these relations uniformly across the model domain. The 2-year assumption was originally based on field survey data from a sample of 36 rivers from the USA and one river in India, carried out in the 1950s^22^. In 31 of the 37 rivers surveyed, the bankfull discharge corresponded to the flow with a recurrence interval of 1 to 3 years. Although many subsequent studies have found substantial variability in the recurrence interval of bankfull discharge^23–27^, this variability has not yet been quantified at the global scale and is therefore not represented in GFMs, potentially introducing systematic biases in some regions in flood risk estimation. For example, if a river has a conveyance capacity smaller than the 2-year assumption implies, channels convey less water before overtopping than models assume, so real flood extents exceed modelled ones and flood risk is underestimated. Flood models are highly sensitive to this threshold: even small differences in bankfull discharge can substantially influence modelled inundation as they propagate through flood-wave dynamics, particularly for smaller, high-frequency events that are critical for quantitative risk assessment^20,28,29^.

The two-year return period flow assumption has been widely adopted due to the lack of reliable, spatially consistent observations of bankfull discharge. A few studies have examined bankfull discharge over broader spatial extents, primarily in the USA and Europe^27,30^, but these have focused predominantly on individual river basins or remain confined within administrative boundaries. Since direct measurement of bankfull discharge is impractical due to its infrequent occurrence, it is typically estimated by interpolating the discharge corresponding to the bankfull water level based on the stage-discharge relationship or cross-section measurements from field surveys. Collecting such data is time-intensive and costly, and therefore, bankfull discharge observations are available for only a very limited number of rivers worldwide. Consequently, relying on field measurements of bankfull discharge for all river reaches globally is infeasible, and the wide range of river characteristics worldwide makes accurate estimation of bankfull discharge challenging.

Accurately estimating bankfull discharge has become more pressing with the development of GFMs^5,19^. Several recently developed global datasets have provided new opportunities to estimate bankfull discharge. These datasets include the location and connectivity of rivers^31,32^, observation-based river width datasets^33^, and dam and reservoir information^34^. Additionally, machine learning is increasingly being used to produce new geophysical datasets^35,36^.

In this work, we use a diverse suite of recently developed global datasets, coupled with machine-learning approaches, to estimate bankfull discharge for ~2.87 million km of rivers wider than 30 m worldwide. We provide spatially distributed global estimates of bankfull river discharge (GQBF), showing distinct climatic patterns of bankfull discharge return periods, and quantify the bias of the common 2-year return period bankfull discharge assumption.

## Results

### Global estimates of bankfull discharge

To estimate bankfull discharge for the world’s ungauged rivers, we first developed an observed bankfull discharge (Q_BFobs_) dataset from nine sources, including literature values, river cross-section measurements, and stage-discharge relationships at gauged river transects. The resulting dataset consists of 2,657 observations with a wide range of locations and bankfull discharge magnitude (details in Methods, Supplementary Text S1, and Figs. S1–4). We based our bankfull river discharge estimation on a recently developed hydrography, Global River Topology (GRIT)^31^, using GRIT’s ~1 km river reaches as the spatial scale to represent the variation in bankfull discharge. We included all GRIT river reaches that coincided with the Global River Width from Landsat (GRWL)^33^ river masks (with overlapping ratio ≥0.5). This selection retains river reaches with satellite-derived width measurements no narrower than 30 m, resulting in a total length of ~2.87 million km. The spatial distribution and density of Q_BF_ observations aggregated within GRIT domains (60 global river basin groups) are provided in Supplementary Fig. S5. For those river reaches, we then prepared 31 predictors describing river characteristics and river discharge-related climate indicators (Methods and Supplementary Table S1). Based on the Q_BFobs_ dataset and the predictors, we developed a Random Forest (RF) model and then used it to estimate the bankfull discharge (Q_BFest_) for each river reach across the full ~2.87 million km of the GRIT hydrography. The density distribution of the predictors used for model training and global estimation can be found in Supplementary Fig. S6.

Our training of the model on 80% of the observed dataset and testing on the remaining 20% (excluded from the training dataset) showed consistently high performance, with both R^2^ values exceeding 0.79 (Supplementary Figs. S7, 8). The RF model exhibits some over-estimation, with Median Percentage Errors (MdPEs) of 11% and 21% (training, testing), which is similar to regional flood frequency analysis estimation errors for 50-year flood magnitudes obtained in tropical and cold climates^37^. The Mean Percentage Errors (MPEs) are 28 and 44% (training, testing), which are greater than those reported in a previous study conducted in Europe at a coarser grid resolution of 5 arc min^27^ (22.8%). This difference likely reflects the greater local variability present at finer spatial resolutions, which is more difficult for the model to represent accurately. In addition, MPE is particularly sensitive to extreme values, which are more prevalent at finer resolutions. Aggregation to coarser resolutions may smooth these extremes and reduce variability, thereby reducing overall errors. The normalised Root Mean Square Error (nRMSE) of the training and testing datasets is 1.32 and 1.66, respectively (equations of the error metrics R^2^, MdPE, MPE, and nRMSE can be found in Supplementary Text S2).

Among the four Köppen climate regions (tropical, temperate, cold, and arid)^38^, the Q_BFest_ has the smallest error (nRMSE = 0.7, testing) in the cold region, likely due to the proportionally larger training sample within this climate region (Supplementary Fig. S8). The tropical region experiences the most pronounced overestimation, but also underestimations in reaches with bankfull discharge values greater than ~7000 m^3^s^-1^ (Supplementary Fig. S9).

We further tested the model’s spatial generalisation performance. Our leave-one-basin-out validation indicates that the RF model generally captures the spatial variability of bankfull discharge well across most basin groups, although performance is lower in a few cases (Supplementary Fig. S10). Performance is relatively poorer in basin groups dominated by arid and tropical climates, where observations of bankfull discharge are more limited compared to temperate and cold regions. Estimates are less accurate for small and very large rivers, those with bankfull discharge below 20 m³s⁻¹ or above 7000 m³s⁻¹, possibly reflecting uncertainties in river-width measurements (with higher width uncertainty for rivers narrower than 90 m^33^) and the scarcity of bankfull discharge observations for very large rivers. Error metrics improve when these size classes are excluded (Supplementary Fig. S9), and corresponding metrics for different ranges of bankfull discharge are provided in Supplementary Table S2.

**Fig. 1:**
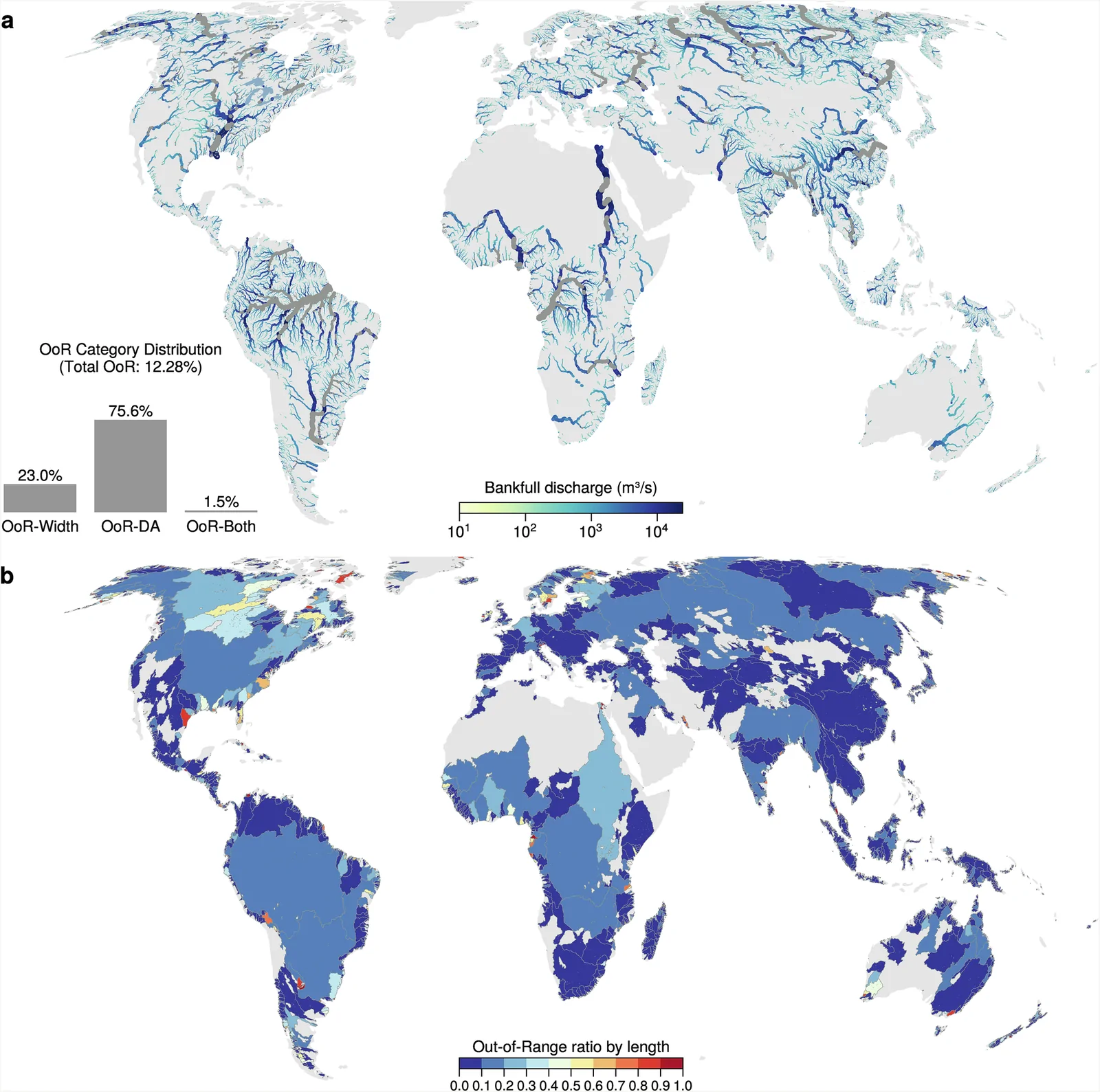
Bankfull river discharge across ~2.87 million km of global rivers, estimated at ~1 km resolution. **a** Bankfull river discharge along the GRIT global river network. Estimations are displayed at GRIT segment scale (aggregated median value of reaches) for visual clarity. Out-of-range estimations are coloured in grey. The left inset shows the proportion of out-of-range river length attributed to river width, drainage area and both. Percentages are rounded and may not sum to exactly 100. **b** Fraction of river-network length that is out-of-range within each GRIT basin, defined as the proportion of total river-network length where river width or drainage area falls outside the training-data range.

Using the validated model, we estimated the river reach-scale bankfull discharge across ~2.87 million km of the GRIT hydrography. For clarity, the estimates are aggregated to the segment-scale in Fig. 1a. The GRIT hydrography defines a river segment as the channel section between two confluences, or between a headwater and its first confluence, and subdivides each segment into reaches of no more than 1 km in length. In the case of multi-threaded rivers, each thread has at least one estimated bankfull discharge value, depending on the length of the thread (Supplementary Fig. S11).

To limit uncertainty in bankfull discharge estimations arising from extrapolation, we flagged locations with predictor values beyond the training range, where model estimations are inherently unreliable^39^. We used two predictors, the DEM-derived drainage area and observation-based river width, to identify out-of-range locations, i.e., reaches with values outside 30–1673 m for river width or outside 50-3,425,560 km^2^ for drainage area. Such reaches account for 12.28% of the total ~2.87 million km of river length. Most are flagged because drainage area exceeds the upper limit of the training range (75.6% of out-of-range reaches), fewer because river width falls outside its range (23.0%), and few on both criteria (1.5%; Fig. 1a). Across global river basins, the out-of-range length ratio is generally below 0.2 (Fig. 1b). These reaches are concentrated along high-order mainstems, particularly in the Amazon, Congo, Yangtze, Mississippi, Nile river basins, and along several major Arctic-draining rivers in northern North America and Eurasia (Fig. 1a). The distribution of junction counts for all mapped and out-of-range rivers is compared in Supplementary Fig. S12.

### Return period of bankfull river discharge varies across climate regions

**Fig. 2:**
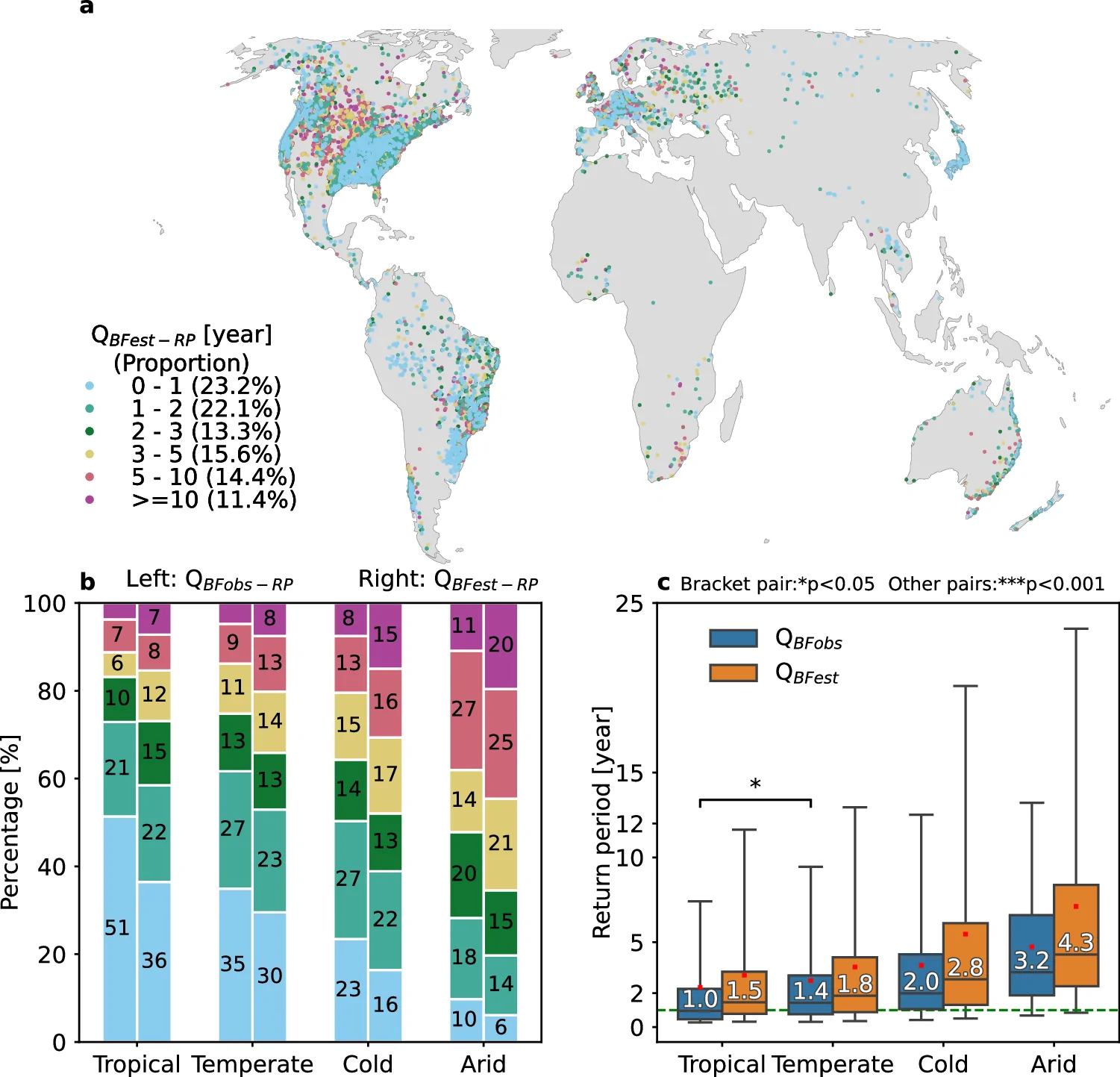
Variation of bankfull river discharge return periods (Q_BF-RP_) across four climate regions: tropical, temperate, cold, and arid. **a** Estimated bankfull discharge return periods (Q_BFest-RP_), with colours indicating return period ranges (0–1, 1–2, 2–3, 3–5, 5–10, and ≥10 years); the proportion of sites in each range is given in brackets in the inset legend. **b** Proportion of gauging stations in each return period category (coloured, colour scale is the same as (**a**) for each climate region, with observed values shown on the left bar and estimated values on the right bar. **c** Distribution of observed and estimated return periods (Q_BFobs-RP_, Q_BFest-RP_) in each climate region. The boxplot limits (horizontal lines), from bottom to top, mark the 5, 25, 50, 75, and 95^th^ percentiles, respectively; red squares mark the mean values; labels represent the median. All pairwise differences between climate regions are significant at *p* < 0.001, for both Q_BFobs-RP_ and Q_BFest-RP_, except the Tropical-Temperate comparison for Q_BFobs-RP_ (bracket; *p* < 0.05).

Based on daily discharge records at 8519 gauging stations, we applied a peak-over-threshold approach to extract flood events. We then fitted a simple flood magnitude-recurrence relationship (see Methods, Flood frequency analysis) to derive return periods for both Q_BFobs_ and Q_BFest_. We find the return periods corresponding to bankfull river discharge (Q_BF-RP_) vary widely across the globe. Overall, almost a quarter of sites (23.2%) are expected to reach Q_BF_ more than once per year on average (Q_BF-RP_ < 1 year). This proportion exceeds those with Q_BF_ return periods of 1–2 years (22.1%) and 2–3 years (13.3%). We find 15.6% of sites with bankfull discharge corresponding to a return period of 3–5 years, 14.4% of 5–10 years, and 11.4% of periods ≥10 years. The spatial distribution indicates that longer return periods ( > 3 years) associated with Q_BF_ occur predominantly in arid regions (e.g., the southwestern United States) but are less common in tropical climates (e.g., the Amazon basin) (Fig. 2a).

We show the proportion of sites across six bankfull discharge return period ranges within each climate region (Fig. 2b). Notably, the observed (estimated) bankfull return period is smaller than 1 year in 51% (36%) of tropical sites, whereas in the arid region, 52% (66%) of sites have return periods exceeding 3 years.

The climate-region median of observed (estimated) Q_BF-RP_ increases from 1.0 years (1.5) in tropical regions, to 1.4 years (1.8) in temperate regions, 2.0 years (2.8) in cold regions, and 3.2 years (4.3) in arid regions. The variation in the Q_BF-RP_ is also pronounced within each climate region. The observed (estimated) Q_BF-RP_ interquartile range is [0.5, 2.2] (0.8, 3.3) for tropical regions, [0.8, 3.0] (0.9, 4.1) for temperate regions, [1.1, 4.3] (1.3, 6.1) for cold regions, and [1.9, 6.6] (2.4, 8.4) for arid climate regions (Fig. 2c). For both observed and estimated bankfull discharge, the distribution of Q_BF-RPs_ differs significantly across climate regions (Kruskal–Wallis H test, *p* < 0.001). Post hoc Dunn’s tests indicate significant differences among nearly all pairs of climate regions (*p* < 0.001), except between tropical and temperate climates for Q_BFobs-RP_, for which the difference was weaker (*p* < 0.05). These results are robust to the choice of the flood frequency analysis method (Supplementary Fig S.13-14).

Q_BFobs_ and Q_BFest_ show a similar distribution of return periods within each climate (Fig. 2b, c). This consistency, to some extent, supports the reliability of our bankfull discharge estimations beyond the testing sites. The slightly higher medians and wider ranges in Q_BFest-RP_ may reflect differences in the spatial and climatic distribution of the estimated and observed sites, as well as differences introduced by the estimation procedure.

**Fig. 3:**
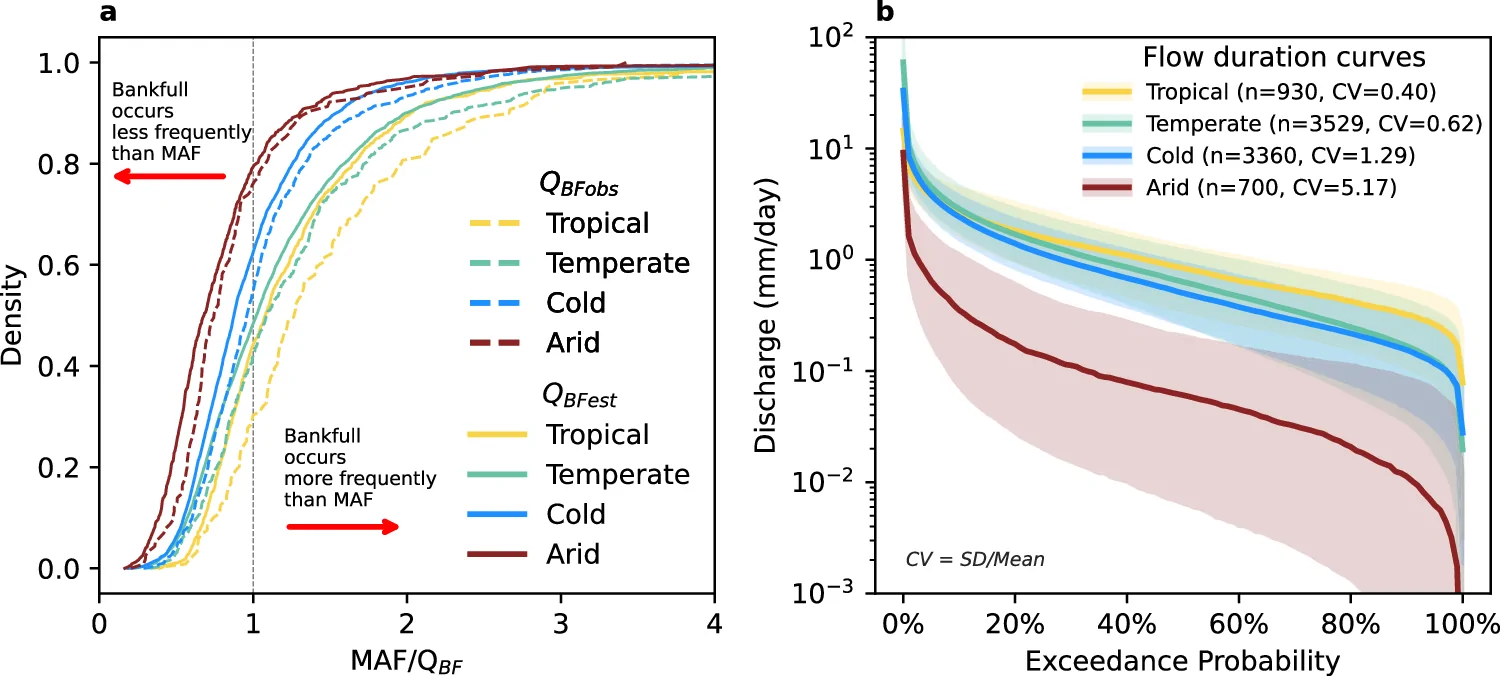
Variability in bankfull discharge and daily streamflow across four climate regions: tropical, temperate, cold, and arid. **a** Probability density of the ratio between Mean Annual Flood (MAF) and bankfull discharge, shown for both observed (Q_BFobs_) and estimated (Q_BFest_) values across climate regions. **b** Cumulative flow duration curves showing daily discharge versus exceedance probability in four climate regions, based on 8519 gauges. Solid lines indicate the median; transparent ribbons show the 25–75th percentiles across all stations within each climate. The number of stations and coefficient of variation (CV) of streamflow are shown in the legend.

The ratio of bankfull discharge (QBF) to mean annual flood (MAF) reveals how bankfull frequency varies with climate across the study sites. The proportion of sites with small MAF/Q_BF_ ratios, where bankfull discharge is greater relative to the mean annual flood and therefore occurs infrequently, was highest in the arid region (80% of sites with a ratio ≤ 1; Fig. 3a). In contrast, in the tropical regions, MAF/Q_BF_ ratios greater than 1 are more prevalent, meaning bankfull occurs more than once per year, on average. This pattern is consistent across both the observed and estimated values (Fig. 3a). Accordingly, tropical rivers show the most sustained flow regimes (low coefficient of variation) and flatter duration curve distributions, while arid rivers exhibit more variable flow regimes (Fig. 3b).

### Biases in the commonly used bankfull river discharge assumption

**Fig. 4:**
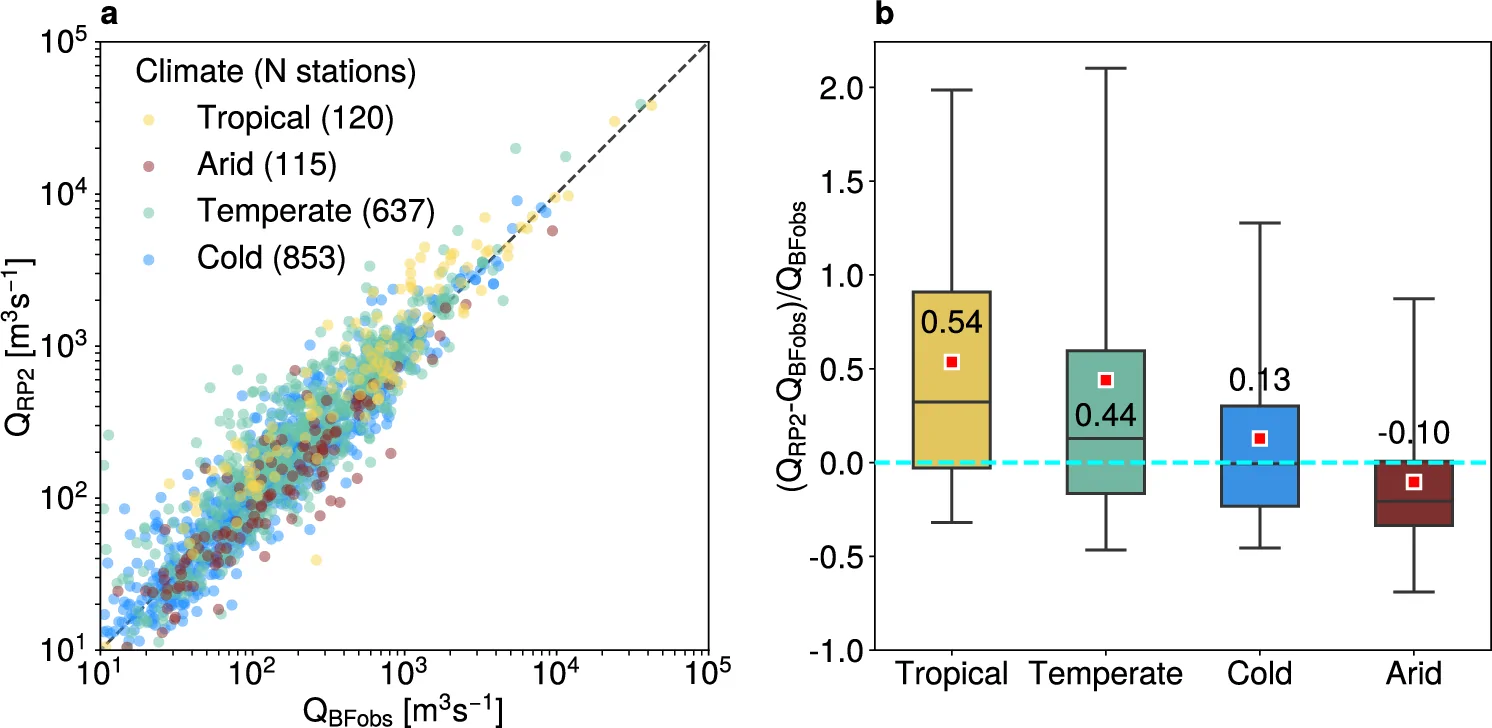
Biases associated with the widely used 2-year return-period bankfull river discharge (Q_RP2_) assumption across four climate regions: tropical, temperate, cold, and arid. **a** Scatter plot of 2-year return period discharge (Q_RP2_; y-axis) and observed bankfull discharge (Q_BFobs_; x-axis) in log scale. **b** Distribution of the 2-year return period bias compared to bankfull discharge observations in each climate region. Horizontal boxplot lines from bottom to top mark the 5, 25, 50, 75, and 95th percentiles, respectively; red squares mark the mean values with numbers displayed. Horizontal cyan line marks zero bias.

By comparing observed bankfull discharge with the 2-year return period discharge (Q_RP2_), computed from long-term daily streamflow observations using the same method as for the Q_BF-RP_ (Methods, Flood frequency analysis), we assess bias in the widely used 2-year river channel capacity assumption and its variation across climate regions. Overall, the 2-year return period discharge aligns broadly with observed bankfull discharge (Fig. 4a). The bias is nevertheless substantial, and its direction varies systematically by climate region. The use of a 2-year return period discharge overestimates bankfull discharge in tropical regions by 54 ± 78% (mean ± standard deviation) and in temperate regions by 44 ± 141%. The overestimation is smallest in cold regions (13 ± 59%). In arid regions, however, the direction of bias is reversed, and the 2-year return period discharge underestimates bankfull discharge by 10 ± 51% (Fig. 4b).

## Discussion

Although the 2-year discharge broadly matches the magnitude of bankfull flow, the assumption of a spatially uniform two-year recurrence does not hold across climate regions. Instead, we demonstrate systematic differences across climate regions, with median recurrence intervals ranging from ~1.5 years in the tropics to ~4.3 years in arid regions. Our analysis suggests that the frequency of bankfull discharge is influenced by the dominant flood-generating mechanisms in each climate region. In arid regions, floods are produced by episodic, high-intensity rainfall, with a large proportion of annual streamflow often derived from a small number of discrete flood events^40^. In humid, perennial tropical catchments, intense wet-season rainfall frequently produces flows near or above bankfull. In cold regions, large floods commonly result from ice breakup or rapid snowmelt, which can exceed bankfull discharge. Such events are often hydrologically complex and show greater interannual variability than rainfall-generated floods in tropical and temperate settings^41,42^, which may help explain the less frequent bankfull discharges observed in cold climates. These interpretations are consistent with established hydrological theory and with the climatic differences in flow regimes shown by flow duration curves (Fig. 3b). In tropical regions, the relatively flat curves indicate more sustained flow regimes, consistent with bankfull conditions reached relatively frequently. In arid regions, the steeper curves indicate more variable flow regimes, consistent with less frequent attainment of bankfull discharge. Climatic variation in bankfull discharge return periods, therefore, provides a basis for linking hydrological forcing to river geomorphology and hydraulic theory. Variability within climate regions may also reflect basin-scale characteristics such as drainage area, slope, and sediment size.

Our findings have direct implications for global river flood modelling. We highlight the importance of incorporating climatic variation when estimating channel capacity at regional to continental scales. Global Flood Models that adopt the uniform 2-year assumption may bias simulated flood depth and extent, underestimating them in the tropics, where actual recurrence intervals are shorter, and overestimating them in arid regions, where recurrence intervals are longer. Using climate-specific median recurrence intervals provides an improved yet simple channel-capacity assumption compared with the spatially uniform 2-year assumption. Adopting these values may affect current global flood hazard and risk assessments, given the sensitivity of inundation models to bankfull discharge^20,28^. The median values and associated uncertainties reported here may be refined as additional observational estimates of bankfull discharge become available. Nevertheless, such refinements are not expected to alter the large-scale climate-associated spatial pattern identified in this study. Indeed, we obtain a consistent climatic ordering when the analysis is repeated using an independently sourced, systematically measured dataset, supporting the increase from tropical to temperate, cold, and arid climates (Supplementary Fig. S15).

Nevertheless, climate-specific median return periods are unlikely to be sufficiently accurate for high-resolution modelling, since substantial variation in bankfull discharge return periods exists within each climate region. The intra-climate heterogeneity of bankfull discharge return periods found in this work aligns with previous observations in localised areas that showed highly variable bankfull recurrence intervals^43,44^. Consequently, the true channel capacity of individual river reaches can deviate considerably from the climate-specific medians. Our GQBF estimation, which integrates a range of factors influencing bankfull discharge and is provided at high resolution, has the potential to improve the realism of global flood inundation simulations and, in turn, inform global risk assessments, infrastructure design, and climate adaptation strategies.

Uncertainties remain at out-of-range locations, where predictor values fall outside the training domain. Although out-of-range locations were identified using drainage area and river width, some reaches may still have combinations of predictor values that are poorly represented in the training data. In such cases, bankfull discharge may be approximated using return-period estimates from nearby in-range reaches, where these are hydrologically comparable. Where suitable neighbouring estimates are unavailable, bankfull discharge may be approximated using the discharge associated with the median return period for each climate zone, derived from flood frequency analysis or estimated from bankfull hydraulic geometry relationships based on bankfull width^7,35^, depth^45^ and slope. These alternatives are themselves uncertain, reflecting intra-climate variability and poorly constrained geometric assumptions, particularly for channel depth. In addition, predictions inevitably contain noise at the scale of individual river reaches, and caution is therefore warranted when applying this dataset at local scales. Overall, achieving globally accurate reach-level estimates of bankfull discharge remains challenging, highlighting the need for continued data collection and improved modelling approaches.

In addition to these model-related limitations, global estimates of bankfull discharge are affected by uncertainties stemming from limited, spatially biased observational data. These include methodological differences among the source datasets, uncertainty in high-flow measurements, temporal and spatial variability of bankfull discharge within river reaches, a biased representation towards perennial rivers, and limited information on channel types and river regulation. (1) Field measurements of the discharge near bankfull can have uncertainties of 10-20% or more^46^, making accurate estimation difficult because the target variable is itself uncertain. (2) Temporal non-stationarity in bankfull conveyance capacity is also well documented^47,48^. Owing to the sparsity of bankfull discharge observations available for global model training, we do not account for temporal changes in bankfull discharge. In locations with multiple estimates of bankfull discharge, the mean of all measurements was used. (3) Bankfull discharge is typically measured at a specific cross-section, often corresponding to a gauging station, rather than at reach-scale. We assume our point-based measurements are representative of the average bankfull discharge for the corresponding GRIT river reach ( ≤ 1 km in length), and therefore, ignore any potential spatial variation within the reach. (4) Gauged sites may not be representative of global diversity in conveyance patterns. Gauged rivers tend to be relatively large, perennial rivers draining human-occupied watersheds^49^; this inevitably introduces a bias in bankfull discharge estimation relative to more natural or ephemeral river systems. (5) Bankfull discharge may vary across channel types, such as alluvial and bedrock rivers^50^, but limited high-resolution information on channel type prevents us from accounting for this effect. (6) Human impacts on conveyance may not be fully represented. Although some model predictors, such as dams, reservoirs, and impervious surface ratios, partially reflect human interventions, they do not fully capture the hydraulic complexity of flow regulation. Rivers with engineered flood defences often exhibit bankfull discharge magnitudes that exceed those of natural systems, as the effective bankfull in defended rivers is typically set by the design flood protection standard rather than by natural channel-forming processes. Consequently, users should exercise caution when applying this dataset to regulated or engineered river reaches.

Further refinement of the bankfull discharge model could help overcome these limitations. First, increasing the number and spatial coverage of observations is likely to improve the model, although such data are challenging to obtain. Remotely-sensed information, such as the river water surface elevation retrieved from the Surface Water and Ocean Topography (SWOT) satellite^51,52^ may be helpful for bankfull discharge estimation, particularly for large rivers. Second, upgrading existing predictors with higher-resolution data once it becomes available could enhance model performance. Additional predictors could provide useful information, such as terrestrial water storage, glacier extent, topographic information derived from digital elevation models, sediment size and transport, or more detailed information on channel modification and river regulation. Finally, there is scope to extend the work to smaller rivers and develop regional models to provide higher-resolution estimates of channel conveyance and other river attributes such as channel depth.

This work quantifies the recurrence interval at which rivers reach bankfull and finds that it varies systematically across global climate regions, departing from the two-year interval assumed in global flood inundation models. We estimate bankfull discharge at ~1 km spatial resolution for ~2.87 million km of rivers globally, using 2657 bankfull discharge observations compiled in this study, the recently published bifurcating Global River Topology hydrography (GRIT^31^), and globally distributed predictors reflecting catchment and river characteristics, climate, landscape, and human intervention. By quantifying variation in the return period of bankfull discharge across and within climate regions globally, we highlight systematic biases associated with using a spatially invariant bankfull return period. We show that the return period of bankfull discharge (estimated) increases systematically across climate regions, from a median of 1.5 years in tropical regions (25^th^-75th percentile: 0.8-3.3) to 1.8 (0.9-4.1) in temperate, 2.8 (1.3-6.1) in cold, and 4.3 (2.4-8.4) in arid regions. Observed return periods follow the same ordering at systematically shorter values (medians of 1.0, 1.4, 2.0 and 3.2 years), consistent with the slight positive bias of the model. Bankfull recurrence is closest to two years in cold regions (observed median 2.0 years), whereas a spatially invariant 2-year return period discharge overestimates bankfull discharge in tropical and temperate regions (54% ± 78% and 44% ± 141%, mean ± s.d.) and underestimates it in arid regions (10% ± 51%). Hence, the assumption that bankfull discharge occurs consistently every two years on average is unlikely to be appropriate everywhere, especially in tropical and arid regions. In river basins where long-term streamflow data are available, bankfull discharge may be approximated using the discharge corresponding to the median Q_BF_ return period for the relevant climate zone. Our bankfull discharge estimation helps fill data gaps in ungauged rivers across the globe, allowing users to select bankfull return period estimates based either on the relevant climate region or at the GRIT reach-scale.

This work provides a means to refine the commonly used assumption of a homogeneous conveyance-capacity return period in GFMs by representing the spatial variation in the bankfull discharge return period, thereby offering potential improvements to current modelling of global fluvial flood hazard and risk. These findings provide a globally consistent benchmark for advancing riverine and water resources research across hydrology, geomorphology, and ecology.

## Methods

### The global river topology (GRIT) hydrography

We used the recently developed Global River Topology (GRIT) hydrography^31,32^ as the basis for developing the global bankfull discharge model. GRIT is a high-resolution bifurcating global hydrography, developed from a satellite-derived river mask^33^ and an improved high-resolution global Digital Elevation Model (DEM): the Forest and Buildings removed Copernicus DEM (FABDEM^53^). The centrelines were generated from the GRWL dataset, and the OpenStreetMap water layer was used to maximise the topographic accuracy of the river network outside of the GRWL mask. GRIT represents bifurcating and multi-threaded rivers, which are not resolved in previous global hydrographies HydroSHEDS^54^ or MERIT Hydro^55^. It represents all rivers with a drainage area larger than 50 km^2^ or rivers wider than 30 m, with an average reach length of 1 km. We estimated the bankfull discharge for all the river reaches wider than 30 m in the GRIT network except in the Polar Köppen climate regions^38^, where river channels are likely to be frozen for much of the year.

### Bankfull discharge reference data

We assume that the point-based bankfull discharge values, typically measured at gauging stations, are representative of discharge across the whole river reach. The observed bankfull discharge locations were matched to river reaches on the GRIT network using a snapping approach, pairing the Q_BFobs_ with the nearest river reach, defined by the distance from the Q_BFobs_ to the river centreline. The Q_BFobs_ values were discarded if this distance exceeded 1 km, or the drainage area of the Q_BFobs_ did not match the drainage area of the paired river reach (deviated by more than 20%). We obtained the climate region of the observed bankfull discharge sites using the Köppen climate zone dataset^38^. Considering the high seasonal variation of the river water status (free-flowing or frozen), sites in the Polar region were excluded. After applying the filtering steps, this left a total of 2657 Q_BFobs_ values to train and test the RF model. These Q_BFobs_ values were distributed across four climate regions (minimum number of 156 sites), with bankfull discharge values ranging from 10 to 42,235 m^3^s^−1^ (median value of 159 m^3^s^-1^). Temporal changes in bankfull river discharge associated with river channel evolution were not considered in this paper due to the very small number of sites where such observations exist. More details of this dataset can be found in the Supplementary Text S1 and Figs. S1–4.

### Bankfull discharge predictors

A selection of hydrological, landscape and climatic indicators known to control river conveyance capacity (bankfull discharge) was identified based on the literature^48,56,57^ and used as predictors for estimating the bankfull discharge. These indicators include drainage area, annual mean discharge, annual total runoff, river widths at multiple inundation frequencies, river sinuosity, reservoir capacity, elevation, slope, water table depth, soil moisture, soil thickness, Leaf Area Index (LAI), Fraction of Absorbed Photosynthetically Active Radiation (FAPAR), impervious percentage area, and a suite of climate indicators (temperature, precipitation, aridity index, snow cover fraction), detailed below.

Depending on the indicator type, different aggregation approaches were used to process these indicators as GRIT river reach predictors. These approaches include Catchment Sum (CS: total value accumulated from the upstream catchment), Catchment Average (CA: mean of all raster values within the catchment), Catchment Majority (CM: the most common value among all raster values within the catchment), Reach Median (RM: median value of all raster values within the river reach), Reach Average (RA: mean value of all raster values within the river reach). It is worth noting that at bifurcated rivers, the calculation of CS follows the partitioning approach in the GRIT network; no extra processing is done for the RM and the RA as each thread of a bifurcated river is treated as an individual river reach. Where predictors were available over multiple years, time-averaged values were computed. The predictors and processing details are described below (Supplementary Table S1).

Drainage area has a controlling effect on river channel size and discharge^9,25,58^. The upstream drainage area was estimated for each reach from the GRIT network attributes. This drainage area is inherited from the GRIT river network, which takes a width-based partitioning approach at river bifurcations^31,59^. The discharge and runoff predictors are produced from the Global Flood Awareness System (GLOFAS) global annual mean discharge and annual total runoff version 4.0, which are re-analysis values provided by the European Centre for Medium-Range Weather Forecasts (ECMWF) at a spatial resolution of 0.05° ( ~ 5 km at the equator) for the period 1993 to 2022^60^. Both variables were converted to a volumetric flow rate (m³s⁻¹) and accumulated along the GRIT drainage network, so that each reach’s predictor value represents the total upstream contribution routed to that reach.

The GRWL dataset is an observation-based, global compilation of river planform geometry at mean annual discharge^33^, derived from thousands of Landsat images acquired in the months during which the mean annual discharge occurs at each location. Here, the river width attribute was calculated from the GRWL river mask by taking the RM of the perpendicular distance from the GRIT river centreline to the river bank^31^. We only considered rivers which overlap with the GRWL river mask (grwl_overlap ≥ 0.5). To account for the uncertainty of the GRIT river width in our model, we included the overlap ratio^31^ between GRIT river and the GRWL river mask as an additional predictor.

We generated the Wetted River Widths (WRWs) dataset from the Landsat surface water occurrence data^61^. We use the term “wetted river widths” to describe the dynamic nature of the river width from various water occurrence values. The Landsat surface water occurrence data is a global surface water mask which indicates the ratio of the average monthly sum of water detections to the monthly sum of all valid observations over a 38-year period (1984–2021). We calculated the WRWs as the wetted area connected to the reach divided by the overlap length between the river and the water layer. The calculation is applied to each river reach directly, resulting in the WRWs predictors. We extract these values for different water occurrence values (50, 40, 30, 20, 10, 1%), where a smaller occurrence value typically implies larger discharge events. We include several frequencies of occurrence because they provide different information on the surface extent (and morphology) of the channel based on the frequency with which the water occurs in each location (Supplementary Fig. S16).

River sinuosity controls channel gradient and hence impacts discharge conveyance^8^. Sinuosity was calculated as the river planform distance divided by the straight-line distance^62^ for each segment in the GRIT network, using the GRASS GIS line sinuosity tool (https://grass.osgeo.org/grass83/manuals/v.to.db.html).

The Global Reservoir and Dam (GRanD) database is a global compilation of existing (until 2016) dam and reservoir data. GRanD includes dams and reservoirs with capacity greater than 0.1 km^3^, with a cumulative storage capacity of 6864 km^3^^34^. The reservoir capacity predictor was computed by mapping the reservoir location (vector) to the nearest river reach and employing the CS reservoir capacity as the reservoir predictor.

Elevation and slope were calculated for each river reach using the CA method from the recent Forest And Buildings removed DEM (FABDEM, ~30 m spatial resolution)^53^. FABDEM is an artefact error-removed DEM based on the recently released 30 m global DEM, Copernicus DEM. The Copernicus DEM was resampled from the high-resolution ( ~ 12 m) Interferometry Synthetic Aperture Radar (InSAR) acquired global DEM, which has been demonstrated to be more accurate in representing the topography than previous global DEMs^63^.

Climate predictors (temperature, annual precipitation, aridity index, snow cover fraction) were averaged (mean) within the direct contributing upstream drainage area of each GRIT reach using the CA method. Here, we use the MSWX temperature and precipitation data from 1979-2022 (spatial resolution of 0.1°) to compute the annual mean temperature and annual mean precipitation^64^. The temperature variation predictor (Temp-range) was calculated as the monthly maximum temperature minus the monthly minimum temperature, averaged across all months.

Water table depth and soil moisture data were obtained from SOIL-WATERGRIDS^65^, monthly data at a spatial resolution of 0.25° over the period 1970–2014. We used the mean depths of the highest and lowest monthly water tables as predictors, namely Water Table Depth-High (WTDH) and Water Table Depth-Low (WTDL). The monthly mean soil moisture was extracted at depth ranges of 0-30 cm, 30-60 cm, and 60-100 cm in the root zone based on all months of the period 1970-2014 (predictors: SM_0-30, SM_30-60, SM_60-100). Additionally, we used the mean soil thickness over the period 1970-2014^66^. The above predictors were computed using the CA method.

The aridity index^67^ (“Global Aridity Index and Potential Evapotranspiration Database - Version 3”, Global-AI_PET_v3) has a spatial resolution of 30” ( ~ 1 km). Time-averaged values of the aridity index from 1970 to 2000 were computed over each GRIT river catchment using the CA method. Monthly 5, 50, and 95-, percentile snow-cover fractions at approximately 1 km spatial resolution, derived from MODIS daily Snow observations, were obtained for 2000–2019^68^. For each percentile, we calculated the temporal mean across the monthly values and used the resulting three long-term mean snow-cover fractions as predictors. The CA value over each GRIT reach catchment was then used as the predictor. The Köppen-Geiger climate category^38^ is also used as a predictor, where the climate region is defined by the most common Köppen value within the catchment.

Vegetation in the catchment controls evaporation and infiltration rates, influencing stream discharge and the resulting river channel network^69,70^. Here, we used the Leaf Area Index (LAI) as a proxy for vegetation in the catchment. LAI tracks the one-sided green leaf area per unit of ground surface area, while the Fraction of Absorbed Photosynthetically Active Radiation (FAPAR) quantifies the solar radiation absorbed by plants within the photosynthetically active radiation spectral region. Gridded daily data from 1981 to 2022 of LAI and FAPAR were accessed from NOAA Climate Data Record of Advanced Very High Resolution Radiometer^71^. Both datasets have a spatial resolution of 0.05°. The mean value over 1981-2022 was computed for each GRIT river catchment. The CA value over each GRIT reach catchment was used as the predictor.

Urbanisation may also affect runoff rates, and therefore, we also used the percentage of impervious area as a predictor. We obtained urban areas from the land cover classification gridded map produced by the Copernicus Climate Change Service from multiple satellite images acquired for the year 2010, then calculated the fraction of urban area in each grid cell, at a spatial resolution of 0.25°^72^. Lastly, we took the CA value of these data as the predictor.

### Random forest model

Benefiting from a bootstrap aggregating technique, RF models can assimilate information from a large number of predictors to estimate the target while limiting overfitting. This is achieved by creating multiple regression models from different subsets of the training samples and combining their outputs to compute the estimation^73,74^. The importance score is defined as the total decrease in node impurity resulting from splitting the predictor variables, averaged over all trees^73^, measured by the residual sum of squares. The importance indicates the relative significance of each predictor for predicting the target variable (Supplementary Fig. S7). We used the *scikit-learn* package (version 1.1.2) in Python^75^ to construct our RF model.

Eighty percent of the observations were assigned to the training set using stratified random sampling, with stratification factors defined by discharge magnitude (binned by quartiles of log₁₀(Q_BF_)) and climate region. This ensured that the training data retained a proportional representation of both Q_BF_ magnitude and climate region relative to the full observation dataset. We achieved this by using the *stratify* parameter in *scikit-learn* package. Five-fold cross-validation was used to tune the model parameters (*max_features*, *min_samples_leaf*, *n_estimators*), by maximising the mean R^2^ value across the validation folds. We calculated error metrics for the training, post-tuning cross-validation, and test datasets, as well as separately within each climate region. For the post-tuning cross-validation, performance was evaluated on validation folds generated independently from those used for hyperparameter tuning.

To assess robustness, the entire procedure was repeated 10 times with different random seeds, resulting in different training and test splits. The mean error metrics for training, cross-validation, and testing are reported in Table S2. Five-fold cross-validation results from one of the post-tuning model runs are shown in Supplementary Fig. S17. The model achieving the highest performance across these runs was then used to estimate Q_BF_ for the ~2.87 million km of river reaches in GRIT, with its associated error metrics reported in the main text and Supplementary Figs. S7, 8. This process can be reproduced using the provided code.

### Flood frequency analysis

In order to estimate the return period of Q_BF_, long time series of discharge records are required. We applied a three-step process to obtain the Q_BFobs-RP_, Q_BFest-RP_ and the 2-year return period flow (Q_RP2_). We used a global dataset of gauging stations to identify the discharge records associated with Q_BFobs_ and Q_BFest_. We first selected gauging stations with high-quality discharge records, defined as having at least 10 complete years of data, each with at least 90% of the daily discharge values available. We then paired the selected gauging stations with the Q_BFobs_ by the gauging site number, and with the Q_BFest_ based on the snapping approach mentioned in the bankfull discharge reference data section above. Our initial global gauging station dataset includes a total of 22,538 stations collected from various agencies for the period 1950-–2021^3^. Eventually, we paired 1818 Q_BFobs_ and 9058 Q_BFest_ values with the qualified discharge records (1545 overlapping sites had both Q_BFobs_ and Q_BFest_).

We estimated the Q_BFobs-RP_, Q_BFest-RP_, and Q_RP2_ using Flood Frequency Analysis (FFA). FFA establishes the relationship between the flood magnitude and frequency of occurrence (or return period). FFA is typically used to estimate the flood quantiles (such as the 2-year event or more extreme events). To obtain the relationship between flood magnitude and frequency of occurrence, flood events are first selected from a long-time series of discharge records, and then the probability of occurrence is estimated based on the statistics of selected flood events. Given that rivers may reach bankfull more frequently than once per year on average, we selected the flood events based on the Peak-Over-Threshold (POT) approach^76^ using the *floodnetRfa* R package (https://github.com/floodnetProject16/floodnetRfa).

In the POT method, the user must determine the appropriate threshold and the rule for separating adjacent flood events to identify independent flood events^77^. Here, to be able to estimate the return period of bankfull discharge, the event identification threshold must be smaller than Q_BF_. Rather than manually identifying this threshold, we systematically assessed four different thresholds, namely 10%, 25%, 50%, and 75% of the bankfull discharge value. We identified independent flood events using the peak-separation criteria of the US Water Resources Council, as described by Lang et al^78^. We then employed a Locally Weighted Scatterplot Smoothing (Loess) curve^79^ to describe the relationship between the recurrence probability and the identified flood events for each threshold. Loess is a non-parametric method that fits a smooth curve to the data and is particularly useful for capturing the underlying patterns of non-linear relationships. Here, we used a Loess span of 0.5, which provides a moderate degree of smoothing. Lastly, we obtained four recurrence interval estimates for Q_BFobs-RP_ and Q_BFest-RP_ corresponding to the four POT thresholds.

Our analysis of the four bankfull discharge return periods at each gauging station revealed that the four return-period estimates diverged most strongly at sites where Q_BF_ is rarely exceeded (return periods >10 years) (Supplementary Figs. S18, 19). We thus kept only those sites where the estimated Q_BF_ return period was robust to the choice of the peak threshold, with no more than 50% variation among the four estimated Q_BF-RP_ from the four sensitivity-testing thresholds above. We chose this value of 50% variation to reduce uncertainty and avoid removing all the sites with relatively large return periods. Details of the relationship between the variation in the estimated return period and the number of events exceeding Q_BF_ can be found in Supplementary Figs. S18, 19. We averaged the Q_BF-RP_ estimated from the four sensitivity thresholds to obtain the final return period. Finally, we obtained the Q_BFest-RP_ for 8519 sites, among which 1545 sites also had Q_BFobs-RP_ (Fig. 2). The choice of span parameters and fitting methods has a negligible impact on the estimated return period (Supplementary Figs. S13, 14). We employed the Kruskal-Wallis H test to assess whether bankfull discharge return periods differed among climate regions (null hypothesis: equal distributions across groups), followed by Dunn’s test for pairwise comparisons (Python *scipy* package).

Using the same method as above, we estimated the discharge at the 2-year return period. The mean discharge values from the four sensitivity-testing thresholds were employed as the final Q_RP2_. We calculated the bias of the 2-year return period of discharge relative to the Q_BFobs_ at 1725 sites (Fig. 4).

## Supplementary information

Supplementary Information

Transparent Peer Review file

## Data Availability

The GQBF data is publicly available at Zenodo: https://doi.org/10.5281/zenodo.13855371. The out-of-range flags are available at https://doi.org/10.5281/zenodo.13855370. Access to key data used in this study is described below. The GRIT network (river vectors with associated drainage area and sinuosity attributes) can be downloaded from https://zenodo.org/records/11219313. Other river attributes can be downloaded from the GRIT-ADB https://doi.org/10.5281/zenodo.19363178. Access to raw data used to generate the predictors is described below. FABDEM can be downloaded from https://data.bris.ac.uk/data/dataset/25wfy0f9ukoge2gs7a5mqpq2j7. GLOFAS version 4 can be downloaded from https://cds.climate.copernicus.eu/cdsapp#!/dataset/cems-glofas-historical?tab=form. The GRWL river data can be downloaded from https://zenodo.org/records/1297434. The water occurrence data can be downloaded from https://global-surface-water.appspot.com/download. The temperature and precipitation data can be downloaded from https://www.gloh2o.org/mswx/. LAI and FAPAR can be downloaded from https://www.ncei.noaa.gov/products/climate-data-records/leaf-area-index-and-fapar. Urban coverage data can be downloaded from https://cds.climate.copernicus.eu/cdsapp#!/dataset/satellite-land-cover?tab=overview. Snow cover fraction data can be downloaded from https://zenodo.org/records/5774954. The soil grid (water table depth, soil moisture) is obtained from https://zenodo.org/records/4997453. The soil thickness can be downloaded from https://daac.ornl.gov/SOILS/guides/Global_Soil_Regolith_Sediment.html#:~:text=6)%20average_soil_and_sedimentary%2Ddeposit_thickness.tif,upland%20hillslopes%20and%20valley%20bottoms.

## Code availability

The code for constructing and evaluating the random forest model and for estimating the return period of bankfull discharge and the 2-year return period discharge from daily river discharge records using the peak-over-threshold approach is available at https://doi.org/10.5281/zenodo.21451739.
