# Author Correction: Global estimation of bankfull river discharge reveals distinct flood recurrences across climate zones

**DOI:** 10.1038/s41467-026-78092-w

**Published:** 2026-09-25

**Authors:** Yinxue Liu, Michel Wortmann, Laurence Hawker, Jeffrey Neal, Jiabo Yin, Marcus Suassuna Santos, Bailey Anderson, Richard Boothroyd, Andrew Nicholas, Greg Sambrook Smith, Philip J. Ashworth, Hannah Cloke, Solomon Gebrechorkos, Julian Leyland, Boen Zhang, Ellie Vahidi, Helen Griffith, Pauline Delorme, Stuart James McLelland, Daniel R. Parsons, Stephen E. Darby, Louise Slater

**Affiliations:** 1 School of Geography and the Environment, University of Oxford, South Parks Road, Oxford, UK; 2 Geography and Environment, Loughborough University, Loughborough, UK; 3 European Centre for Medium-Range Weather Forecasts (ECMWF), Shinfield Park, Reading, UK; 4 School of Geographical Sciences, University of Bristol, University Road, Bristol, UK; 5 State Key Laboratory of Water Resources Engineering and Management, Wuhan University, Wuhan, China; 6 Department of Hydrology, Geological Survey of Brazil – SGB/CPRM, Brasilia, Brazil; 7 WSL Institute for Snow and Avalanche Research SLF, Davos Dorf, Switzerland; 8 Institute for Atmospheric and Climate Science, ETH Zurich, Zurich, Switzerland; 9 Department of Geography and Planning, School of Environmental Sciences, University of Liverpool, Liverpool, UK; 10 Geography, Faculty of Environment, Science and Economy, University of Exeter, Exeter, UK; 11 School of Geography, Earth and Environmental Sciences, University of Birmingham, Birmingham, UK; 12 Department of Earth Science and Environmental Change, University of Illinois, Urbana-Champaign, IL USA; 13 Department of Meteorology, University of Reading, Reading, UK; 14 Department of Geography & Environmental Science, University of Reading, Reading, UK; 15 School of Geography and Environmental Sciences, University of Southampton, Southampton, UK; 16 JBA Consulting, 1 Broughton Park, Old Lane North, Broughton, Skipton, UK; 17 Energy and Environment Institute, University of Hull, Hull, UK; 18 Vice Chancellor’s Office, Loughborough University, Loughborough, UK

**Keywords:** Natural hazards, Hydrology

Correction to: *Nature Communications* https://doi.org/10.1038/s41467-026-76433-3, published online 18 August 2026

In the version of this article initially published, the first name of **Philip J. Ashworth** was spelled incorrectly (Philp) and is now amended in the HTML and PDF versions of the article.

