# Supplementary material for: Global estimation of bankfull river discharge reveals distinct flood recurrences across climate zones
Source: Nat Commun. 2026 Aug 18;17:8179. doi: 10.1038/s41467-026-76433-3 (PMC13487230; doi:10.1038/s41467-026-76433-3)
Supplement: Supplementary file 1 [file 41467_2026_76433_MOESM1_ESM.pdf]

Supplementary Materials for

Global Estimation of River Bankfull Discharge Reveals Distinct Flood Recurrences Across Climate Zones

Yinxue Liu[1,2]*†, Michel Wortmann[1,3]*†, Laurence Hawker[4], Jeffrey Neal[4], Jiabo Yin[1,5], Marcus Suassuna Santos[6], Bailey Anderson[7,8], Richard Boothroyd[9], Andrew Nicholas[10], Greg Sambrook Smith[11], Philip J. Ashworth[10,12], Hannah Cloke[13,14], Solomon Gebrechorkos[1,15], Julian Leyland[15], Boen Zhang[1], Ellie Vahidi[10], Helen Griffith[16], Pauline Delorme[17], Stuart James McLelland[17], Daniel R Parsons[2,18], Stephen E. Darby[15], Louise Slater[1†]

[1]School of Geography and the Environment, University of Oxford, South Parks Road, Oxford, OX1 3QY, UK,

[2]Geography and Environment, Loughborough University, Loughborough, LE11 3TU, UK,

[3]European Centre for Medium-Range Weather Forecasts (ECMWF), Shinfield Park, Reading, RG2 9AX, UK,

[4]School of Geographical Sciences, University of Bristol, University Road, Bristol BS8 1SS, UK,

[5]State Key Laboratory of Water Resources Engineering and Management, Wuhan University, Wuhan, China,

[6]Department of Hydrology, Geological Survey of Brazil – SGB/CPRM, Brasilia, Brazil,

[7]WSL Institute for Snow and Avalanche Research SLF, Davos Dorf, Switzerland,

[8]Institute for Atmospheric and Climate Science, ETH Zurich, Zurich, Switzerland,

[9]Department of Geography and Planning, School of Environmental Sciences, University of Liverpool, Liverpool, UK,

[10]Geography, Faculty of Environment, Science and Economy, University of Exeter, Exeter, EX4 4RJ UK,

[11]School of Geography, Earth and Environmental Sciences, University of Birmingham, Birmingham, B15 2TT, UK,

[12]Department of Earth Science and Environmental Change, University of Illinois Urbana-Champaign, IL, 61801, USA,

[13]Department of Meteorology, University of Reading, Reading, UK,

[14]Department of Geography & Environmental Science, University of Reading, Reading, UK,

[15]School of Geography and Environmental Sciences, University of Southampton, Southampton, UK,

[16]JBA Consulting, 1 Broughton Park, Old Lane North, Broughton, Skipton, North Yorkshire, BD23 3FD, UK,

[17]Energy and Environment Institute, University of Hull, Hull, UK,

[18]Vice Chancellor's Office, Loughborough University, Loughborough, UK

Correspondence to: Yinxue Liu (Yinxue.liu@ouce.ox.ac.uk; Yinxue.liu@outlook.com); Michel Wortmann (michel.wortmann@ecmwf.int) and Louise Slater (louise.slater@ouce.ox.ac.uk)

Contents of this file

This file contains 19 supplementary figures, 2 supplementary tables, and texts S1 and S2.

- Text S1. Sources of the bankfull discharge observation dataset.
- Fig. S1. Spatial distribution of the observed bankfull discharge from 9 sources.
- Fig. S2. Example of bankfull discharge estimation at one USGS gauging station.
- Fig. S3. Example of bankfull discharge estimation in Colombia at one gauging station.
- Fig. S4. Examples of bankfull discharge estimation in Brazil at one gauging station.
- Fig. S5. Observed bankfull discharge density distribution for each of the Global RIver Topology (GRIT) global basins (60 in total).
- Fig. S6. Cumulative density distribution of predictors used in the Random Forest model for the training samples and the global river reaches.
- Fig. S7. Cross-validation results of the Random Forest model and importance ranking of the predictors.
- Fig. S8. Training and testing results of the Random Forest model in four climate regions.
- Fig. S9. Error density distribution together with the magnitude of the estimated bankfull discharge and the record length of the streamflow data.
- Fig. S10. Random Forest performance (R^2) for each domain, using each individual domain as the testing set and all other domains as the training set.
- Fig. S11. An example of Q_{BFest} in the upper Mississippi river.
- Fig. S12. Cumulative density distribution of upstream junction counts for all the rivers (~2.87 million km in length) and for the out-of-range rivers.
- Fig. S13. Variation of the estimated return period for observed bankfull discharge with different span parameters of the Loess method.
- Fig. S14. The estimated return periods of observed bankfull discharge using the Generalized Pareto distribution method.
- Fig. S15. Distribution of return period of bankfull discharge across climate regions, derived from two observation sources in the USA.
- Fig. S16. Example of the water masks at three occurrence values from Landsat surface water occurrence data1 in the upper Mississippi River.
- Fig. S17. An example of five-fold cross-validation result with tuned parameter set and error metrics annotated.
- Fig. S18. Relationship between the estimated return period of the observed bankfull discharge (Q_{BFobs}) and the number of exceedance events for 1,818 gauging stations.
- Fig. S19. Relationship between the estimated return period of the estimated bankfull discharge (Q_{BFest}) and the number of exceedance events for 9,058 gauging stations.
- Table S1. Details of the predictors used in the Random Forest model.
- Table S2. Averaged error metrics of training, cross-validation, and test data as well as that of each climate region of the Random Forest model.
- Text S2. Equations of the error metrics used to assess Random Forest model performance.

Text S1

The bankfull discharge observations are a compilation of nine different datasets (Fig. S1). The observations are of two types: six existing bankfull discharge datasets, sourced primarily from the literature, and three bankfull discharge datasets computed from cross-sectional river measurements. The six pre-existing bankfull discharge datasets include: *UK CEH*, *USA2 Literature*, *Europe Literature*, *USA3-Northeast*, *USA4-Alaska*, *China Literature* (shown in the legend of Fig. S1). All the literature-based references are included in the reference list of the main manuscript.

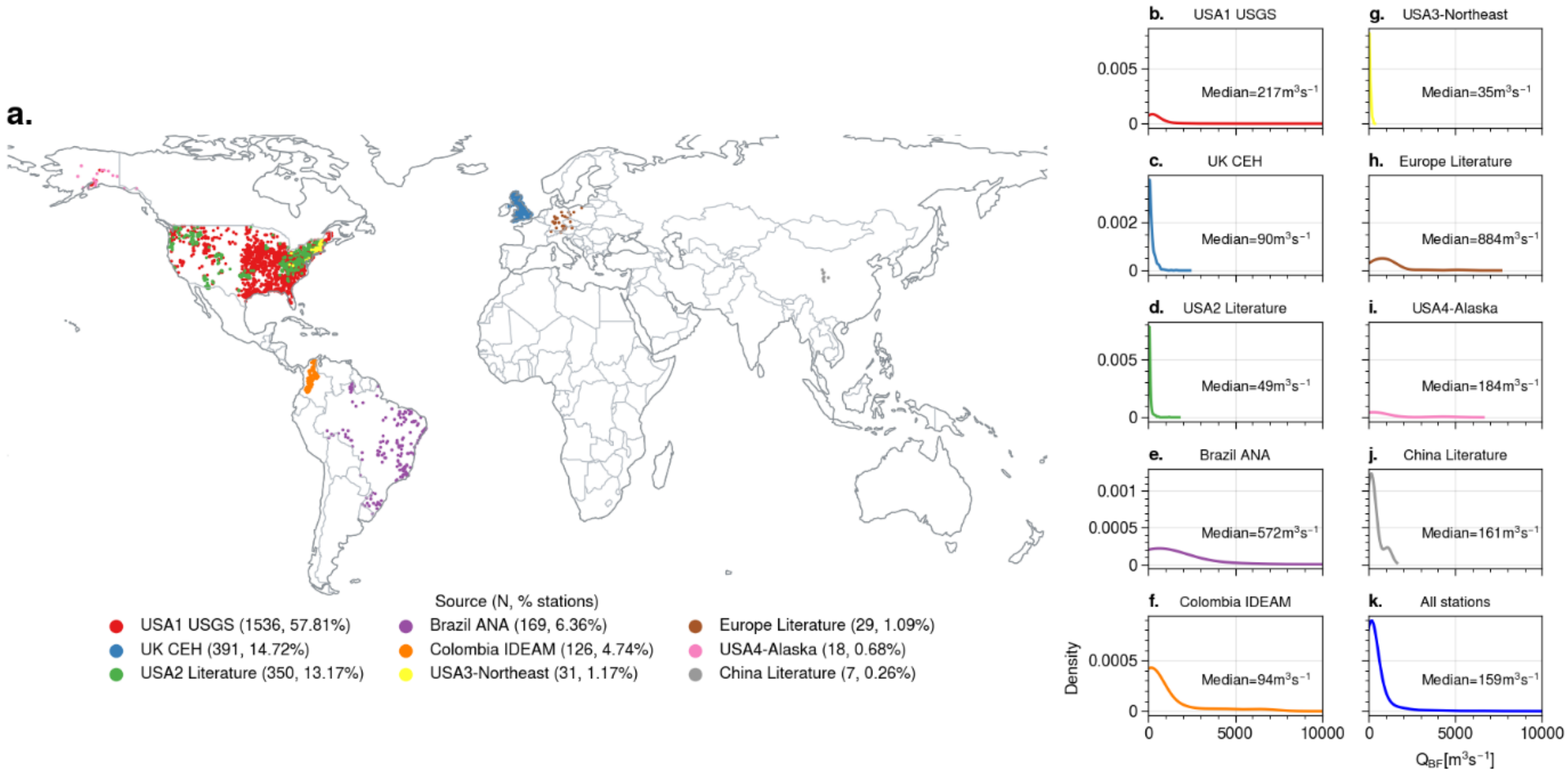

Fig. S1. Location of observed bankfull discharge at 2,657 stations (panel a) and density distribution of the bankfull discharge for each source (panels b-f), and for all stations (panel k). Colour is assigned to differentiate the data sources. In panel a, numbers in brackets indicate the number of stations from each source. In panel b-k, the median value for each source is annotated.

The *UK CEH* data was obtained by personal communication from the UK National River Flow Archive – UK Centre for Ecology and Hydrology, https://nrfa.ceh.ac.uk/.

The *USA2 Literature* data was sourced from Bieger et al. (2015)[30]. We obtained the data from http://swat.tamu.edu/publications/. Data description of the bankfull discharge data can be found in the paper available at https://doi.org/10.1111/jawr.12282.

The *Europe Literature* data was sourced from Schneider et al. (2011)[27], available at https://doi.org/10.1016/j.jhydrol.2011.08.004, Table 5 (column Q_{bf} obs). It reports observed bankfull discharge at 67 locations in Europe. We kept the 30 stations whose station id matched with the longitude and latitude information recorded in the Global Runoff Data Centre (GRDC) database (downloaded from https://portal.grdc.bafg.de/applications/public.html?publicuser=PublicUser#dataDownload/Home).

The *USA3-Northeast* data is a compilation of values curated by Mr Gardner Bent, based on a series of reports listed in https://pubs.usgs.gov/sir/2013/5155/. We retained 48 sites that matched the station ids obtained from the United States Geological Survey (USGS), available at https://waterdata.usgs.gov/nwis/inventory.

The *USA4-Alaska* data is from Anderson (2023) and can be downloaded from https://doi.org/10.5281/zenodo.7920063. It reports the bankfull discharge for 45 Alaskan rivers based on observed streamflow and water level measured in the field, from aerial photographs, satellite imagery, or field surveys (pers. communication).

The *China Literature* data was sourced from Li et al. (2021), available at https://doi.org/10.3390/rs13142650, Table 2. We retained the bankfull discharge values in 22 hydrological stations (12 mainstem, 10 tributary) located in the upper Yellow River from this source.

In addition to the above six existing bankfull discharge datasets, three additional datasets were computed for the manuscript and are described below. Since direct measurements of bankfull discharge are not available, all three datasets were derived from cross-sectional measurements of the river channel.

The *USA1 USGS* bankfull discharge dataset was derived using the approach described in Slater et al. (2015)[47]. This approach employs cross-section measurements of stage and discharge from the USGS to compute a curve at each site, and then intersects the curve with the flood stage measurement to extract the estimated bankfull discharge. The flood stages were downloaded from the National Oceanic and Atmospheric Administration's National Weather Service (NWS) at https://water.weather.gov/ahps/download.php. The NWS defines "action" flood stage as "an established gauge height for a given location above which a rise in water surface level begins to create a hazard to lives, property, or commerce". We make the assumption that this is the closest available measurement of bankfull (see Figure S2). Cross-sectional measurements were downloaded from the USGS National Water information system at https://waterdata.usgs.gov/nwis/measurements?search_criteria=huc2_cd&submitted_form=introduction. The channel measurements were filtered to retain only measurements later than 1 January 1980. We retained the single longest channel measurement location ('chan_nu') at each site. We only kept sites with at least 20 measurements over at least 10 years. The stage-discharge rating curve at each site was fitted using a Locally-weighted scatterplot smoothing curve (Loess) in R with a span of 0.2. By intersecting this curve with the National Weather Service's estimate of flood stage at each site, we extracted the time-averaged estimate of bankfull discharge at flood stage (Fig. S2). We obtained bankfull discharge values at 1,536 stations from this source. The resulting bankfull discharge data are distributed across the USA (locations are shown in Fig. S1 panel a, b).

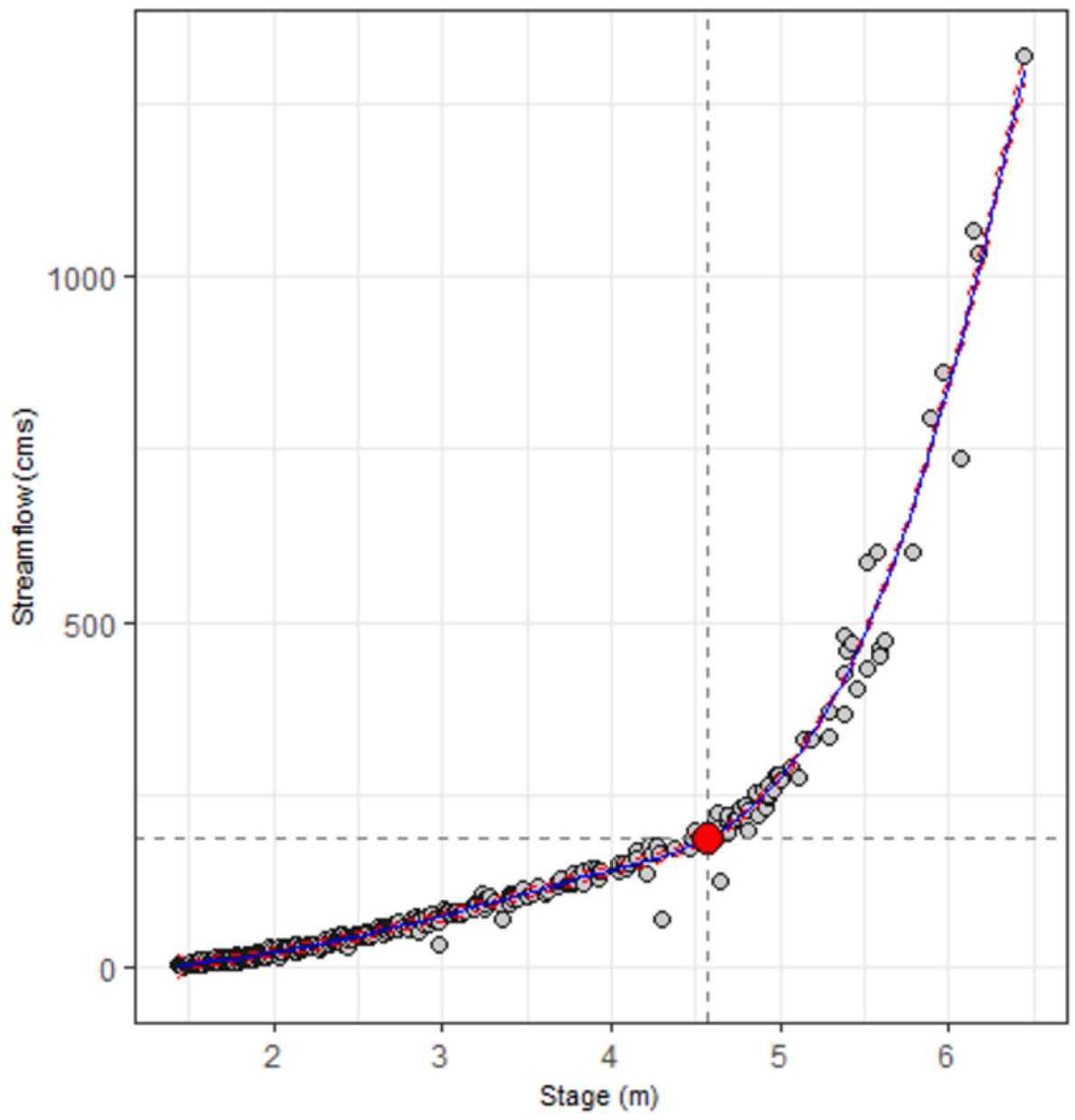

Fig. S2. Example of bankfull discharge computed at one gauge in the USA (site 05453100) which has a National Weather Service minor flood stage of 4.57 m (vertical dashed line) and an estimated bankfull discharge of 187.87 m^3/s (horizontal dashed line). The red circle indicates estimated bankfull discharge (y-axis) at the bankfull stage (x-axis). The blue line indicates a Locally-weighted scatterplot smoothing (Loess) curve fitted to the stage-discharge measurements over the full historical record (grey circles), with the red dashed lines showing the upper and lower bounds of the 95% confidence band for the Loess fit.

The *Colombia IDEAM* bankfull discharge dataset was produced using the following method. Multiple cross-section measurements were obtained for each gauging station from the Colombian Institute of Hydrology, Meteorology and Environmental Studies (IDEAM). The measurements consist of the river channel cross-sectional area, discharge and the date when the measurement was conducted. We took the discharge corresponding to the maximum cross-section area (Qmax.cross_section) as the bankfull discharge as long as the flow was clearly within the banks (Fig. S3). Outliers were removed following the process described below. The cross-sectional discharge values (Qmax.cross_section) were compared with gauged discharge (Qmax.gauge) (maximum discharge within ±3 days of the date of the cross-sectional measurement). We ruled out any stations where the Qmax.cross_section deviated notably from the reported discharge value (Qmax.cross_section >150% Qmax.gauge or Qmax.cross_section <50% Qmax.gauge). We conducted manual checks to ensure that the measured cross-sectional shape had a U shape rather than an inverted omega shape ℧, indicating the flow was most likely within the banks. We kept a total number of 129 stations with valid bankfull discharge values.

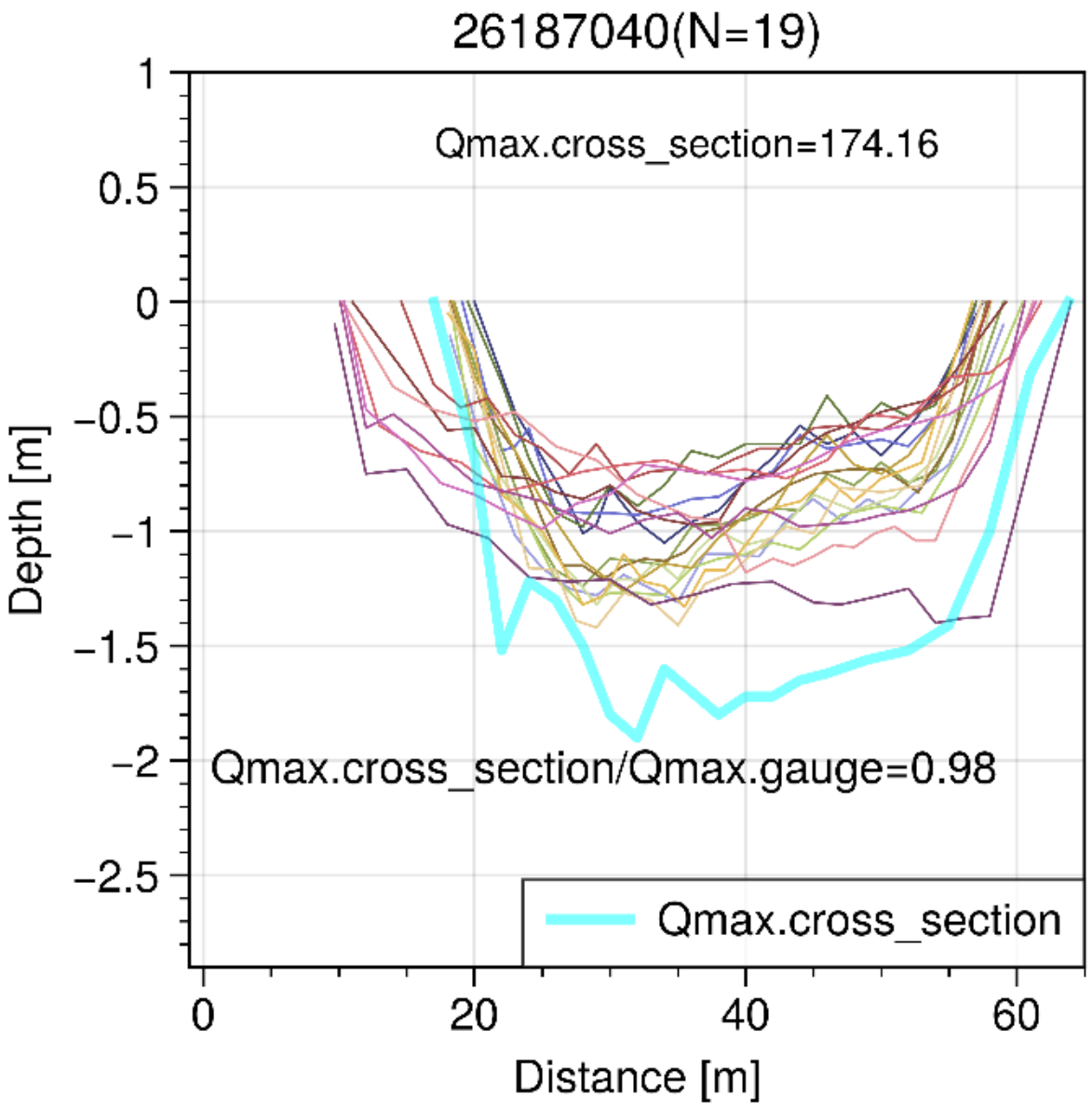

Fig. S3. Example of deriving bankfull discharge for a gauging station in Colombia (site no. 26187040) based on 19 cross-section measurements (coloured). The cross-section with the maximum area (highlighted in cyan) corresponds to the discharge value *Q*max.cross_section, which is taken as the bankfull discharge for this site.

The *Brazil ANA* bankfull discharge dataset was derived by combining cross-sectional measurements of the river channel and the stage-discharge curve at each location. Estimates were derived using a semi-automated approach that involves the evaluation of cross-sectional and stage-discharge measurements. Data were downloaded from the National Hydrometeorological Network of Brazil (https://www.snirh.gov.br/hidroweb/apresentacao, last accessed 5th March, 2024) managed by Brazilian Water and Sanitation Agency (ANA) and largely operated by the Brazilian Geological Survey (SGB/CPRM). Preliminary data cleaning was followed by the analysis of breakpoints in stage-discharge pairings using the 'segmented' R package (Muggeo, 2008), available at https://journal.r-project.org/articles/RN-2008-004/RN-2008-004.pdf (last accessed 10 April, 2026). Between one and three breakpoints were identified at each location. In locations where multiple breakpoints were identified, the one corresponding to the highest flow was considered representative of the bankfull discharge. In certain instances where a reliable breaking point could not be detected, the discharge corresponding to the maximum cross-sectional area was considered bankfull (Fig. S4). Manual checks were performed at all sites to ensure that estimated discharges were consistent with riverbank boundaries. We estimated bankfull flows for 169 stream gauges.

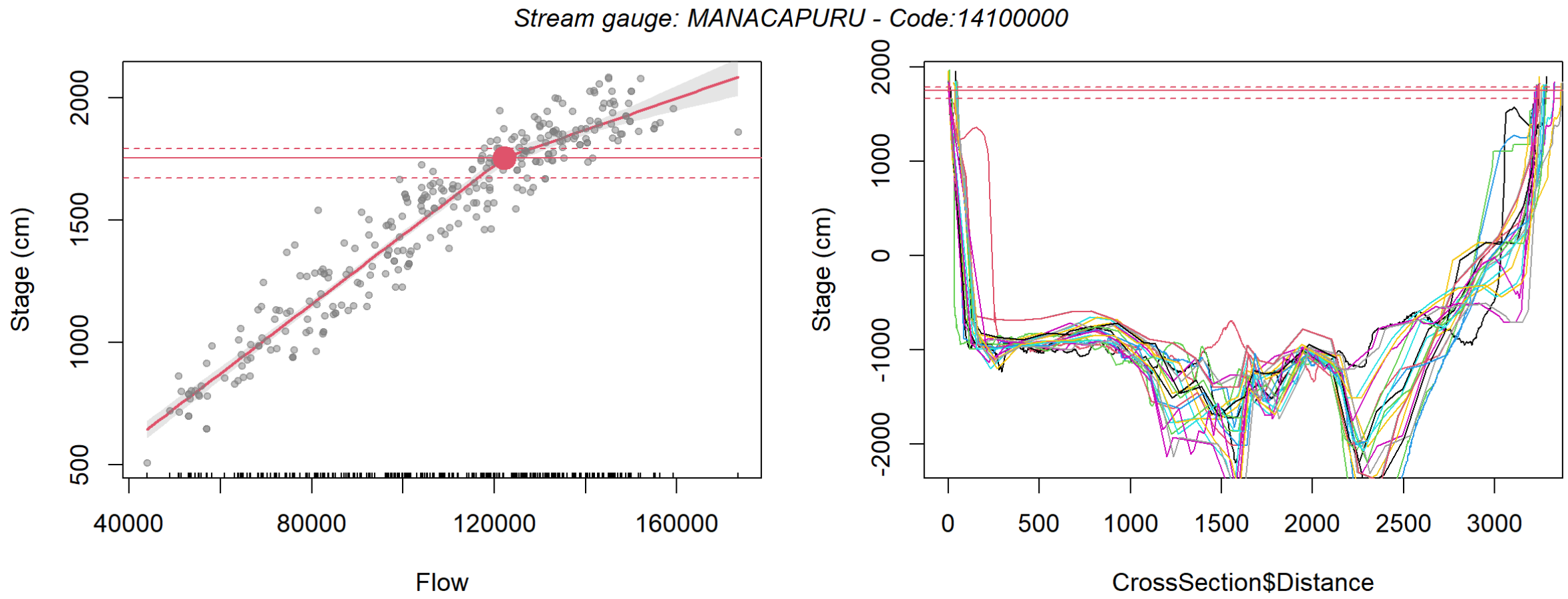

Fig. S4. Examples of deriving bankfull discharge in Brazil (MANACAPURU gauge). Horizontal, solid line (red) marks the identified bankfull stage, horizontal, dashed lines (red) mark the uncertainty interval of the bankfull stage. The right plots show the river cross-sectional measurements at each station.

From these nine data sources, we removed duplicate or erroneous sites following three criteria. First, in locations with duplicate measurements, we retained only one value with the highest confidence. We used the following confidence order: *USA3-Northeast* > *USA1 USGS* > *USA2 Literature* > *USA4-Alaska*. This order was decided based on expert knowledge considering the source data and the measuring methods. Second, we removed any sites located further than 1 km from the Global RIver Topology (GRIT) river reaches and any sites where the reported drainage area deviated more than 20% from the drainage area of the paired GRIT river reach. Lastly, we removed sites with bankfull discharge smaller than 10 m^3s^{-1} (given the constraints in the Random Forest predictors in representing small rivers, such sites are challenging to estimate). After applying these filtering procedures, we retained the bankfull discharge observations for 2,657 river reaches (Fig. S1).

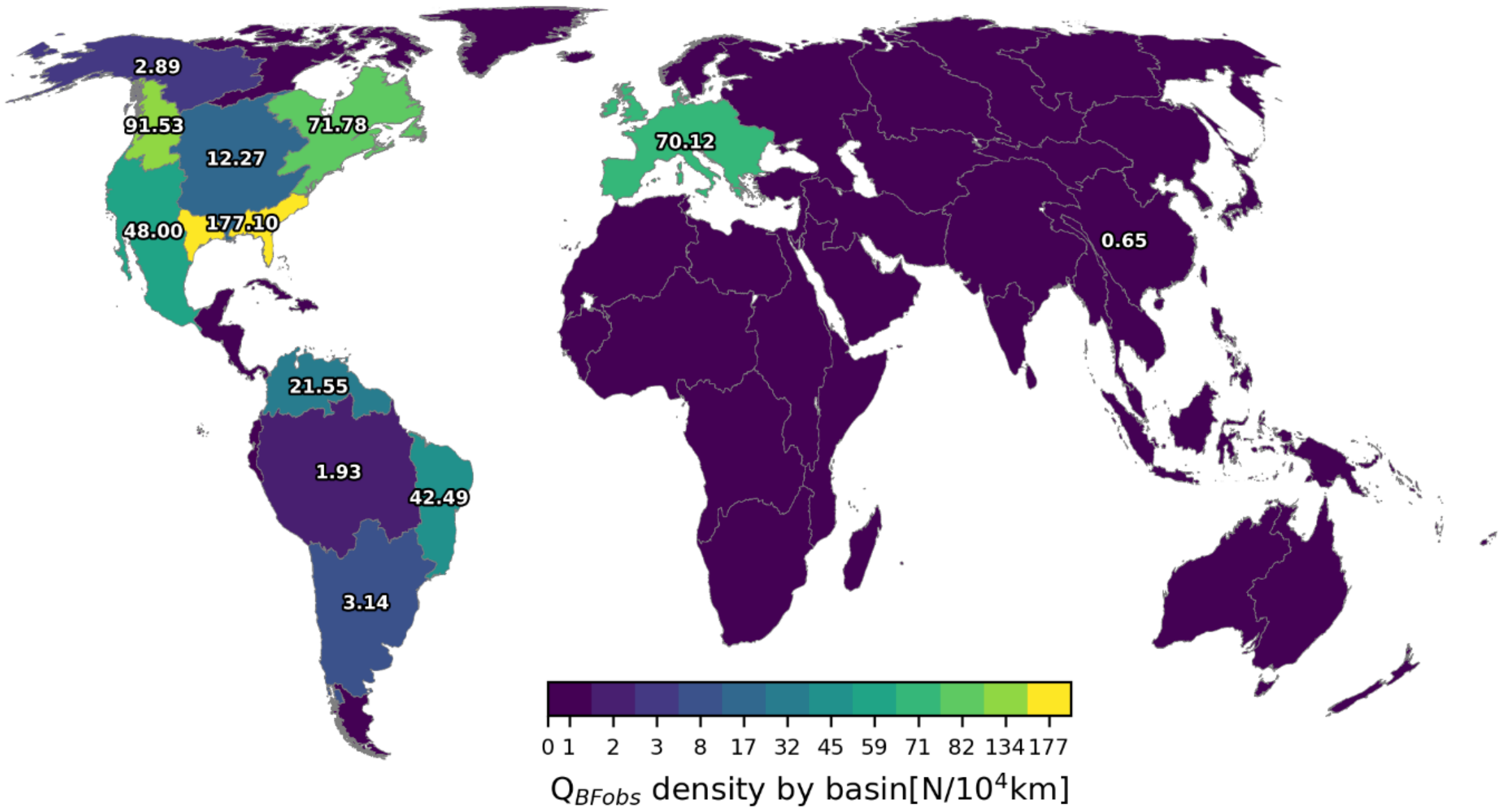

Fig. S5. Observed bankfull discharge density within the Global RIver Topology (GRIT) aggregated basins, showing the 12 out of 60 basins with observed bankfull discharge data, colour coded by density, which is the number of observations per 10,000 km within each basin.

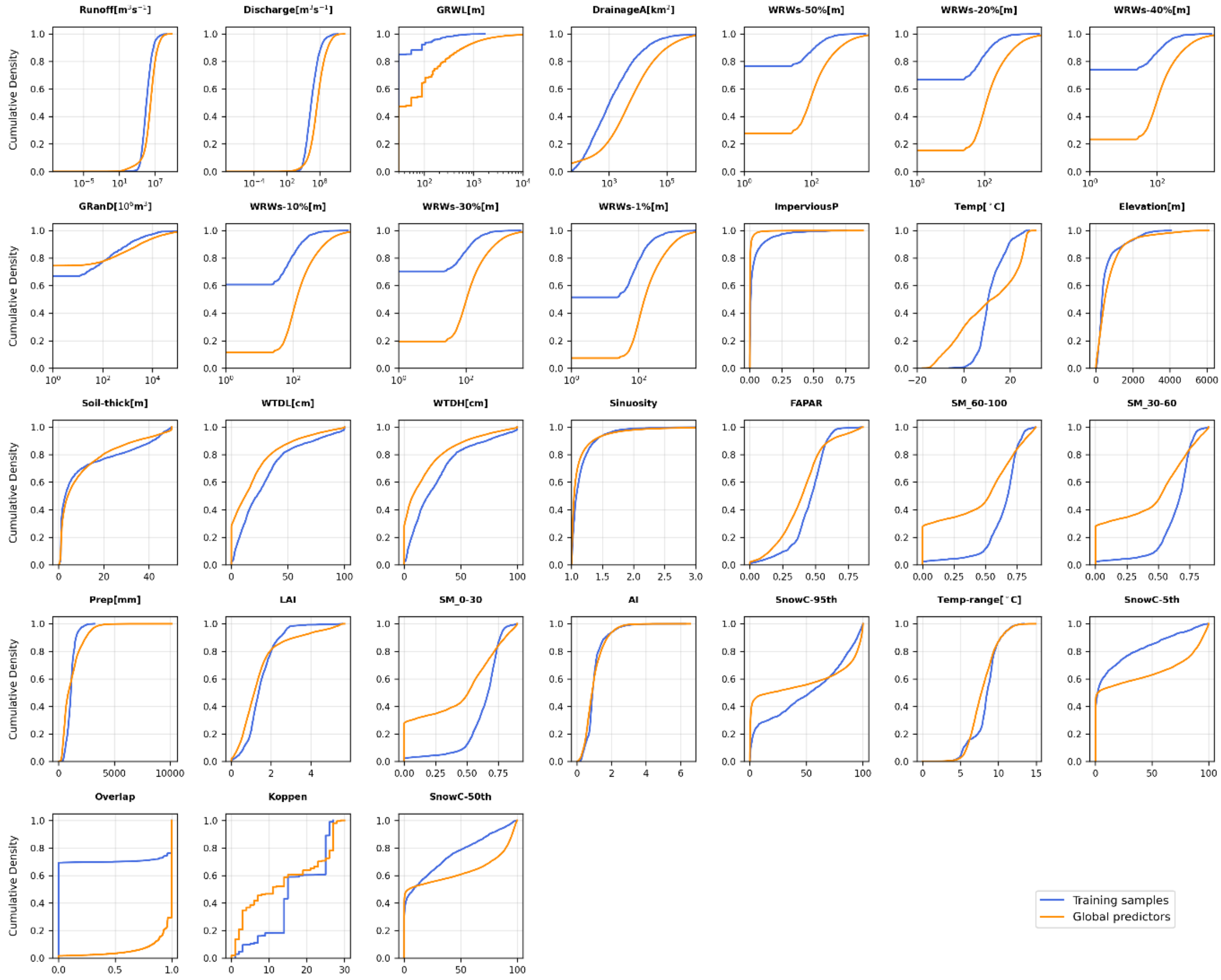

Fig. S6. Empirical cumulative distributions of predictors (panel titles) used in the Random Forest model for training samples (blue lines), and the global river reaches (orange lines). Discharge and Runoff are re-accumulated from GLOFAS v4.0 reanalysis; GRWL: Global River Width from Landsat; DrainageA: Drainage area; WRWs-50%: Wetted River Width at 50% Landsat-based water occurrence (same for other WRWs-occurrences); GranD: Global Reservoir and Dam database version 1.3; ImperviousP: Impervious Percentage; Temp: Temperature; Elevation: Digital elevation model from Forest And Buildings removed Copernicus DEM (FABDEM); Soil-thick: Soil thickness; WTDL: Water Table Depth (Low); WTDH: Water Table Depth (High); FAPAR: Fraction of Absorbed Photosynthetically Active Radiation; SM_60-100: Soil Moisture at depth 60-100 cm; SM_0-30, SM_30-60 follow the same format; Prep: Precipitation; LAI: Leaf Area Index; AI: Aridity Index; SnowC-95th: MODIS snow cover fraction at 95th percentile (SnowC-5th and SnowC-50th follows the same format); Temp-range: Temperature range (maximum - minimum temperature); Overlap: the overlap ratio between the GRWL water mask and the DEM-derived river; Koppen: the categorical value of Koppen climate. Out-of-range river reaches (width outside 30 – 1,673 m or drainage area outside 50 – 3,425,560 km^2) were excluded.

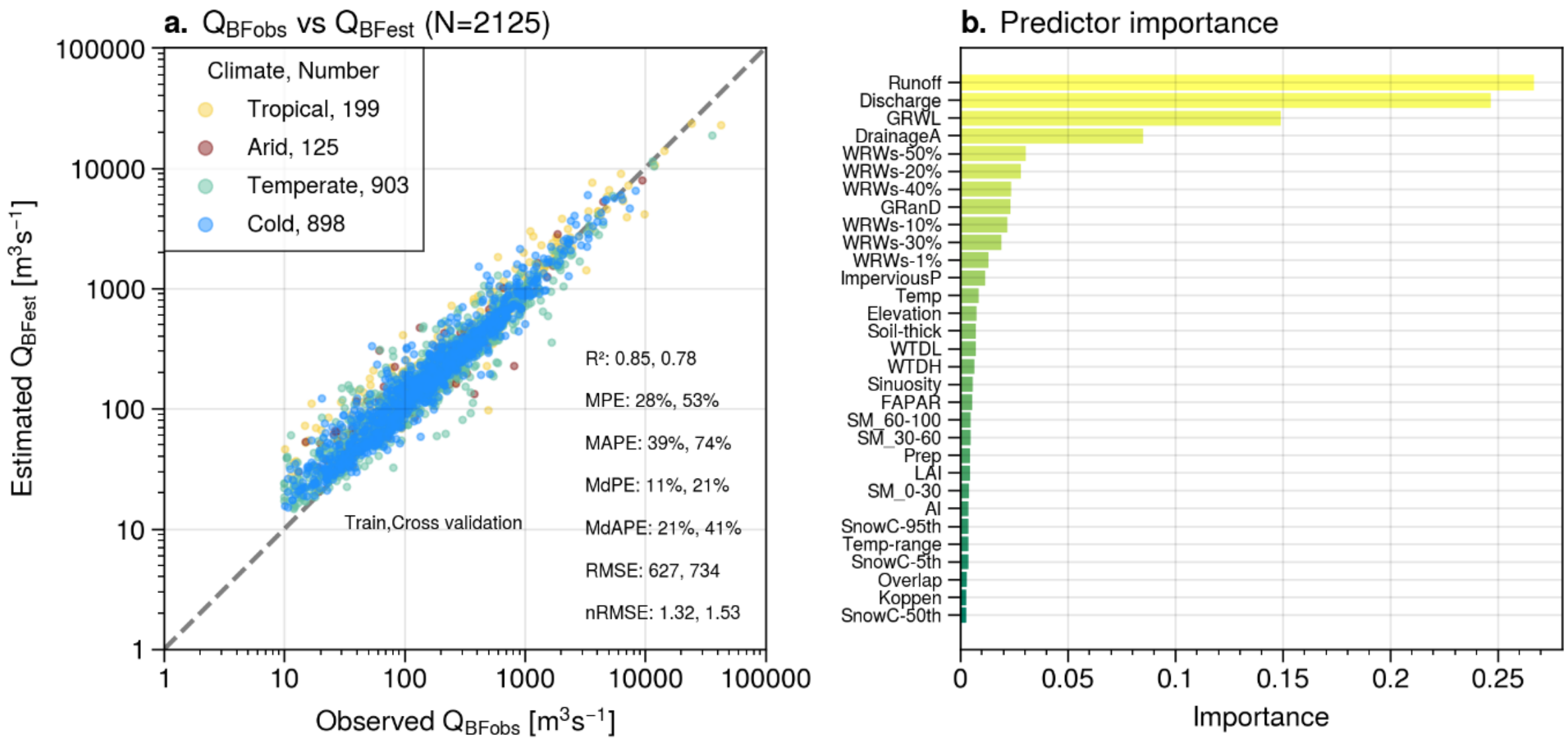

Fig. S7. Cross-validated Random Forest model. a, Scatter plot (log scale) of observed bankfull discharge (Q_{BFobs}) versus estimated bankfull discharge (Q_{BFest}) at 2,125 training Q_{BFobs} locations with train and 5-fold cross-validation metrics annotated. Both axes are log scaled. Legend (upper left) indicates the number of samples in each climate. Colours indicate the four main Koppen climate regions. b, Importance ranking of predictors in decreasing order. The horizontal axis shows the normalized impurity-based importance of each feature (their sum equals 1).

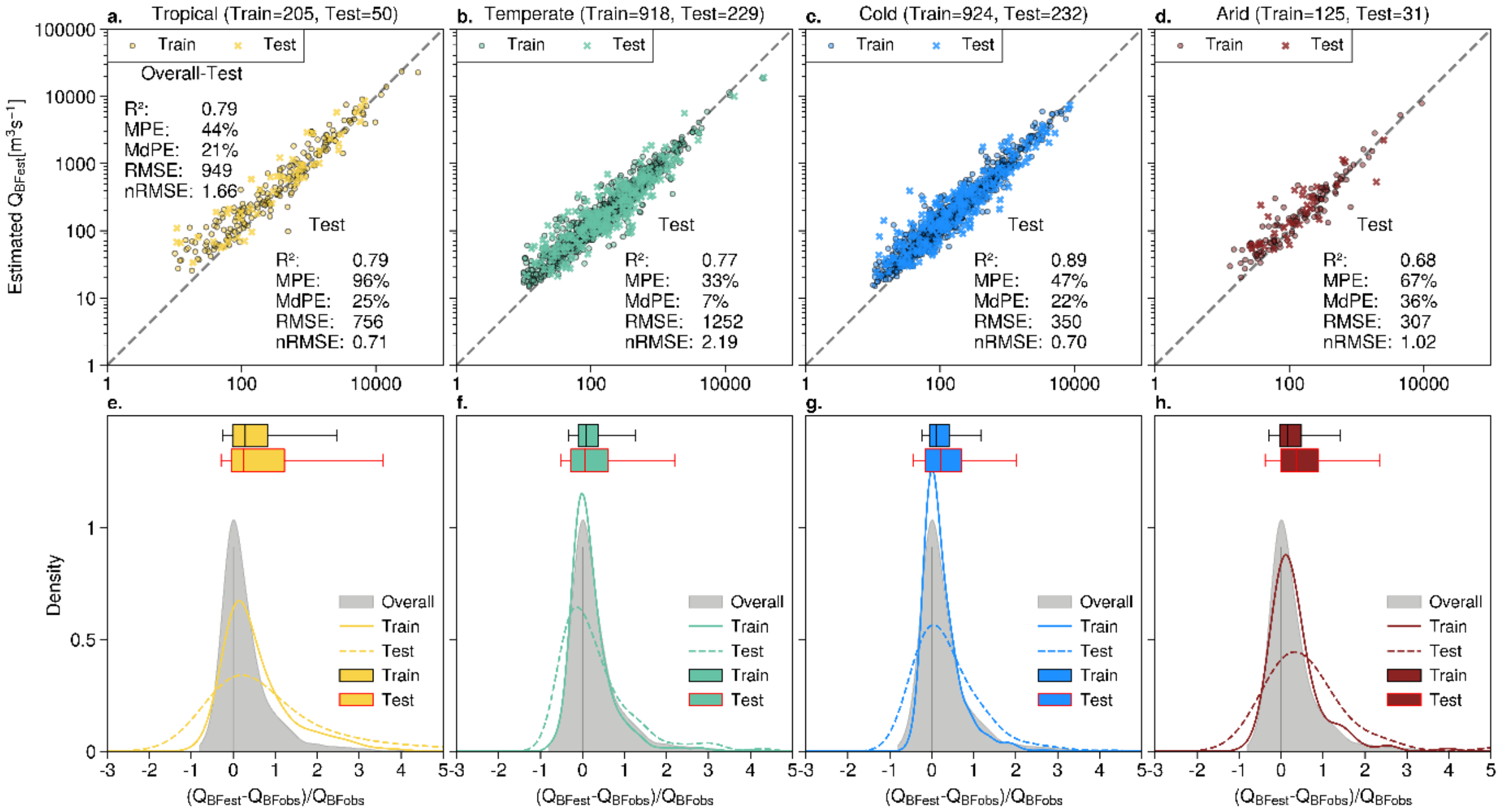

Fig. S8. Performance of the global Random Forest model in the training and testing datasets in four climate regions. a-d, Comparison between observed bankfull discharge (Q_{BFobs}) and estimated bankfull discharge (Q_{BFest}) in the tropical, arid, temperate, and cold regions, respectively (log scale). Error metrics (R^2, Mean Percentage Error: MPE, Median Percentage Error: MdPE, Root Mean Square Error: RMSE, normalized RMSE, equations of these metrics are in Supplementary Text S2) of the overall testing set are annotated in the upper left on panel a, while error metrics of individual climate zones are annotated on the lower right of each panel. Panel captions show the number of training and testing data points in each climate region. e-h, Q_{BFest} error (horizontal axis) distribution (density and boxplot) in the four climate regions, differentiating by training and testing data. Grey shaded area is the overall density distribution (training + testing in all climate regions). Grey vertical line marks the horizontal axis origin. The boxplots share the same horizontal axis as the density plot. The boxplot lines, from left to right, mark the 5th, 25th, 50th, 75th, and 95th percentiles, respectively. Colour indicates the climate region, consistent with panels a-d.

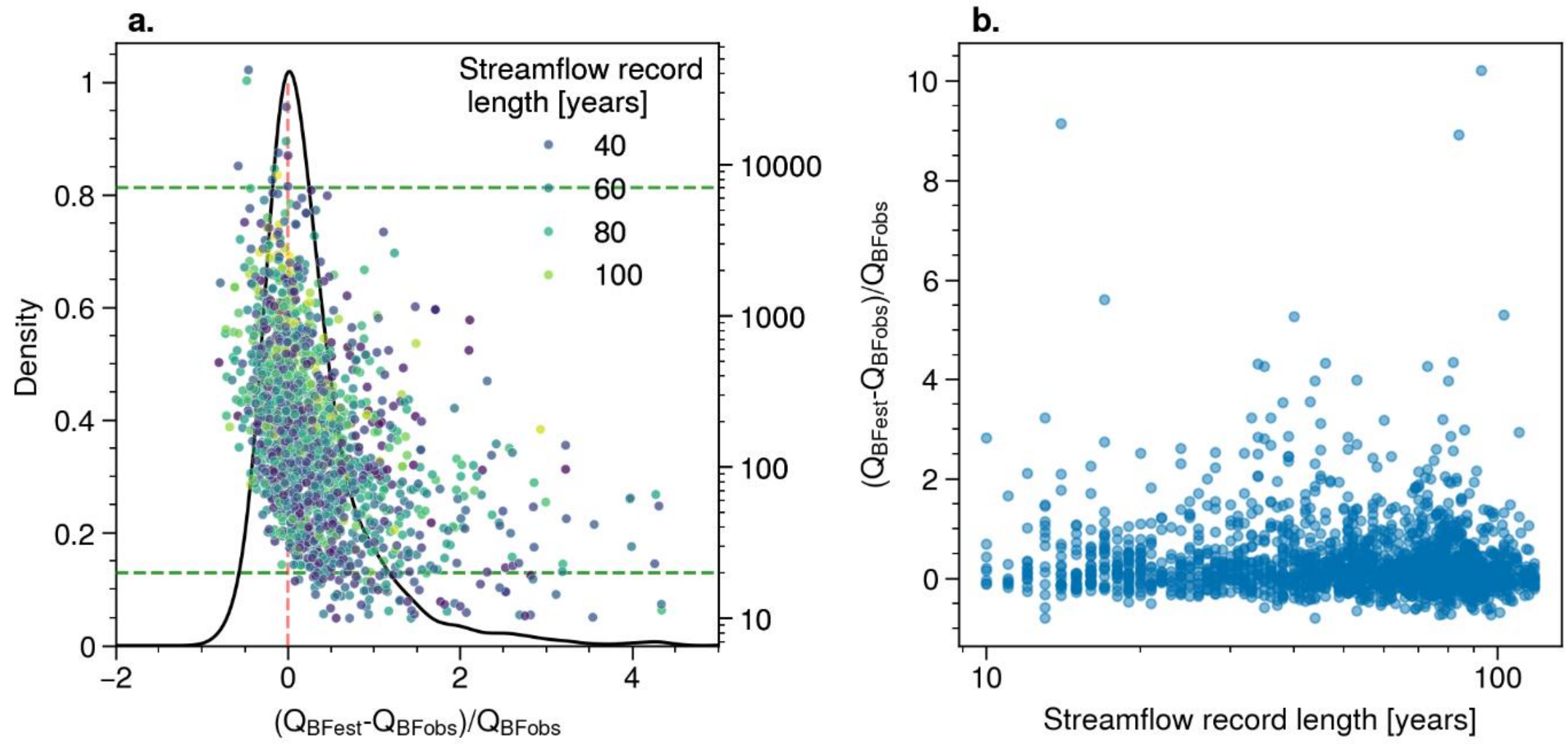

Fig. S9. Distribution of error in estimated bankfull discharge (Q_{BFest}) at 2,657 gauging stations. Black line is the error density distribution, with the x-axis representing the relative error in the Q_{BFest}, and the left y-axis showing the density value, coloured by the streamflow record length (panel a). Scatter points show the error distribution associated with the magnitude of Q_{BFest} (right y-axis, log scale). Q_{BFest} values smaller than 20 m^3s^{-1} (lower horizontal dashed green line) were all overestimated in the model (x>0), while Q_{BFest} values greater than 7,000 m^3s^{-1} (upper horizontal dashed line) were all underestimated (x<0). Panel b shows the streamflow record length (x-axis) versus the relative Q_{BFest} error (y-axis).

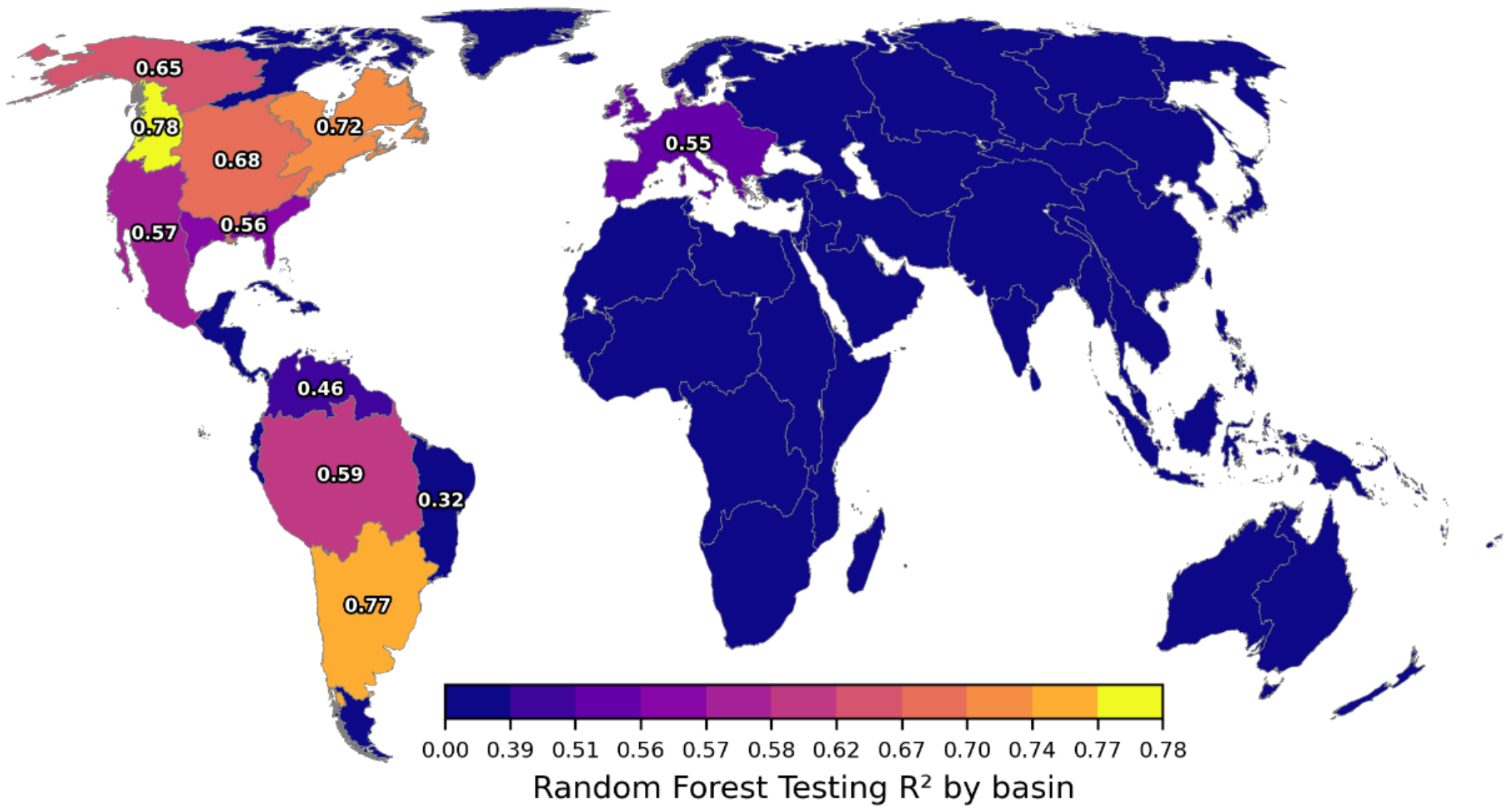

Fig. S10. Random Forest performance (R^2) for each basin, using each individual basin as the testing data and all the other basins as the training data. Basins with fewer than 10 values were excluded.

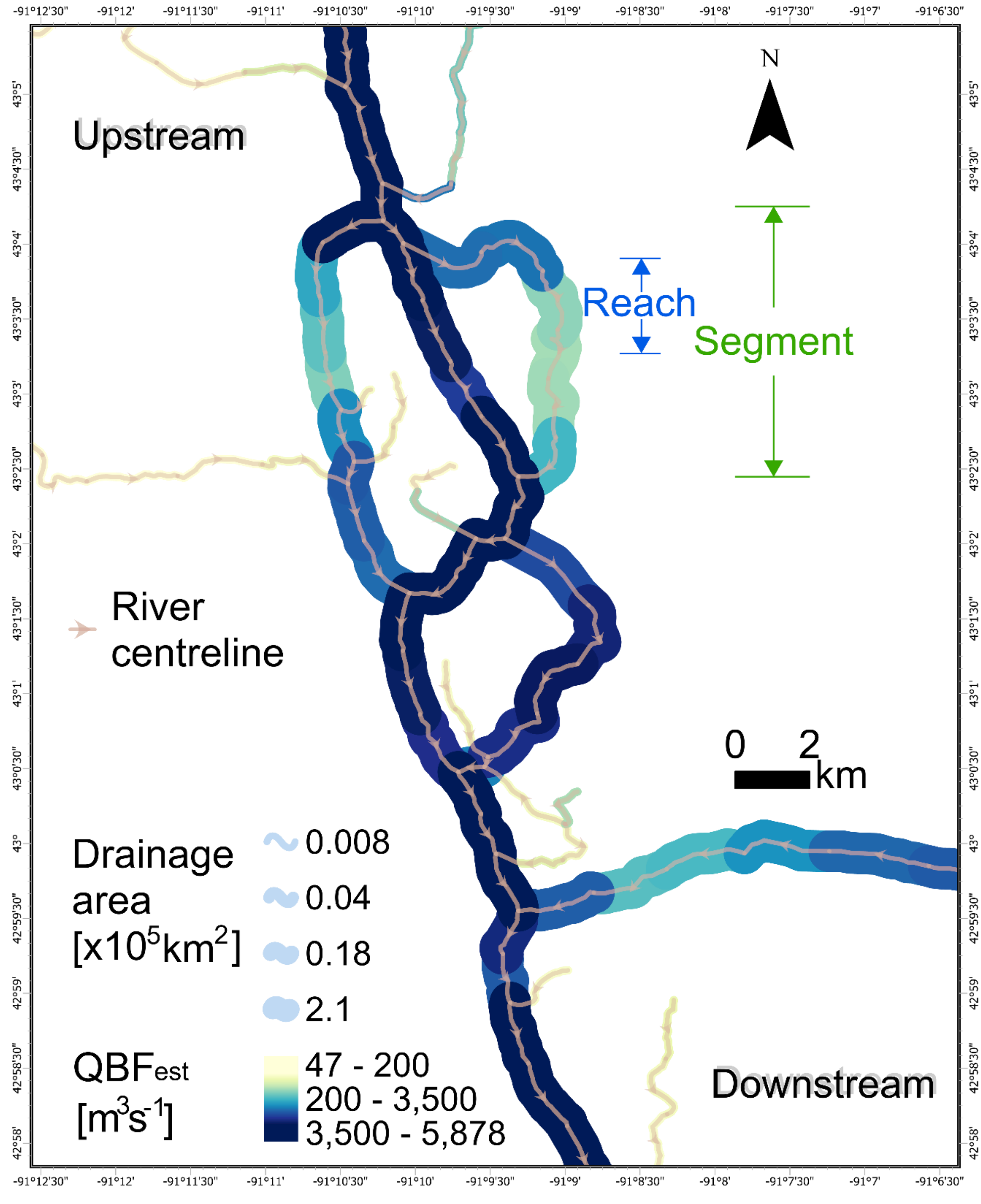

Fig. S11. An example of Q_{BFest} in the upper Mississippi River. The magnitude is coloured. The direction of river flow is indicated by an arrow at the centre of each river reach. Contributing drainage area is represented by line thickness at reach-scale. Reaches with width < 30 metres are not shown.

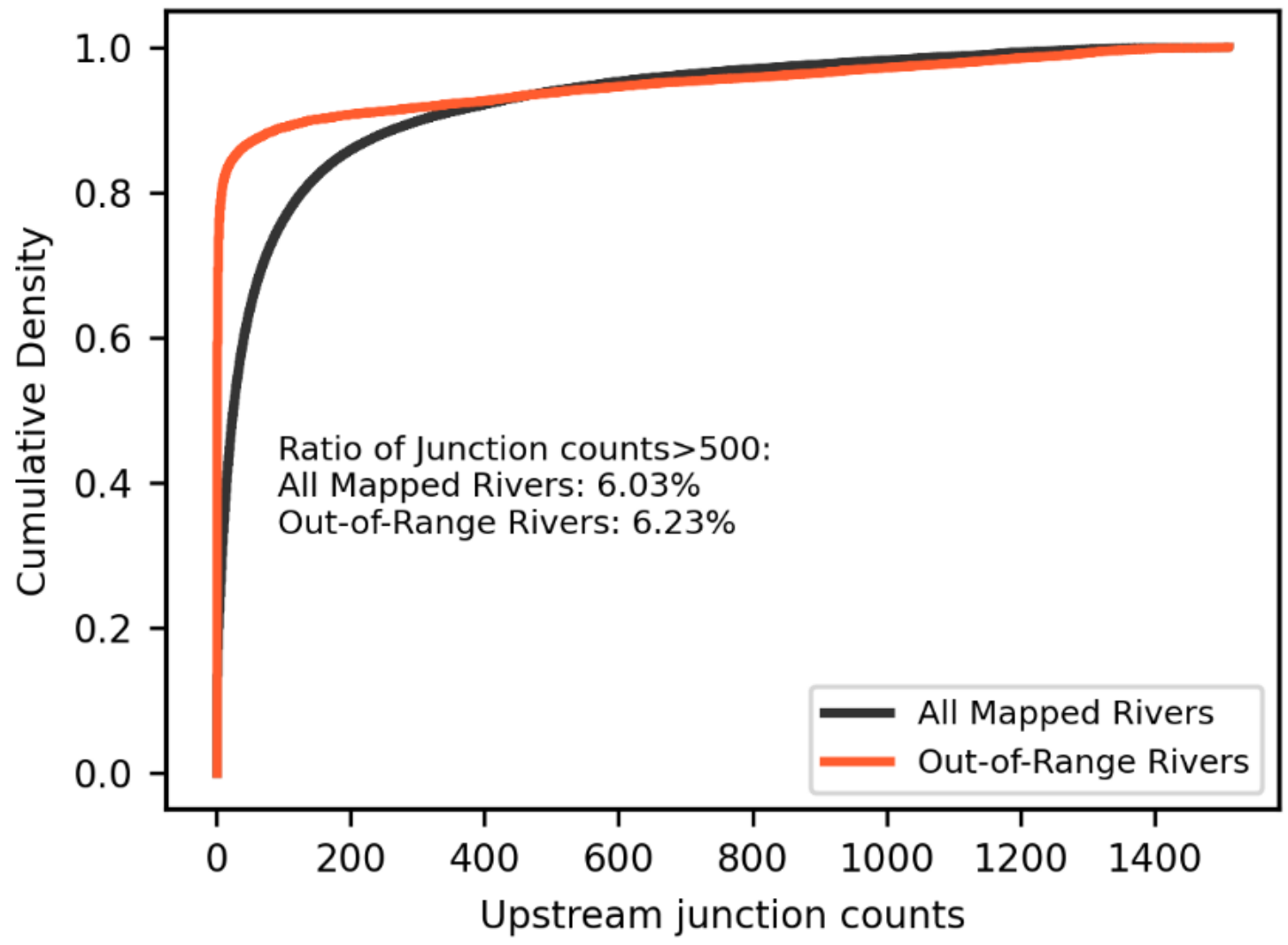

Fig. S12. Cumulative density distribution of upstream junction counts for all the rivers (~2.87 million km in length) and for the out-of-range rivers. Greater upstream junction counts indicate rivers located farther downstream within the river network and generally correspond to larger mainstem rivers.

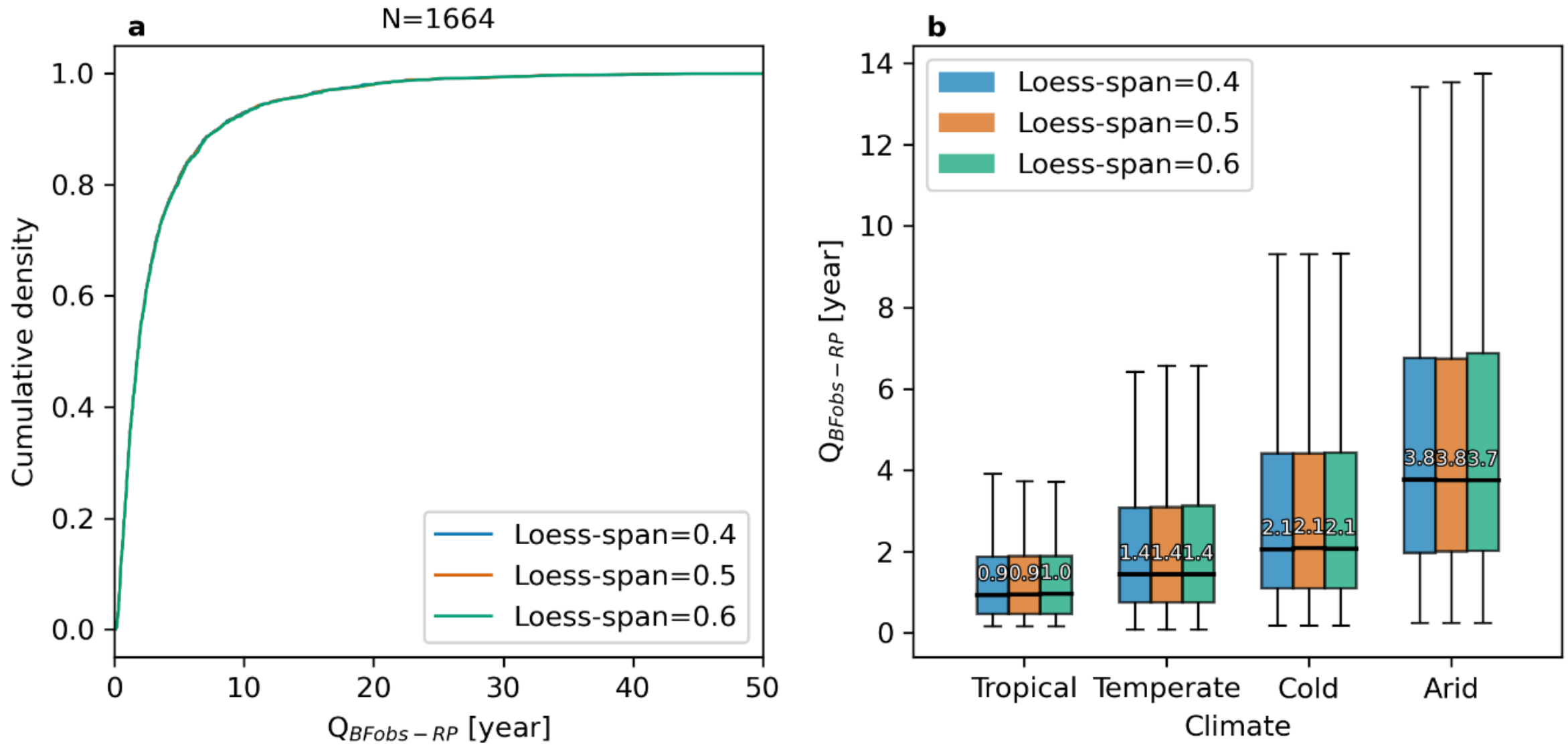

Fig. S13. Variation of the estimated return period for observed bankfull discharge with different span parameters of the Loess method at 1,664 stations. a. Cumulative density distribution coloured by the Loess span parameter. b. Boxplot of the estimated return period for each climate coloured by the Loess span parameter. A Loess span of 0.5 was used in this study.

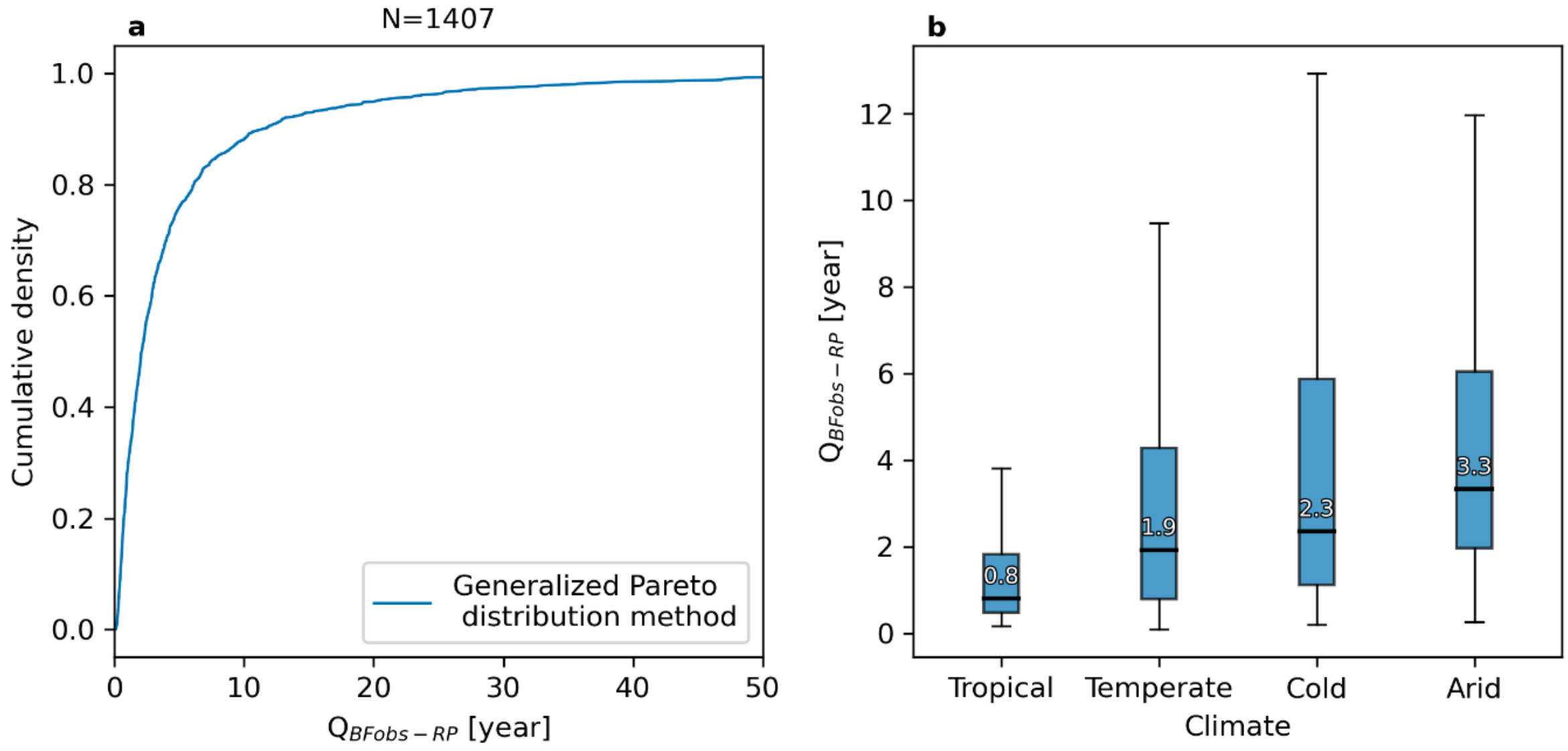

Fig. S14. The estimated return periods of observed bankfull discharge at 1,407 stations, using the Generalized Pareto distribution method. a. Cumulative density distribution. b. Boxplot of the estimated return period for each climate.

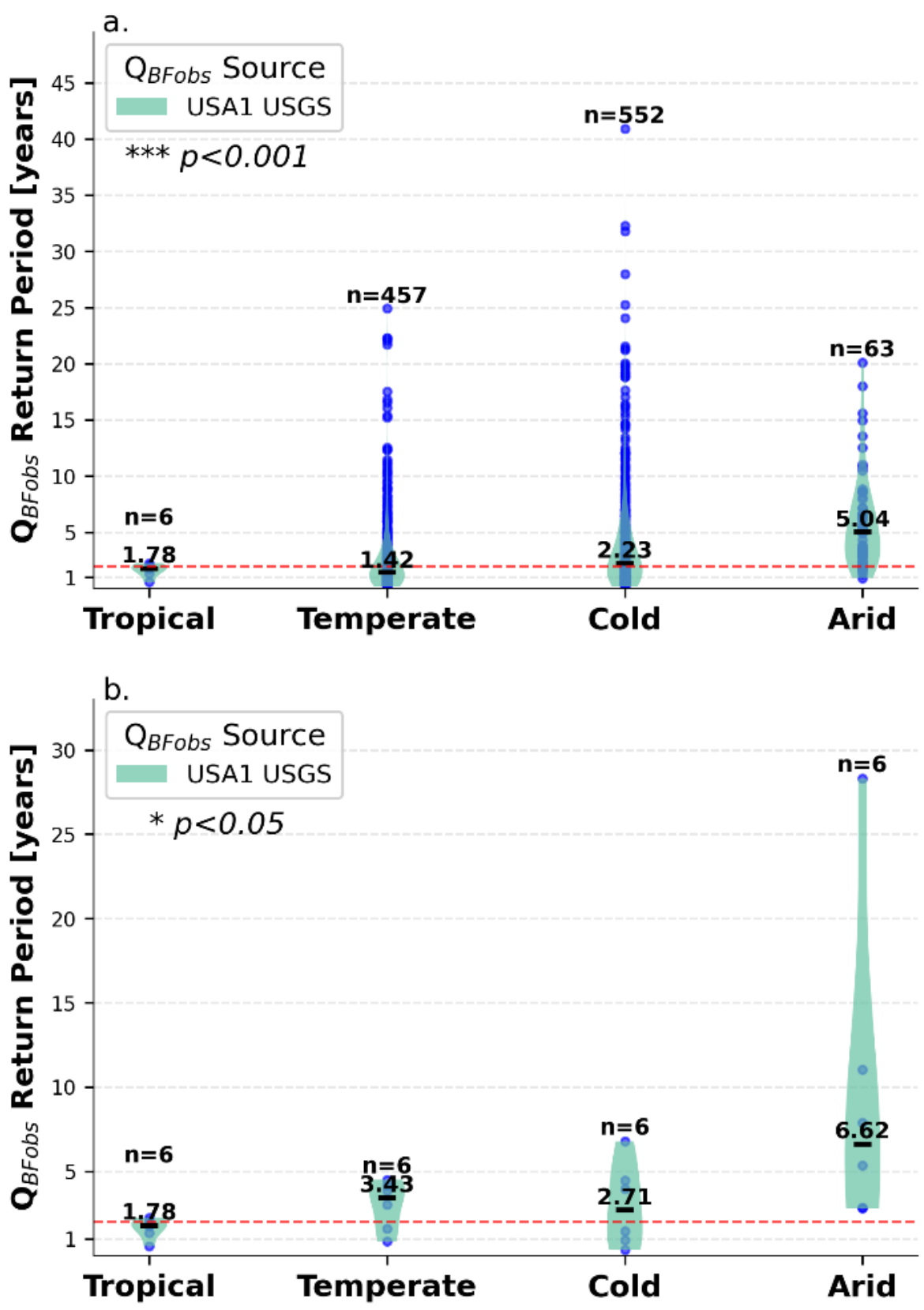

Fig. S15. Distribution of the return period of bankfull discharge across climate regions, derived from USA1 USGS (source names align with those in Fig. S1), a, all samples with calculated return period, b, six randomly sampled values within each climate (6 is the minimum sample size within all climates). The violin plots show the density distribution; blue scatters indicate the return period of each individual site; black lines mark the median return period for each climate, with the median value displayed. The red dashed line marks a return period equal to 2 years. The number of sites within each climate region is displayed at the top of the violin plots. The results of the significance test (Kruskal–Wallis) are annotated. Panel b is included only to illustrate the effect of equalising sample sizes across climate regions; results depend on the random sample selected and should not be interpreted as a robust inferential comparison.

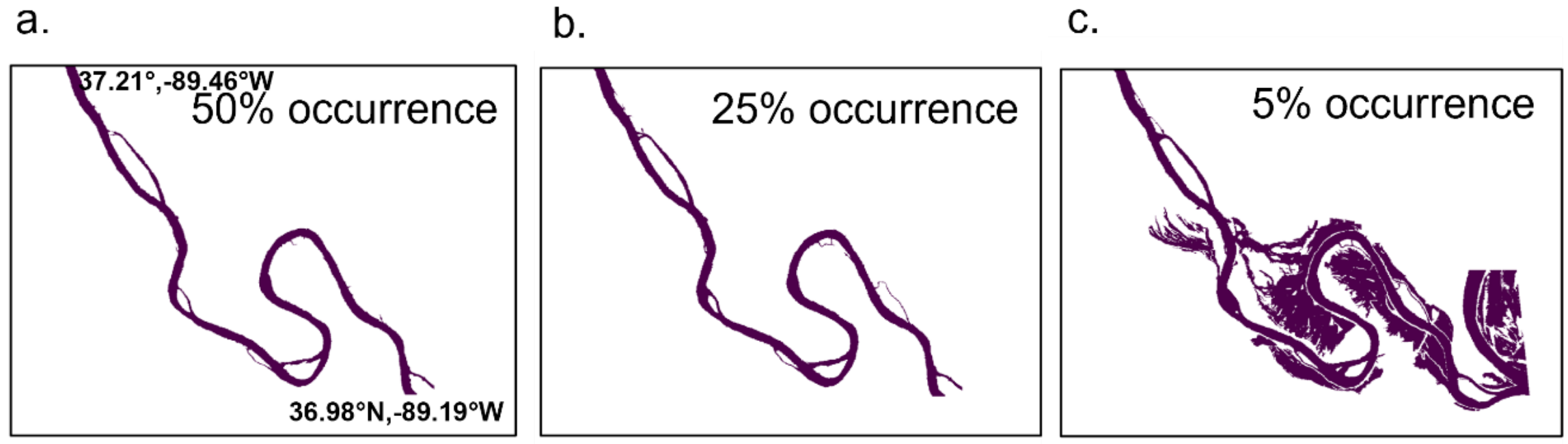

Fig. S16. Example of the water masks at three occurrence values from Landsat surface water occurrence data in the upper Mississippi River. The occurrence values are the ratio of the average monthly sum of water detections to the monthly sum of total valid observations over a 37-year period (1984-2021).

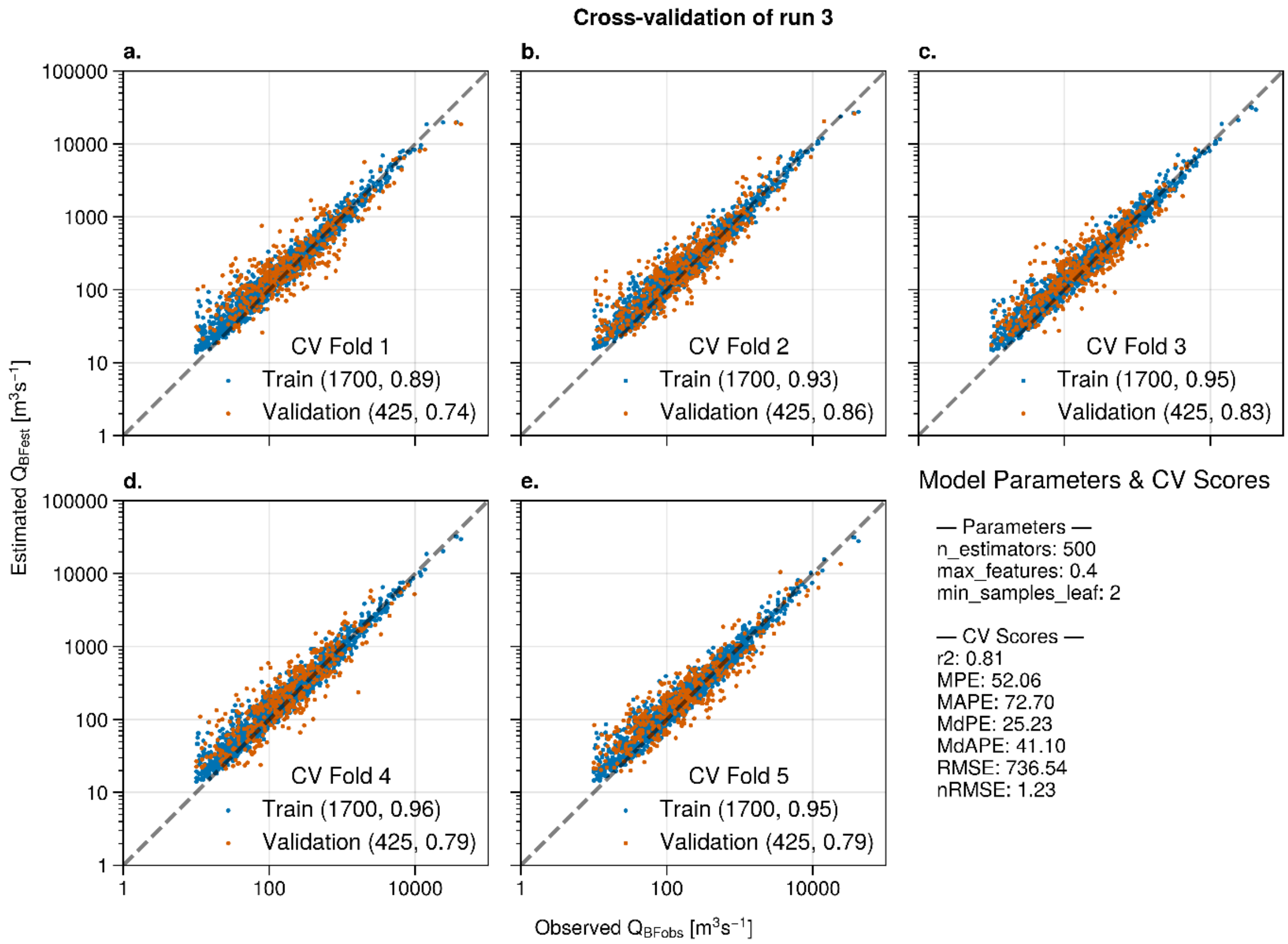

Fig. S17. Five-fold cross-validation results from one run (out of 10 model runs), shown with the tuned parameter set and associated error metrics.

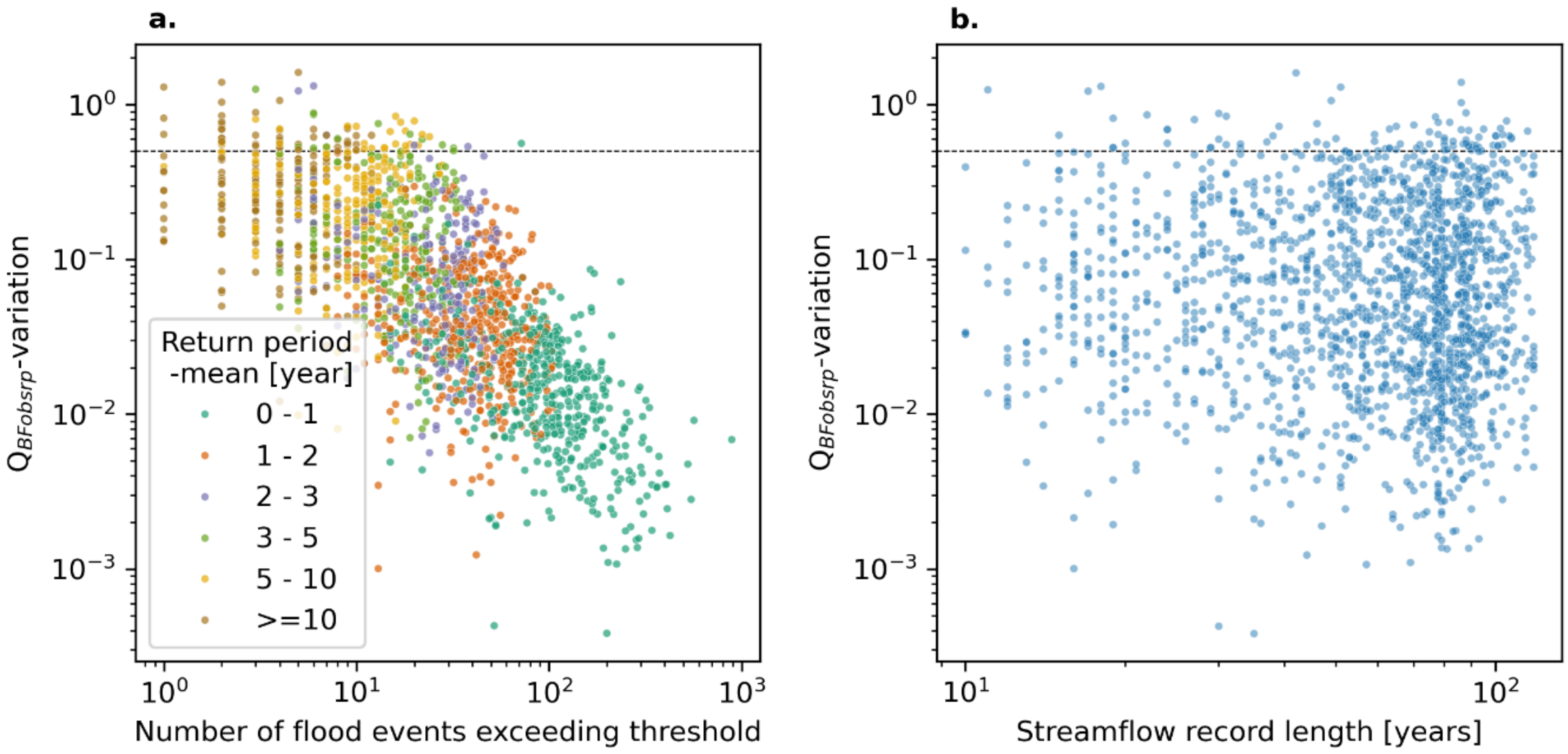

Fig. S18. Sensitivity of estimated bankfull discharge return periods to the choice of POT threshold using observed bankfull discharge (Q_{BFobs}). Relationship between the variation in the return period of the observed bankfull discharge ($Q_{BFobs\text{-}RP}$; y axis) and a. the number of events exceeding bankfull discharge (x-axis, log scale), and b. the length of streamflow records (x-axis, log scale). The variation is defined by the maximum $Q_{BF\text{-}RP}$ minus the minimum $Q_{BF\text{-}RP}$ divided by the mean $Q_{BF\text{-}RP}$. This variation is generated by applying 10%, 25%, 50%, 75% of the Q_{BFobs} as the threshold to selected flood events. The return period is estimated by extracting the flood probability from its relationship with the select flood events using the Loess function in R. These sites exclude those without high-quality daily discharge records (total complete years of streamflow record length >=10 years). Colour (legend) indicates the mean $Q_{BFobs\text{-}RP}$ at each site. Variability decreases as the number of events exceeding the threshold increases, but shows no clear relationship with streamflow record length. The dashed line marks the 50% variability (y=0.5) threshold used to exclude sites with return-period estimates that were highly sensitive to the POT threshold.

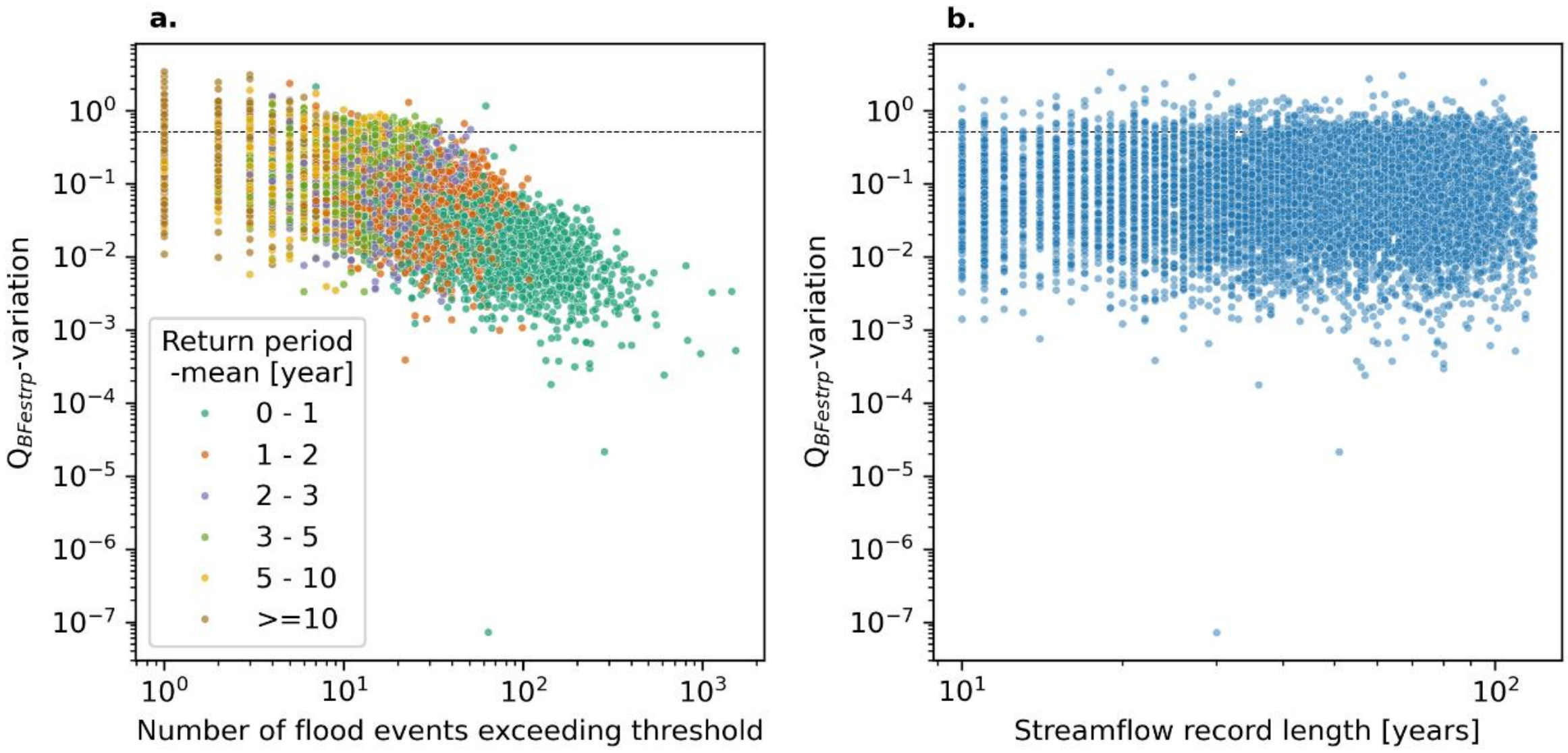

Fig. S19. Sensitivity of estimated bankfull discharge return periods to the choice of POT threshold using estimated bankfull discharge Q_{BFest}. Relationship between the variation in the return period of the estimated bankfull discharge: $Q_{BFest\text{-}RP}$ (y axis) and a. the number of events exceeding estimated bankfull discharge: Q_{BFest} (x-axis, log scale) and b. the length of streamflow records (x-axis, log scale). These sites exclude those without high-quality daily discharge records (total complete years of streamflow record length >=10 years). Variability decreases as the number of events exceeding the threshold increases, but shows no clear relationship with streamflow record length. The dashed line marks the 50% variability (y=0.5) threshold used to exclude sites with return-period estimates that were highly sensitive to the POT threshold.

Table S1. Predictors used for bankfull discharge estimation. CS: Catchment Sum (total value accumulated in the upstream catchment). CA: Catchment Average (mean of all raster values within the catchment), CM: Catchment Majority (the majority of all raster values within the catchment), RM: Reach Median, RA: Reach Average (median/mean, respectively, of all raster values within the river).

**Symbol**	**Data source and processing**	**Calculation**	**Unit**	**Reference**
DrainageA	FABDEM with a spatial resolution of ~30 m, computed based on topography, minimum 50 km^2.	CS	km^2	Wortmann et al.[31,32]
Discharge	GLOFAS v4.0 reanalysis daily discharge data from 1993 to 2022, gridded data at 0.05°. Average value over the time period was used.	CS	cum/s	Zoster et al.[60]
Runoff	GLOFAS v4.0 reanalysis annual total runoff data from 1993 to 2022, gridded data at 0.05°. Average value over the time period was used.	CS	cum/s	
GRWL, Overlap	Global River Width from Landsat (GRWL). Landsat-based river width at transects, minimum width 30 m. The overlap ratio between the GRWL river mask and the Global RIver Topology (GRIT).	RM	m, /	Allen & Pavelsky et al.[33], Wortmann et al.[31,32]
WRWs-1%, WRWs-10%, WRWs-20%, WRWs-30%, WRWs-40%, WRWs-50%	Wetted River Width at 1%, 10%, 20%, 30%, 40%, 50% Landsat-based water occurrence (occurrence range 0-100), width=wet areas/river reach length.	RA	m	Pekel et al.[61]
Sinuosity	River topology sinuosity, obtained from Global RIver Topology (GRIT) river attribute.	RA	/	Wortmann et al.[31,32]
GRanD	Global Reservoir and Dam database version 1. Vector data with location and capacity of reservoirs.	CS	m^3	Wang et al.[34]
Elevation	FABDEM Elevation with a spatial resolution ~30 m.	CA	m	Hawker et al.[53]
Prep	Mean annual precipitation from MSWX between 1979 and 2022, gridded data at 0.1°, average value over the above time period was used.	CA	mm	MSWX[64]

Symbol	Data source and processing	Calculation	Unit	Reference
Temp	Monthly average temperature from MSWX between 1979-2022, gridded data at 0.1°. Average value over time period was used.	CA	°C	MSWX[64]
Temp-range	Monthly minimal and maximum temperature range from MSWX between 1979-2022, gridded data at 0.1°. Monthly temperature range was calculated using maximum minus minimum temperature. Average value over months of the time period was used.	CA	°C	MSWX[64]
WTDH, WTDL	Water table depth-high and low. Gridded data at 0.25° annual data between 1970-2014.	CA	m	Guglielmo et al.[65]
SM_0-30, SM_30-60, SM_60-100	Soil Moisture at 0-30, 30-60, 60-100 cm Average value over time period was used.		m^3/m^3	
Soil-thick	Average soil and sedimentary deposit thickness (value range 0-50), gridded data at ~1 km.	CA	m	Pelletier et al.[66]
AI	Gridded Aridity Index data at ~1 km.	CA	/	Zomer et al.[67]
Koppen	The Koppen climate category.	CM	/	Beck et al.[38]
SnowC-5th, SnowC-50th, SnowC-95th	MODIS snow cover fraction, gridded data at ~1 km (value range 0-100), 5th, 50th, 95th percentile values of averaged annual maximum value over 2000-2019.	CA	/	Hengl et al.[68]
LAI	Gridded Leaf area index data at 0.05°, daily data between 1979 and 2022, average value is used.	CA	/	Vermote et al.[71]
FAPAR	Fraction of Absorbed Photosynthetically Active Radiation Daily mean 1979-2022.	CA	/	Vermote et al.[71]

Symbol	Data source and processing	Calculation	Unit	Reference
ImperviousP	Impervious percentage was calculated as urban areas divided by the pixel size 0.25° (value range 0-1), urban area is extracted from the land cover classification map at 2010.	CA	/	Copernicus Climate Change Service[72]

Table S2. Error metrics of training, cross-validation, and test data as well as that of the cross-validation and test data in each climate region of the random forest model (averaged across 10 runs). Metrics are defined in Text S2 below.

Type	Category	Count	R^2	MPE	MAPE	MdPE	MdAPE	RMSE	nRMSE
Overall	Train	2125	0.9	28	39	11	20	513	0.73
	Cross-validation	425	0.74	52	72	23	40	777	1.23
	Test	532	0.8	51	72	22	40	679	1.24
Cross-validation	Tropical	40	0.61	114	127	62	66	1664	2.07
	Arid	25	0.62	63	81	37	48	271	1.24
	Temperate	181	0.73	46	70	18	40	686	1.2
	Cold	180	0.78	42	61	21	37	353	0.95
Test	Tropical	50	0.77	116	130	57	60	1685	2.25
	Arid	31	0.64	61	78	41	51	285	1.13
	Temperate	226	0.77	45	70	17	39	442	1.14
	Cold	225	0.78	42	61	20	37	352	0.99
Test	Q>10000	2	/	-31	37	-31	38	10510	0.4
	Q5000-10000	4	/	-19	24	-19	22	2075	0.28
	Q1000-5000	44	/	-2	36	-10	31	942	0.47
	Q500-1000	68	/	2	38	-12	30	342	0.5
	Q100-500	216	/	28	52	11	34	185	0.78
	Q<=100	199	/	108	115	69	70	73	1.81

Text S2

Equations of the error metrics (R^2, MdPE, MPE, MAPE, MdAPE, RMSE, nRMSE) used to evaluate the Random Forest model are as follows:

$$R^2 = 1 - \frac{\sum_{i=1}^{i=n}(Q_{BFest} - Q_{BFobs})^2}{\sum_{i=1}^{i=n}(Q_{BFobs} - Q_{BFobs\_mean})^2} \quad (1)$$

$$MdPE = Median(\frac{Q_{BFest} - Q_{BFobs}}{Q_{BFobs}} \times 100\%) \quad (2)$$

$$MPE = (\frac{1}{n}) \sum_{i=1}^{i=n} \frac{Q_{BFest} - Q_{BFobs}}{Q_{BFobs}} \times 100\% \quad (3)$$

$$MAPE = (\frac{1}{n}) \sum_{i=1}^{i=n} \frac{|Q_{BFest} - Q_{BFobs}|}{Q_{BFobs}} \times 100\% \quad (4)$$

$$MdAPE = Median(\frac{|Q_{BFest} - Q_{BFobs}|}{Q_{BFobs}} \times 100\%) \quad (5)$$

$$RMSE = \sqrt{\frac{1}{n} \sum_{i=1}^{i=n} (Q_{BFest} - Q_{BFobs})^2} \quad (6)$$

$$nRMSE = \sqrt{\frac{1}{n} \sum_{i=1}^{i=n} \left(\frac{Q_{BFest} - Q_{BFobs}}{Q_{BFobs}}\right)^2} \quad (7)$$

where Q_{BFobs} is the observed bankfull discharge, Q_{BFest} is the estimated bankfull discharge, Q_{BFobs_mean} is the average value of the observed bankfull discharge, and *n* is the total number of values. The percentage error metrics (MdPE, MPE) indicate the degree to which the model overestimates (positive values) and underestimates (negative values) predictions. The absolute percentage error metrics (MAPE, MdAPE) and RMSE show the absolute error magnitude. The normalized RMSE (nRMSE) shows the relative error magnitude.
