# Supplementary material for: Global estimation of bankfull river discharge reveals distinct flood recurrences across climate zones
Source: Nat Commun. 2026 Aug 18;17:8179. doi: 10.1038/s41467-026-76433-3 (PMC13487230; doi:10.1038/s41467-026-76433-3)
Supplement: Supplementary file 2 [file 41467_2026_76433_MOESM2_ESM.pdf]

nature portfolio

Peer Review File

Global Estimation of Bankfull River Discharge Reveals Distinct Flood Recurrences Across Climate Zones

Corresponding Author: Dr Yinxue Liu

This file contains all reviewer reports in order by version, followed by all author rebuttals in order by version.

Attachments originally included by the reviewers as part of their assessment can be found at the end of this file.

Version 0:

Reviewer comments:

Reviewer #1

(Remarks to the Author)
Liu, Wortmann, et al. have used a machine learning approach to estimate bankfull discharge for 2.87 million km rivers globally. The compiled observed Qbf dataset is a very useful one, and the technicality is generally sound with lots of useful information presented for rivers across different climate regimes. While I think the paper has great potential to be a contribution to the community, several major concerns need to be carefully addressed.

Methods: Line 176 & Line 246: it would be vital to present what methods the authors used to calculate return periods from long-term daily discharge records? This needs to be explicitly referred to the FFA section in the end. Have the authors compared it against another FFA approach that would use at least 30-40 years of annual maximum data points? Since the fitted distribution matters a lot for the flood-frequency analysis, how does the selection of the loess span and fitting method introduce uncertainties for your results? This can largely influence the results presented in Figure 3 and Figure 4. So this needs to be carefully addressed with more text descriptions or SI figures.

Open data: in addition to estimated Qbf on GRIT, can the authors also provide the training Qbf data? Also, why is the raster data provided? How is that useful?

Out-of-distribution locations: although the authors have carefully discussed the patterns for the out-of-distribution river locations, one critical aspect is that these out-of-distribution reaches are all crucial locations that need Qbf information (i.e., major flooding locations requiring accurate Qbf information). Authors should carefully discuss how this might compromise the goals of global flood modeling and potential solutions to this.

Code: the provided code repository is a bit too simple (only two python scripts). Authors are suggested to provide a structure code directory, with a few working examples to walk through the entire workflow step by step. Some instructions and coding requirement (libraries) are also helpful information that's needed.

Line 91: literature 32 and 33 are indirect because they do not represent ML applications in characterizing the riverine systems. Authors are suggested to refer to Lin et al. (2020, GRL) and Messager et al. (2021, Nature), both are better sources to fit in the contexts.

Line 122-125: these arguments read a bit strange. Why the performance difference has something to do with the resolution? My understanding is that they both conceptualize channel capacity regardless of the spatial resolution. I have a hard time understanding this and please clarify.

Line 153: the definition of out-of-distribution and the motivation of why removing them needs to be presented before its first appearance.

(Remarks on code availability)
the provided code repository is too simple (only two python scripts). Authors are requested to provide a structure code directory, with a few working examples to walk through the entire workflow step by step. Some instructions and coding requirement (libraries) are also helpful information that's needed.

Reviewer #2

(Remarks to the Author)
This manuscript presents a first-of-its-kind analysis of bankfull discharge for the globe. This is a highly impactful, rigorous, and strong body of work that is appropriate for the journal. I have a few major comments that could require some heavier revision and then a larger number of minor comments to improve clarity and readability.

Major comments

1) Tone and context for results

From my understanding of the results, an important interpretation is that the historical assumption of a spatially-invariant 2-year bankfull recurrence interval is remarkably representative of rivers when taken on an average global basis. However, in some places in the manuscript, this important result is discounted or minimized. I would adjust the tone to reflect this point.

In general, places where "bias" is used to describe the spatially-invariant 2-year bankfull recurrence should be changed to "difference." To my mind, bias implies that there is a known truth to be measured against your estimates and the estimates of the spatially-invariant 2-year bankfull recurrence interval. However, I believe it is more accurate to say you are evaluating the differences that occur between the two methods.

Some other specific examples are:

- L49: Change "This new understanding…" to "This spatial refinement…"

- L71: Change to read "potentially introducing systematic differences in some regions in flood risk estimation."

- L120: Change to read: "...exhibits some over-estimation of bankfull width…"

- L123: Remove the word "slightly"

- L124-125: Delete the sentence starting with "This performance…" It is preferable, I think, to let the reader decide if the performance is acceptable.

- L126: Please add the units of RMSE to the values 1.32 and 1.66.

- L245: Change heading to "Differences in the commonly-use bankfull river discharge assumption" rather than biases. Your estimates could be biased as well and you don't have a truth to compare so you really are assessing differences. Same in Figure 4 captions - replace biases with differences. Replace within text elsewhere this occurs. You use differences in L285 so I don;t think it takes away from anything.

- L284: Change to "Our results assess"

- L307: Replace "improved" with "refined"

- L301 and L304: Change bias to differences

- L309" Remove the word "slightly." It is unclear how one could be so sure additional updated data would only "slightly" change estimates.

- In L386, the statement is made that this work "refutes" the prevailing assumption of the 2-year bankfull recurrence interval. However, as stated above, one could argue this work remarkably validates the bankfull recurrence of two years, a finding which is even more amazing given that the spatially-invariant value was determined without the level of detailed data used here. Even later in L399 the authors admit that "the 2-year assumption therefore provides a reasonable approximation…" and so it appears contradictory to the statement in L386.

- L394: Reword to state "highlight the systematic differences associated with the use of a spatially invariant bankfull return period in different regions."

- L403 makes the statement that the two-year assumption should be "obsolete" but I disagree. The 2-year assumption is shown to be remarkably good on an average basis. Furthermore, you have not tested smaller rivers and future work could further validate the 2-year assumption. I would delete this sentence.

2) Validation of the method

2a) In reading the main body of the text and the methods, I could not locate whether you repeatedly resampled 80% of the data to gain a more robust validation of the results rather than only one sampling of the dataset for validation. The Methods section notes a fivefold procedure but can you please clarify the number of times you resampled the dataset?

2b) Use of variability as a proxy for accuracy of out-of-sample estimates

- L159-160: The statement is made "We use the coefficient of variation (CV) of the RF's per-tree predictions as a simple proxy for relative uncertainty where observations are unavailable." I do not agree with the use of the CV of the estimates as a method to quantify uncertainty. Variability of the estimates alone - as measured here by the CV - is not a valid way to assess accuracy. For example, estimates can have a low variability but be completely wrong. I would remove statements referencing the use of variabikuty of the estimates to make any comment on the uncertainty of the estimated valuues. You have no observations for out-of-sample predictions with which to make any statement as to how the model performs for these cases.

- Again we see this logic in L222-225 that somehow variability of the results for out-of-sample estimates are somehow justification for the performance of out-of-sample estimates. Again, the consistency in estimates could be due to a systematic bias in your results rather than a commentary on accuracy. I would remove this statement. You have no way to assess the actual accuracy of out-of-sample estimates and any attempt to justify their validity through variability of the estimates is not appropriate. The work is already strong and these types of statements detract from the strength of your findings.

3) Limitations of the analysis

If I am reading correctly, it appears from L107 that the training dataset contains mainly large rivers that are 30 m or wider. As typical with machine-learning approaches, the results of the model should not be applied to locations that fall outside of the range of conditions under which the models were developed and the authors make note that out-of-sample locations were removed. However, it would be helpful to explicitly state the bounds of the training dataset. Different locations in the text (Figure 1, Methods section, as examples) explain that out-of-sample reaches account for 12.3% of the total reaches modeled (L154) but it is not clear what the thresholds were for drainage area and river width for a reach to quality as out-of-sample. Please note those values explicitly around L154, Figure 1 or the Methods section.

4) Improved clarity and communication

4a) Be consistent with your abbreviations. There are many cases where you have abbreviated a term but then still spell it out later. I have made note of these cases but it is possible that I missed some. Please carefully edit your manuscript with an eye towards consistency:

L112: "bankfull discharge observations" should be abbreviated to Qbfest.
L130: "estimated Qbf" should be changed to Qbf-est
L168: "estimated Qbf" should be changed to Qbf-est
L172: "Qbf estimates: should be Qbf-est
L191: "return period of the bankfull river discharge" to QBF-RP
L195: bankfull river discharge return periods to QBF-RPs
L221: observed and estimated bankfull discharge to QBF-obs and QBF-est
L223 and L224: use abbreviations for QBF-est

4b) Figure 1: I question the value of the maps in this figure. They are difficult to read and it is unclear what the reader is expected to observe from the maps. I also could not locate any grey lines showing the out-of-sample rivers.

4c) L165 states that "differences are statistically significant" but I could not locate what hypothesis test was used. The Dunn test and K-S test are mentioned later in the text but it is not clear if either of those tests were the one used here.

4d) The wording around bankfull recurrence interval could be improved. I offer some alternative wording to address this point:

- L39: Consider revising to read "...can transport before spilling over its banks, also known as the bankfall discharge…" This change can help distinguish large flows from overbank flow. Note that you also use a different explanation for bankfull discharge in L59 where you state bankfull discharge is the "maximum flow rate contained within a river just before inundation occurs on to the surrounding floodplain." It would improve clarity to be consistent in the manuscript as to your definition of bankfull discharge,

- L42: The current wording of "we deliver the first global estimation of bankfull discharge" reads as though you are providing one global value for all rivers. Consider revising to "we deliver the first update to global estimates.."

- In L176-181, it would be helpful to be more precise. For example, consider rewording to "...we calculated the return period for both QBF-obs and QBF-est distributed across the four climate regions. The return periods corresponding to bankfull river discharge (QBF-RP) vary widely across the globe. Overall, almost a quarter of sites are expected to exceed QBF several times a year on average…We find 15.6% of sites with bankfull discharges corresponding to a return period of 3.5 years…"

- L183: Change to read "..indicates that longer return periods (>3 years) associated with bankfull discharge predominantly

occur in arid regions and are less common in tropical climates.

- L186 is confusing. Bankfull discharge is one value and it is unclear what is intended by the statement “indicating more frequency bankfull status.” Consider revising to “Shorter return periods, indicating the probability of more frequent occurrence of discharges that exceed QBF…”

- L188-189 is also awkwardly worded. Consider “The median return period of the discharge associated with the observed (estimated) QBF-RP increases…”

- Consider changing “out-of-distribtuion” to “out-of-sample”. In my reading, distribution implies some type of probabilistic assessment.

4d) Editorial comments

L60: Change to read “This critical flow value…”

L67: Consider stating the number of rivers rather than saying “For most of the 37 rivers surveyed…”

L82: Consider revising to read: “Collecting such data is time-intensive and costly, and therefore, bankfull discharge…”

L88-90: Consider revising to read: “a host of novel global datasets have emerged that can be used to estimate bankfull discharge. These datasets include the location and connectivity of rivers, observation-based river widths, and dam and reservoir information.”

L92: Consider revising to read: “this rich variety of emerging datasets coupled with machine learning approaches…”

L93-96: Consider revising to read: “We present the first estimates of bankfull river discharge across the globe (GQBF), revealing distinct patterns of bankfull discharge return periods across climate regions, and compare the use of the 2-year return period bankfull discharge assumption relative to the GQBF.

L169: Reword to “higher for drainage areas smaller than 1000km2 and located in arid climates.

L316-317: Reword to state "... aligns with previous observations in localized areas that report highly variable..” Otherwise this sentence reads as though others have already done this analysis globally. I had to go to the citations to see that these cited studies were for local areas and not global analyses.

L341-344: Enumerate the 5 limitations that you explain further in the paragraph. I only see 3 listed in this introductory sentence but you actually number and explain 5 things in the paragraph.

L345-371: Excellent descriptions and analysis

L505: You are already abbreviate upstream accumulated (CS).

L528: Weird spacing in the text.

L531: You already abbreviate river catchment (CA).

L552: You already abbreviate river catchment (CA).

Figure 3a. Consider the text by the left arrow to read “bankfull discharge occurs less frequently than the mean annual flood” and the right arrow “Bankfull discharge occurs more frequently than the mean annual flood”

(Remarks on code availability)

Reviewer #3

(Remarks to the Author)
Please find the attached document for my full review.

(Remarks on code availability)

Version 1:

Reviewer comments:

Reviewer #1

(Remarks to the Author)
I think the authors have carefully addressed all my comments and the dataset is a good contribution to the community. I have no further comments on the main texts except for some minor issues regarding code availability.

(Remarks on code availability)
The authors have added more details regarding the code, but the results are still not fully reproducible because the training data cannot be released. In this case, can the authors consider giving some working examples? Currently what's in the /example directory is not workable examples. This reduces the reproducibility. More strategies to improve reproducibility is highly recommended.

Reviewer #2

(Remarks to the Author)
I have now read the responses to the review comments and the revised manuscript. The authors did an exceptional job in thoughtfully revising the manuscript according to the reviewer comments. Notable improvements include a complete revision of the figures in the manuscript, expansion and clarification of the methods, and a detailed sensitivity analysis of the methodological choices used to estimate the return periods.

While there are limited instances where the authors disagreed with my comments, I fully accept their rebuttal and reasoning. Even in those instances, the authors thought carefully about the spirit of the comment and further strengthened the manuscript.

I have no further comments and recommend the manuscript be accepted. It was a delight to be part of the colleague review process and I look forward to seeing the manuscript published.

(Remarks on code availability)

Open Access This Peer Review File is licensed under a Creative Commons Attribution 4.0 International License, which permits use, sharing, adaptation, distribution and reproduction in any medium or format, as long as you give appropriate credit to the original author(s) and the source, provide a link to the Creative Commons license, and indicate if changes were made.
In cases where reviewers are anonymous, credit should be given to 'Anonymous Referee' and the source.
The images or other third party material in this Peer Review File are included in the article's Creative Commons license, unless indicated otherwise in a credit line to the material. If material is not included in the article's Creative Commons license and your intended use is not permitted by statutory regulation or exceeds the permitted use, you will need to obtain permission directly from the copyright holder.
To view a copy of this license, visit https://creativecommons.org/licenses/by/4.0/

REVIEWER COMMENTS

Reviewer #1 (Remarks to the Author):

Liu, Wortmann, et al. have used a machine learning approach to estimate bankfull discharge for 2.87 million km rivers globally. The compiled observed Qbf dataset is a very useful one, and the technicality is generally sound with lots of useful information presented for rivers across different climate regimes. While I think the paper has great potential to be a contribution to the community, several major concerns need to be carefully addressed.

We thank the reviewer for the positive evaluation of our manuscript, and have addressed the major concerns as described below. All line numbers in this document refer to the clean revised manuscript.

Methods: Line 176 & Line 246: it would be vital to present what methods the authors used to calculate return periods from long-term daily discharge records? This needs to be explicitly referred to the FFA section in the end. Have the authors compared it against another FFA approach that would use at least 30-40 years of annual maximum data points? Since the fitted distribution matters a lot for the flood-frequency analysis, how does the selection of the loess span and fitting method introduce uncertainties for your results? This can largely influence the results presented in Figure 3 and Figure 4. So this needs to be carefully addressed with more text descriptions or SI figures.

We agree. In the revised manuscript, we have clarified the FFA method in the Results section and provided explicit references to the implementation in the Methods and Code Availability sections. We adopted the peak-over-threshold method rather than the annual maximum approach because the latter cannot resolve return periods shorter than one year (Lines 567-570). Regarding the record length, we show that streamflow record length has no apparent impact on the estimated return period (Supplementary Figs. S17-18 panel b).

Regarding the methodology, we conducted additional tests to assess the sensitivity of the estimated return period to 1) the Loess span parameter, 2) fitting method. We tested different Loess span parameters (0.4, 0.5, 0.6) and find the choice of span has a negligible impact on the estimated return periods (Supplementary Fig. S13, also included below). We also tested another approach, using the Generalized Pareto distribution, i.e., one of the most commonly-used fitting methods for flood frequency analysis (FFA), suitable for Peak-over-threshold selected flood peaks. The estimated return period results are very similar to our current method (Supplementary Fig. S14, also included below), showing that the impact of fitting method on the estimated return period is also marginal. We also included an example of the estimated return period using the Generalized Pareto distribution with varied flood peak thresholds below (Fig. R1).

The marginal impact of the Loess span and fitting method on the estimated return period (see Fig. S13) is expected because, for discharges near bankfull conditions, most flood peaks are closely spaced in magnitude (i.e., incremental increases rather than order-of-magnitude jumps), such that different fitting approaches have minimal influence on the estimated return period (Schneider et al., 2011). Previous studies have shown that methodological differences in FFA primarily affect estimates of extreme events (e.g., 100-year floods) rather than more frequent events (Zhou et al., 2022).

Overall, our use of a consistent FFA framework for estimating return periods of observed and modelled bankfull discharge, as well as the 2-year flood, ensures that our conclusions regarding climate-dependent variation in bankfull discharge return periods, and the bias in the 2-year assumption, are robust. We have added a statement discussing the Loess span parameter setting and fitting method (Lines 594–595).

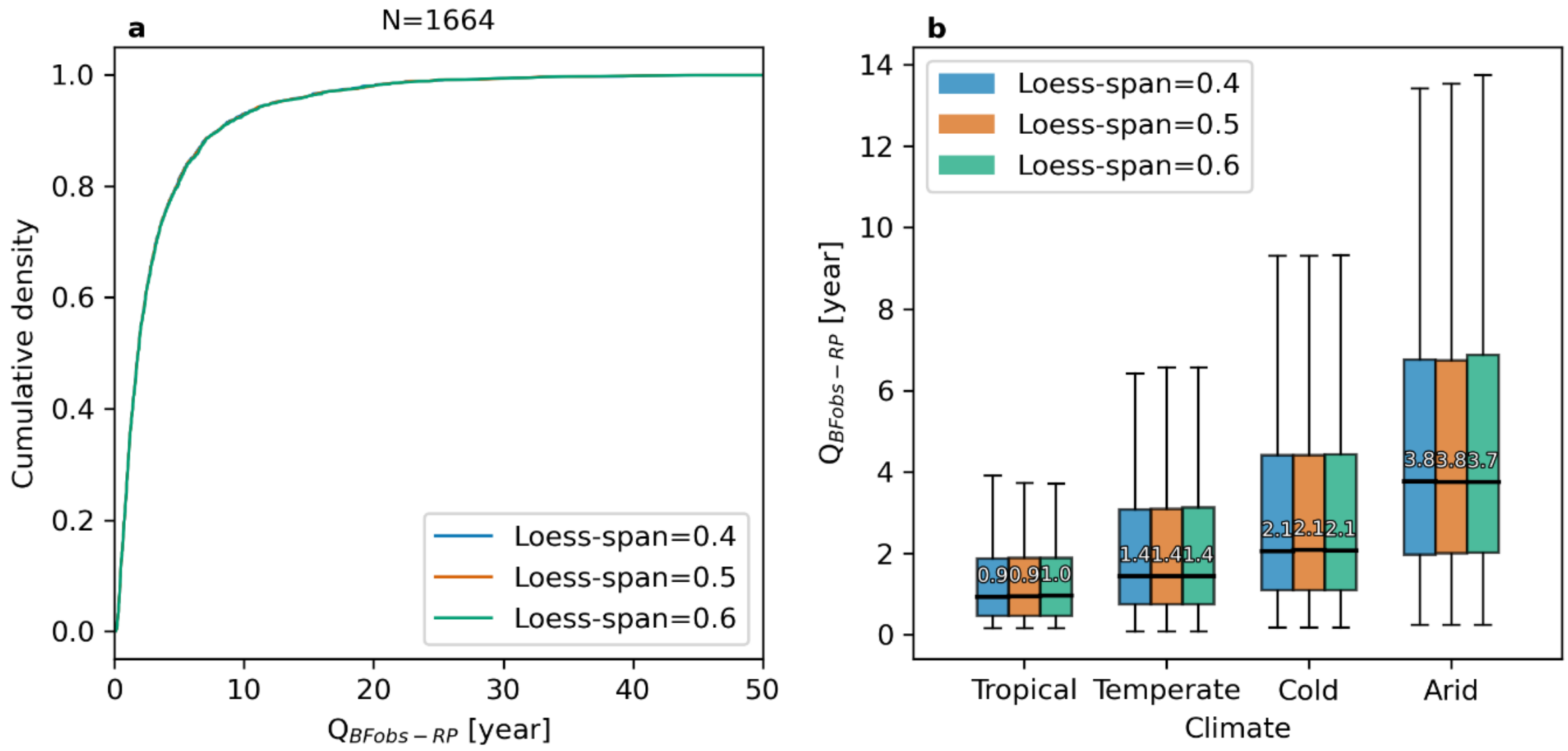

Fig. S13. Absence of variation in estimated return period for observed bankfull discharge using different Loess span parameters at 1,664 stations. a. Cumulative density distribution coloured by the Loess span parameter. b. Boxplot of the estimated return period for each climate coloured by the Loess span parameter. A Loess span of 0.5 was adopted in the manuscript.

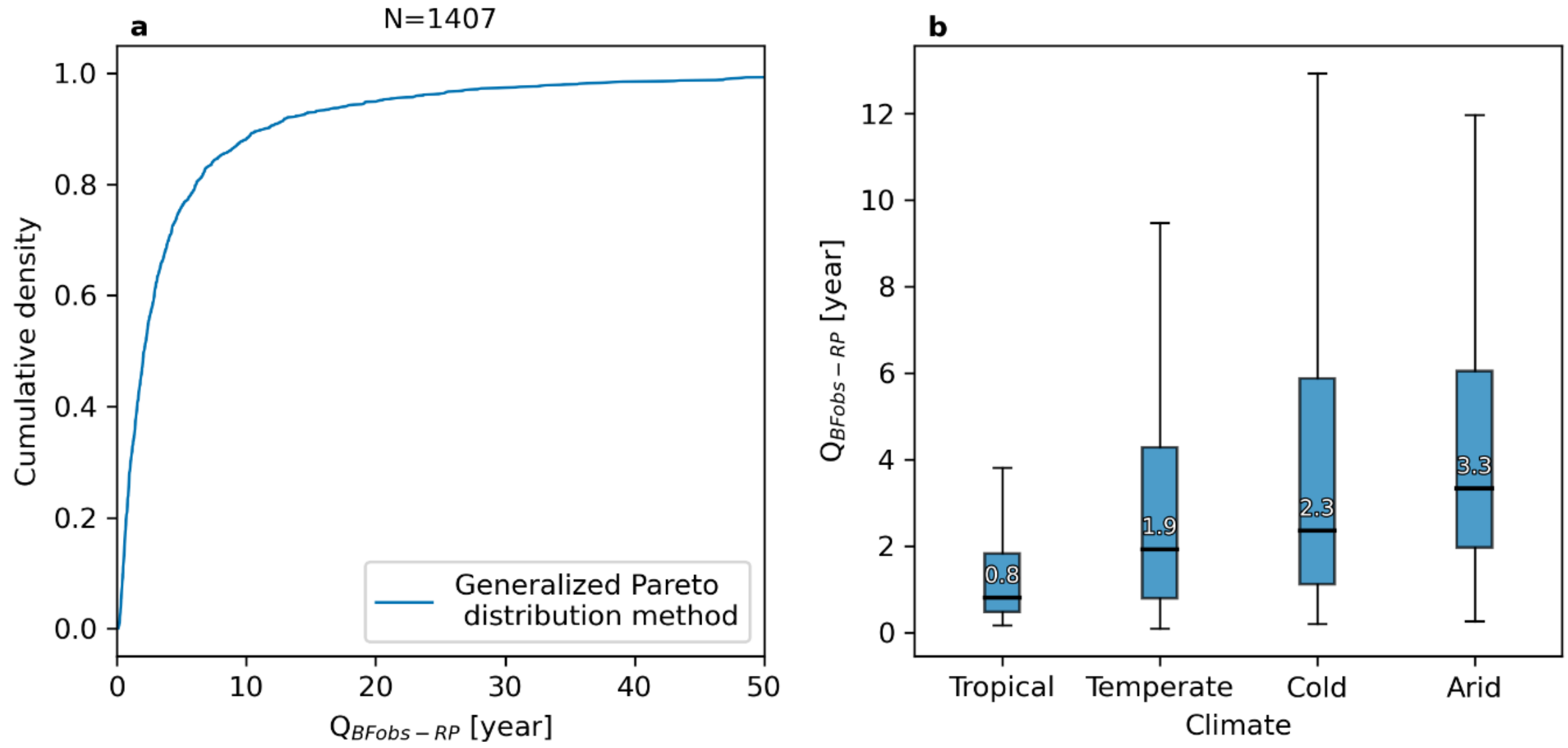

Fig. S14. Return period of observed bankfull discharge at 1,407 stations, estimated using the Generalized Pareto distribution approach. a. Cumulative density distribution. b. Boxplots for each climate coloured by the Loess span parameter.

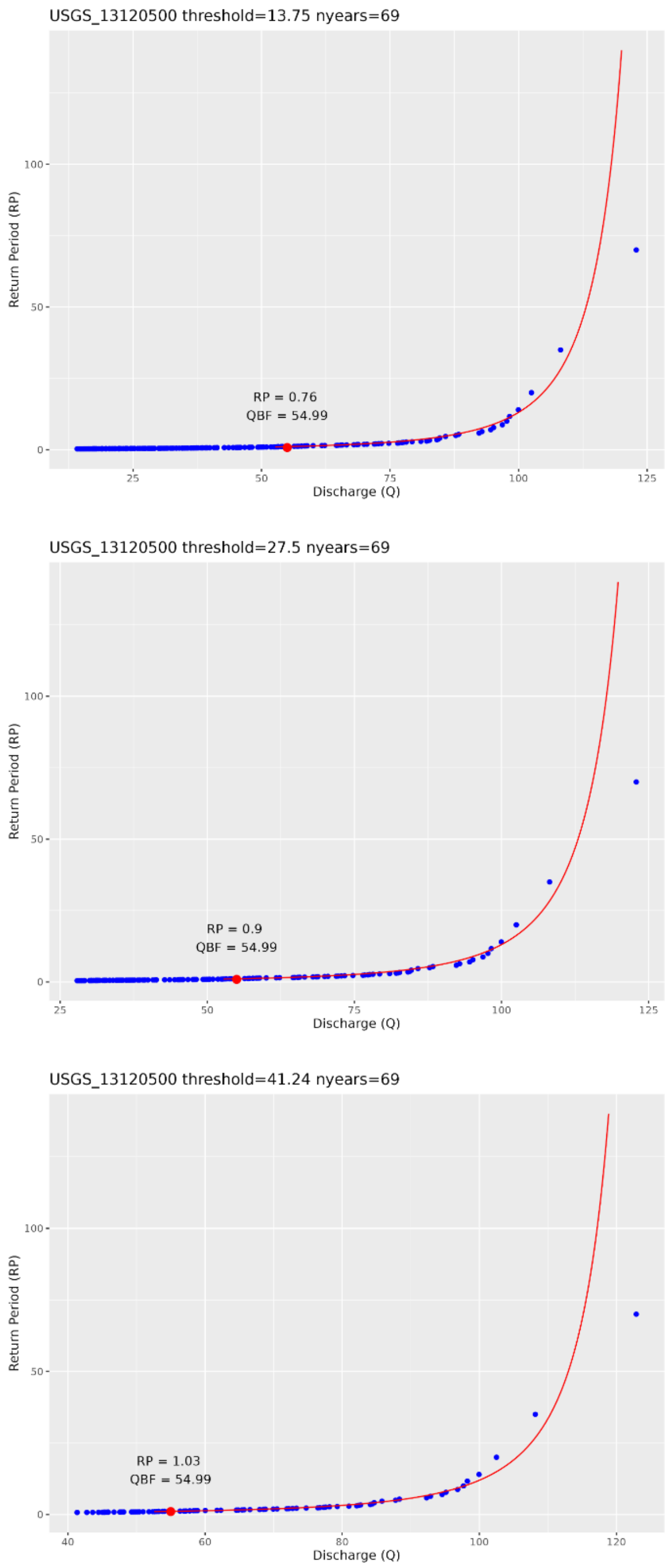

Fig R1. An example of using the fitted Generalized Pareto distribution (GPD) to estimate the bankfull discharge return period obtained using significantly varying flood peak thresholds (discharge magnitude in m^3s^{-1}) at station no. USGS13120500. Blue dots indicate the selected flood peaks with the threshold labelled above each panel. Red line is the fitted curve from the GPD method. Red dot indicates the observed bankfull discharge and the estimated return period. The total number of valid years of streamflow data is labelled above each panel (69 years).

Reference:

Schneider, C., Flörke, M., Eisner, S., & Voss, F. (2011). Large scale modelling of bankfull flow: An example for Europe. *Journal of Hydrology*, *408*(3-4), 235-245.

Zhou, X., Ma, W., Echizenya, W., & Yamazaki, D. (2021). The uncertainty of flood frequency analyses in hydrodynamic model simulations. Natural Hazards and Earth System Sciences, 21(3), 1071-1085. https://nhess.copernicus.org/articles/21/1071/2021/

Open data: in addition to estimated Qbf on GRIT, can the authors also provide the training Qbf data? Also, why is the raster data provided? How is that useful?

Raster data: While we encourage the community to adopt more realistic river network representations such as GRIT, we also recognize that many users rely on alternative river network datasets and may wish to apply our bankfull discharge estimates within those frameworks. To support this, we provide rasterized data that allow users to extract bankfull discharge values at the grid-cell level based on location, independent of any specific river network representation.

Training data: Although we would prefer to make the observed bankfull discharge dataset fully publicly available, it was compiled from multiple sources across different institutions, and we do not have permission to redistribute all of the underlying data. However, we provide detailed information on the original data sources in Supplementary Text S1, including guidance on how to access these datasets where they are publicly available.

Out-of-distribution locations: although the authors have carefully discussed the patterns for the out-of-distribution river locations, one critical aspect is that these out-of-distribution reaches are all crucial locations that need Qbf information (i.e., major flooding locations requiring accurate Qbf information). Authors should carefully discuss how this might compromise the goals of global flood modeling and potential solutions to this.
We appreciate this comment from the reviewer and agree that these out-of-distribution locations (shown in grey in the manuscript figure) are critical for global flood modelling. Accordingly, in the revised manuscript, we have added a discussion of the limitations associated with out-of-distribution locations, as well as potential approaches to address them (Lines 307–316). At present, there is no optimal solution, as Qbf cannot be reliably estimated in the largest rivers where observations are not available. In the future, as more observations become available, we hope to provide updated versions of the dataset. In the meantime, possible interim approaches include using climate-adjusted flood return periods as proxies. In addition, river widths in these regions are often well constrained from satellite observations; therefore, bankfull hydraulic geometry could potentially be inferred by combining width with assumptions for the associated parameters. The major uncertainty arises from the determination of bankfull river width and assumptions of hydraulic geometry

parameters. Satellite-derived river widths may be underestimated relative to bankfull widths when the imagery is acquired at flows below bankfull stage.

In the longer term, increased observational coverage may help address this gap. For example, the accumulation of time series of water surface elevation data, width and bank shapes from SWOT over time may provide useful constraints on bankfull conditions, particularly for the main stems of large rivers (Cerbelaud et al., 2026).

Reference:

Cerbelaud, A., Wade, J., David, C.H. et al. Wide-swath altimetry maps bank shapes and storage changes in global rivers. Nature 651, 666–671 (2026). https://doi.org/10.1038/s41586-026-10218-y

Code: the provided code repository is a bit too simple (only two python scripts). Authors are suggested to provide a structure code directory, with a few working examples to walk through the entire workflow step by step. Some instructions and coding requirement (libraries) are also helpful information that's needed.

We thank the reviewer for this helpful suggestion. In the revised manuscript, we have substantially improved the code documentation. Specifically, we now provide a *README.txt* file with step-by-step instructions, the *environment.yml* files to facilitate reproducible environment setup, and detailed workflows covering all major analyses.

These include: (i) construction of the Random Forest model, (ii) cross-validation and error metric calculations for training, testing, and cross-validation datasets (for all samples and across climate categories), (iii) permutation importance analysis, (iv) return period estimation of bankfull discharge and estimating 2-year return period of flood magnitude.

The code and associated instructions can be accessed here: https://github.com/YinxueLiu/G-QBF.

Line 91: literature 32 and 33 are indirect because they do not represent ML applications in characterizing the riverine systems. Authors are suggested to refer to Lin et al. (2020, GRL) and Messager et al. (2021, Nature), both are better sources to fit in the contexts.
We thank the reviewer for this suggestion. In the revisions, we followed this advice and changed the citations.

Line 122-125: these arguments read a bit strange. Why the performance difference has something to do with the resolution? My understanding is that they both conceptualize channel capacity regardless of the spatial resolution. I have a hard time understanding this and please clarify.
We thank the reviewer for this comment. We agree that the conceptualization of channel capacity itself is not dependent on spatial resolution. Our intention was instead to highlight

that model performance may vary across resolutions because the statistical properties of the observed data change with aggregation scale.

At finer spatial resolutions, the data exhibit greater local heterogeneity and more extreme values, which are inherently more difficult for the model to capture. In contrast, aggregation to coarser resolutions smooths local variability and reduces the influence of extremes, often resulting in lower prediction errors overall.

For models such as random forests, prediction errors are typically larger at the tails of the distribution. Because aggregation reduces the prominence of these extremes, coarser-resolution products may achieve lower MPE values. We have clarified this explanation in the revised manuscript.

In the revisions, we rewrote the sentence as below to be explicit.

‘*The Mean Percentage Errors (MPEs) are 28% for training and 44% for testing, which are greater than those reported in a previous study conducted in Europe at a coarser grid resolution of 5 arc min[26] (22.8%). This difference likely reflects the greater local variability present at finer spatial resolutions, which is more difficult for the model to represent accurately. In addition, MPE is particularly sensitive to extreme values, which are more prevalent at finer resolutions. Aggregation to coarser resolutions may smooth these extremes and reduce variability, thereby contributing to lower overall errors.*’

Line 153: the definition of out-of-distribution and the motivation of why removing them needs to be presented before its first appearance.
We thank the reviewer for these comments. In the revisions, we have clarified these aspects (Line 160-164).

Reviewer #1 (Remarks on code availability):

the provided code repository is too simple (only two python scripts). Authors are requested to provide a structure code directory, with a few working examples to walk through the entire workflow step by step. Some instructions and coding requirement (libraries) are also helpful information that's needed.

In the revisions, we have substantially improved the readability and accessibility of the code. Please read details in the response to the code comment above.

Reviewer #2 (Remarks to the Author):

This manuscript presents a first-of-its-kind analysis of bankfull discharge for the globe. This is a highly impactful, rigorous, and strong body of work that is appropriate for the journal. I have a few major comments that could require some heavier revision and then a larger number of minor comments to improve clarity and readability.

We thank the reviewer for the positive evaluation of our manuscript and for recognising the novelty and potential impact of this work. We appreciate the constructive feedback and the identification of both major and minor points, which have helped us improve the clarity, robustness, and presentation of the study. We have carefully addressed each comment in detail below. All line numbers in this document refer to the clean revised manuscript.

Major comments

1) Tone and context for results

From my understanding of the results, an important interpretation is that the historical assumption of a spatially-invariant 2-year bankfull recurrence interval is remarkably representative of rivers when taken on an average global basis. However, in some places in the manuscript, this important result is discounted or minimized. I would adjust the tone to reflect this point.
In general, places where "bias" is used to describe the spatially-invariant 2-year bankfull recurrence should be changed to "difference." To my mind, bias implies that there is a known truth to be measured against your estimates and the estimates of the spatially-invariant 2-year bankfull recurrence interval. However, I believe it is more accurate to say you are evaluating the differences that occur between the two methods.

On the first point, we agree with the reviewer that our analysis shows the 2-year return period assumption is, on average, representative of bankfull discharge across thousands of observed sites globally. Our expanded dataset does not overturn the findings based on the 37 rivers investigated by Wolman & Leopold. However, our work highlights a new result: the climate dependence of the return period associated with bankfull discharge. The agreement on a log–log scale arises largely because the magnitude differences between bankfull discharge and the 2-year flood are relatively small in general. In typical flood frequency curves, the flood magnitude differences between frequent events (e.g., 1-year vs. 3-year return periods) are modest, which can mask systematic variation in the return period. However, accounting these variations is important, particularly for large-scale flood inundation modelling and flood risk estimates. Because even small differences in bankfull discharge can substantially influence flood inundation modelling (propagating through flood wave dynamics and degrading model accuracy), particularly for smaller, high-frequency

events that are critical for quantitative risk assessment (Neal et al., 2021; De Paiva et al., 2013). Therefore, adopting climate-relevant return periods instead of a fixed 2-year assumption is likely to substantially affect global flood risk assessments, despite its apparent agreement on average. We have clarified these points in the revised manuscript (Line 75-78). We have also reframed the text to emphasize the implications of climate-dependent variation in bankfull discharge return periods throughout.

On the second point, we clarify that we quantify deviations of the 2-year return period of flood magnitudes relative to the *observed* bankfull discharge measurements (or channel capacity). Therefore, here we used the phrase "bias" referring to assumption against observation. This "bias" is in a statistical sense (difference relative to observation-based reference) rather than implying that the observations represent an error-free ground truth. We therefore retain the term "bias" to describe the deviation between the 2-year return period discharge and the discharge associated with the observed bankfull return period. We have clarified this in the text at lines 98-100.

References:

Neal, J., Hawker, L., Savage, J., Durand, M., Bates, P., & Sampson, C. (2021). Estimating river channel bathymetry in large scale flood inundation models. *Water Resources Research*, *57*(5), e2020WR028301. https://doi.org/10.1029/2020WR028301

De Paiva, R. C. D., Buarque, D. C., Collischonn, W., Bonnet, M. P., Frappart, F., Calmant, S., & Bulhões Mendes, C. A. (2013). Large-scale hydrologic and hydrodynamic modeling of the Amazon River basin. *Water Resources Research*, 49(3), 1226-1243. https://doi.org/10.1002/wrcr.20067

Some other specific examples are:
- L49: Change "This new understanding…" to "This spatial refinement…"

We understand the reviewer's concern. However, we argue that our work does not constitute a spatial refinement; rather, it provides a conceptually new understanding of river flood frequency across different climate regions.

Although the 2-year return period is a convenient proxy, it is not directly available for ungauged rivers and therefore cannot be defined globally without additional assumptions. For this reason, we do not consider the term "spatial refinement" to be appropriate.

- L71: Change to read "potentially introducing systematic differences in some regions in flood risk estimation."
- L120: Change to read: "...exhibits some over-estimation of bankfull width…"
- L123: Remove the word "slightly"
- L124-125: Delete the sentence starting with "This performance…" It is preferable, I think, to let the reader decide if the performance is acceptable.

We thank the reviewer for the above four comments (L71, L120, L123, L124).

In the revisions, we have changed the text accordingly.

- L126: Please add the units of RMSE to the values 1.32 and 1.66.

We thank the reviewer for this comment. In the revised manuscript, we have added the unit for RMSE.

Please note that these values (1.32, 1.66) represent the normalized root mean square error (nRMSE), defined as the root mean square deviation between estimated and observed values, normalized by the observed values. As such, it is dimensionless (see equation below). The equation is also provided in Supplementary Text S2 (Equation 7).

$$nRMSE = \sqrt{\frac{1}{n}\sum_{i=1}^{i=n}\left(\frac{Q_{BFobs} - Q_{BFest}}{Q_{BFobs}}\right)^2}$$

- L245: Change heading to "Differences in the commonly-use bankfull river discharge assumption" rather than biases. Your estimates could be biased as well and you don't have a truth to compare so you really are assessing differences. Same in Figure 4 captions - replace biases with differences. Replace within text elsewhere this occurs. You use differences in L285 so I don;t think it takes away from anything.
- L284: Change to "Our results assess"
- L307: Replace "improved" with "refined"
- L301 and L304: Change bias to differences

As we explained above (response to L49), we feel it is not appropriate to use the term 'refined' and 'differences'.

- L309" Remove the word "slightly." It is unclear how one could be so sure additional updated data would only "slightly" change estimates.

We thank the reviewer for pointing this out.

In the revisions, we have removed the word slightly, and revised the sentences as below.

'The median values and associated uncertainties reported here may be refined as additional observational estimates of bankfull discharge become available. Nevertheless, such refinements are not expected to alter the large-scale climate-associated spatial pattern identified in this study. Indeed, analysis of return periods of bankfull discharge derived from independently sourced, systematically measured datasets confirms consistent spatial variation across climate regions, supporting the observed increase from tropical to temperate, cold, and arid climates (Supplementary Fig. S15).'

- In L386, the statement is made that this work "refutes" the prevailing assumption of the 2-year bankfull recurrence interval. However, as stated above, one could argue this work

remarkably validates the bankfull recurrence of two years, a finding which is even more amazing given that the spatially-invariant value was determined without the level of detailed data used here. Even later in L399 the authors admit that “the 2-year assumption therefore provides a reasonable approximation…” and so it appears contradictory to the statement in L386.

- L394: Reword to state “highlight the systematic differences associated with the use of a spatially invariant bankfull return period in different regions.”
- L403 makes the statement that the two-year assumption should be “obsolete” but I disagree. The 2-year assumption is shown to be remarkably good on an average basis. Furthermore, you have not tested smaller rivers and future work could further validate the 2-year assumption. I would delete this sentence.

We thank the reviewer for these suggestions. In the revised manuscript, we have carefully reframed our narrative around the 2-year return period assumption for bankfull discharge, emphasizing that this assumption is often a reasonable approximation of bankfull discharge magnitude. However, our work takes an important scientific step forward by identifying and explaining the variability of bankfull discharge return periods and its systematic patterns across climates. We have therefore clarified the implications of using the 2-year assumption and resolved ambiguous or potentially contradictory statements. We also adjusted the tone for 2-year assumption as below.

‘*Hence, the assumption that bankfull discharge occurs consistently every two years on average is unlikely to be appropriate, especially in tropical and arid regions*.’

As noted above, we respectfully disagree with the reviewer’s characterization that our study assesses differences between two methods of estimating bankfull discharge. The 2-year return period is not conceptually the same as bankfull discharge. We would only use the term “difference” if we were comparing our observations with another independent estimate of bankfull discharge derived from field measurements. In contrast, the 2-year return period is an assumption and is not intended to measure bankfull discharge.

Furthermore, although additional analyses of smaller rivers may reveal apparent agreement between the 2-year recurrence assumption and bankfull discharge, such agreement does not eliminate the need for expanded observational constraints and approaches that explicitly account for variability.

2) Validation of the method

2a) In reading the main body of the text and the methods, I could not locate whether you repeatedly resampled 80% of the data to gain a more robust validation of the results rather than only one sampling of the dataset for validation. The Methods section notes a fivefold procedure but can you please clarify the number of times you resampled the dataset?

We thank the reviewer for this comment. We repeated the train–test split 10 times (parameter *n_run* in the code). For each run, we set a different random seed (*random_state*, we used an arbitrary base value plus the run index *i*) to ensure that the training and testing samples varied across runs. For each run, we computed error metrics for: (1) the training set, (2) five-fold cross-validation (averaged across folds), and (3) the testing set. In addition, these metrics were calculated separately for each of the four climate regions, such that each climate has its own set of evaluation results for cross-validation and testing categories.

We then selected the best-performing model (based on the combined RMSE from the training and testing sets) for the global estimation. In our main text, we reported the best model performance (Line 120-134), with associated figures provided in Supplementary Fig S7-8. We also reported the averaged error metrics as described above as Supplementary Table S2.

In the revised manuscript, we have clarified this procedure and strengthened the description of model validation and evaluation in the Method. We also added an additional figure showing cross-validation performance across folds with the tuned parameter set and annotated error metrics annotated (Supplementary Fig. S18, see below).

To ensure full transparency, we provide detailed code for all steps, including data splitting and error metric calculations. All codes associated with this procedure is included in https://github.com/YinxueLiu/G-QBF/scripts/RandomForest.py

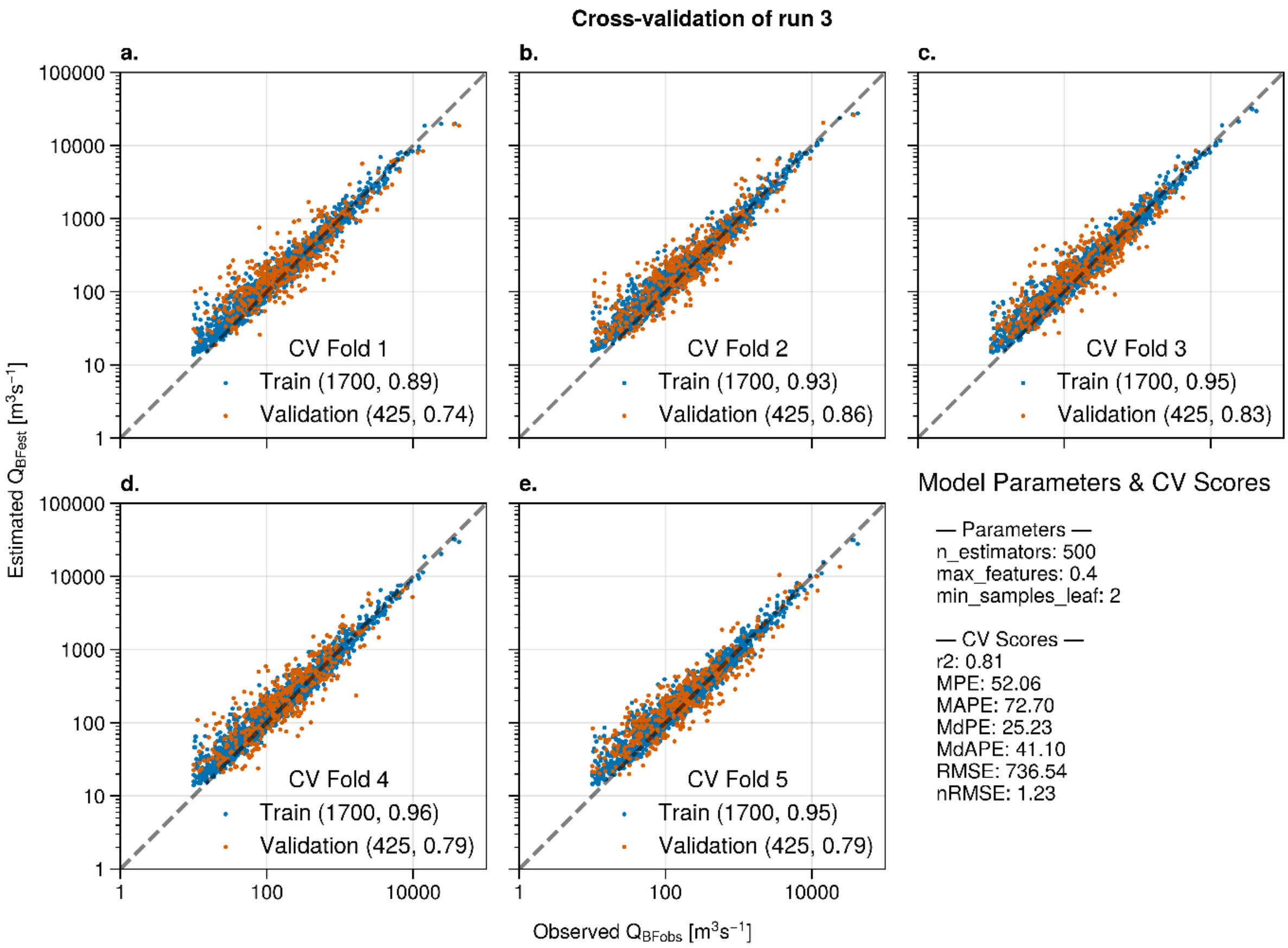

Fig. S18. Five-fold cross-validation results from one run (out of 10 model runs), shown with the tuned parameter set and associated error metrics.

2b) Use of variability as a proxy for accuracy of out-of-sample estimates

- L159-160: The statement is made "We use the coefficient of variation (CV) of the RF's per-tree predictions as a simple proxy for relative uncertainty where observations are unavailable." I do not agree with the use of the CV of the estimates as a method to quantify uncertainty. Variability of the estimates alone - as measured here by the CV - is not a valid way to assess accuracy. For example, estimates can have a low variability but be completely wrong. I would remove statements referencing the use of variability of the estimates to make any comment on the uncertainty of the estimated values. You have no observations for out-of-sample predictions with which to make any statement as to how the model performs for these cases.

- Again we see this logic in L222-225 that somehow variability of the results for out-of-sample estimates are somehow justification for the performance of out-of-sample estimates. Again, the consistency in estimates could be due to a systematic bias in your results rather than a commentary on accuracy. I would remove this statement. You have no way to assess the actual accuracy of out-of-sample estimates and any attempt to justify their validity through variability of the estimates is not appropriate. The work is already strong and these types of statements detract from the strength of your findings.

We agree with the reviewer that the coefficient of variation across random forest trees does not always translate directly to estimation error. In the revised manuscript, we removed the content relevant to this.

3) Limitations of the analysis

If I am reading correctly, it appears from L107 that the training dataset contains mainly large rivers that are 30 m or wider. As typical with machine-learning approaches, the results of the model should not be applied to locations that fall outside of the range of conditions under which the models were developed, and the authors make note that out-of-sample locations were removed. However, it would be helpful to explicitly state the bounds of the training dataset. Different locations in the text (Figure 1, Methods section, as examples) explain that out-of-sample reaches account for 12.3% of the total reaches modeled (L154) but it is not clear what the thresholds were for drainage area and river width for a reach to quality as out-of-sample. Please note those values explicitly around L154, Figure 1 or the Methods section.

We thank the reviewer for this comment. In the revised manuscript, we added the range of river width and drainage area that are used to define the out-of-sample data explicitly (Line 160-164). We also revised figure 1 by adding additional quantification of the out-of-sample ratio identified by river width, drainage area, and both factors as a chart.

4) Improved clarity and communication

4a) Be consistent with your abbreviations. There are many cases where you have abbreviated a term but then still spell it out later. I have made note of these cases but it is possible that I missed some. Please carefully edit your manuscript with an eye towards consistency:

L112: "bankfull discharge observations" should be abbreviated to Qbfest.
L130: "estimated Qbf" should be changed to Qbf-est
L168: "estimated Qbf" should be changed to Qbf-est
L172: "Qbf estimates: should be Qbf-est
L191: "return period of the bankfull river discharge" to QBF-RP
L195: bankfull river discharge return periods to QBF-RPs
L221: observed and estimated bankfull discharge to QBF-obs and QBF-est
L223 and L224: use abbreviations for QBF-est
We thank the reviewer for pointing this out. In the revisions, we checked this throughout the manuscript and have revised accordingly.

4b) Figure 1: I question the value of the maps in this figure. They are difficult to read and it is unclear what the reader is expected to observe from the maps. I also could not locate any grey lines showing the out-of-sample rivers.

We thank the reviewer for this comment.

In the revised manuscript, we have substantially improved Figure 1 to better highlight the key information: (1) the spatial distribution of estimated bankfull discharge across global rivers, and (2) the spatial distribution of out-of-sample rivers by basin. We have also moved the zoomed-in map to Supplementary Fig. S11. We removed the coefficient of variation.

4c) L165 states that "differences are statistically significant" but I could not locate what hypothesis test was used. The Dunn test and K-S test are mentioned later in the text but it is not clear if either of those tests were the one used here.

We thank the reviewer for pointing this out. In the revisions, we made the statement in the main text explicitly as follows: (Line 200-204)

For both observed and estimated bankfull discharge, the distributions of Q_{BF-RP} differed significantly across climate regions (Kruskal–Wallis H test, $p < 0.001$). Post hoc Dunn's tests further indicated significant pairwise differences among nearly all climate-region pairs ($p < 0.001$), except between tropical and temperate climates for $Q_{BFobs-RP}$, for which the difference was weaker ($p < 0.05$).

We also improved the statement in the **Methods** section as: (Line 595-598)

We employed the Kruskal–Wallis H test to assess whether bankfull discharge return periods differed among climate regions (null hypothesis: samples were drawn from the same distribution), followed by Dunn's post hoc test for pairwise comparisons.

4d) The wording around bankfull recurrence interval could be improved. I offer some alternative wording to address this point:

- L39: Consider revising to read "...can transport before spilling over its banks, also known as the bankfall discharge…" This change can help distinguish large flows from overbank flow. Note that you also use a different explanation for bankfull discharge in L59 where you state bankfull discharge is the "maximum flow rate contained within a river just before inundation occurs on to the surrounding floodplain." It would improve clarity to be consistent in the manuscript as to your definition of bankfull discharge,

We agree with the reviewer. In the revisions, we made the definition of bankfull discharge consistent as overbank flow and avoided using the term flooding.

- L42: The current wording of "we deliver the first global estimation of bankfull discharge" reads as though you are providing one global value for all rivers. Consider revising to "we deliver the first update to global estimates.."

We thank the reviewer for raising this point. While we agree that the original wording may have been ambiguous, we do not consider the term "the first update" to be appropriate, as no prior global estimates existed before our work.

We revised the sentence to "*we deliver the first spatially distributed global estimation of bankfull discharge*".

- In L176-181, it would be helpful to be more precise. For example, consider rewording to "...we calculated the return period for both QBF-obs and QBF-est distributed across the four climate regions. The return periods corresponding to bankfull river discharge (QBF-RP) vary widely across the globe. Overall, almost a quarter of sites are expected to exceed QBF several times a year on average…We find 15.6% of sites with bankfull discharges corresponding to a return period of 3.5 years…"
- L183: Change to read "..indicates that longer return periods (>3 years) associated with bankfull discharge predominantly occur in arid regions and are less common in tropical

climates.

- L186 is confusing. Bankfull discharge is one value and it is unclear what is intended by the statement "indicating more frequency bankfull status." Consider revising to "Shorter return periods, indicating the probability of more frequent occurrence of discharges that exceed QBF…"
- L188-189 is also awkwardly worded. Consider "The median return period of the discharge associated with the observed (estimated) QBF-RP increases…"

We thank the reviewer for offering the writing suggestions. In the revisions, we have made all the changes accordingly.

- Consider changing "out-of-distribtuion" to "out-of-sample". In my reading, distribution implies some type of probabilistic assessment.

We thank the reviewer for this suggestion. We have replaced the term 'out-of-distribution' with 'out-of-range' throughout the manuscript and Supplementary Information. We adopt 'out-of-range' to more directly reflect cases where predictor values fall outside the range of the training data, without implying a probabilistic distributional assumption. The term has been updated for clarity and consistency.

4d) Editorial comments

L60: Change to read "This critical flow value…"

L67: Consider stating the number of rivers rather than saying "For most of the 37 rivers surveyed…"

L82: Consider revising to read: "Collecting such data is time-intensive and costly, and therefore, bankfull discharge…"

L88-90: Consider revising to read: "a host of novel global datasets have emerged that can be used to estimate bankfull discharge. These datasets include the location and connectivity of rivers, observation-based river widths, and dam and reservoir information."

L92: Consider revising to read: "this rich variety of emerging datasets coupled with machine learning approaches…"

L93-96: Consider revising to read: "We present the first estimates of bankfull river discharge across the globe (GQBF), revealing distinct patterns of bankfull discharge return periods across climate regions, and compare the use of the 2-year return period bankfull discharge assumption relative to the GQBF.

We thank the reviewer for the editorial comments. In the revisions, we changed all the sentences accordingly. (Line 96-100)

"*We present the first spatially distributed global estimation of bankfull river discharge (GQBF), revealing distinct patterns of bankfull discharge return periods across climate*

regions, and quantify the differences of the common 2-year return period bankfull discharge assumption relative to observations."

Please note that since we only compared the 2-year return period bankfull discharge assumption with observed bankfull discharge, we slightly changed the wording from the reviewer's suggested version.

L169: Reword to "higher for drainage areas smaller than 1000km2 and located in arid climates.
L316-317: Reword to state "... aligns with previous observations in localized areas that report highly variable.." Otherwise this sentence reads as though others have already done this analysis globally. I had to go to the citations to see that these cited studies were for local areas and not global analyses.
We thank the reviewer for the above suggestions and revised the sentences accordingly.

L341-344: Enumerate the 5 limitations that you explain further in the paragraph. I only see 3 listed in this introductory sentence but you actually number and explain 5 things in the paragraph.
We thank the reviewer for pointing this out. In the revisions, we improved the introductory sentence as (Line 318-321)

"These include uncertainty in high flow measurements, temporal and spatial variability within river reaches, biased representation towards perennial rivers, and limited information of channel types and river regulation."

We also reorganized the points to align with the introductory sentence.

L345-371: Excellent descriptions and analysis
We thank the reviewer for this kind comment.

L505: You are already abbreviate upstream accumulated (CS).
L528: Weird spacing in the text.
L531: You already abbreviate river catchment (CA).
L552: You already abbreviate river catchment (CA).
We thank the reviewer for this comment. We improved the spacing in the revisions. We also replaced the text after the abbreviated definition to CS, CA accordingly.

Figure 3a. Consider the text by the left arrow to read "bankfull discharge occurs less frequently than the mean annual flood" and the right arrow "Bankfull discharge occurs more frequently than the mean annual flood"

We thank the reviewer for this suggestion. We revised figure 3a accordingly (see below).

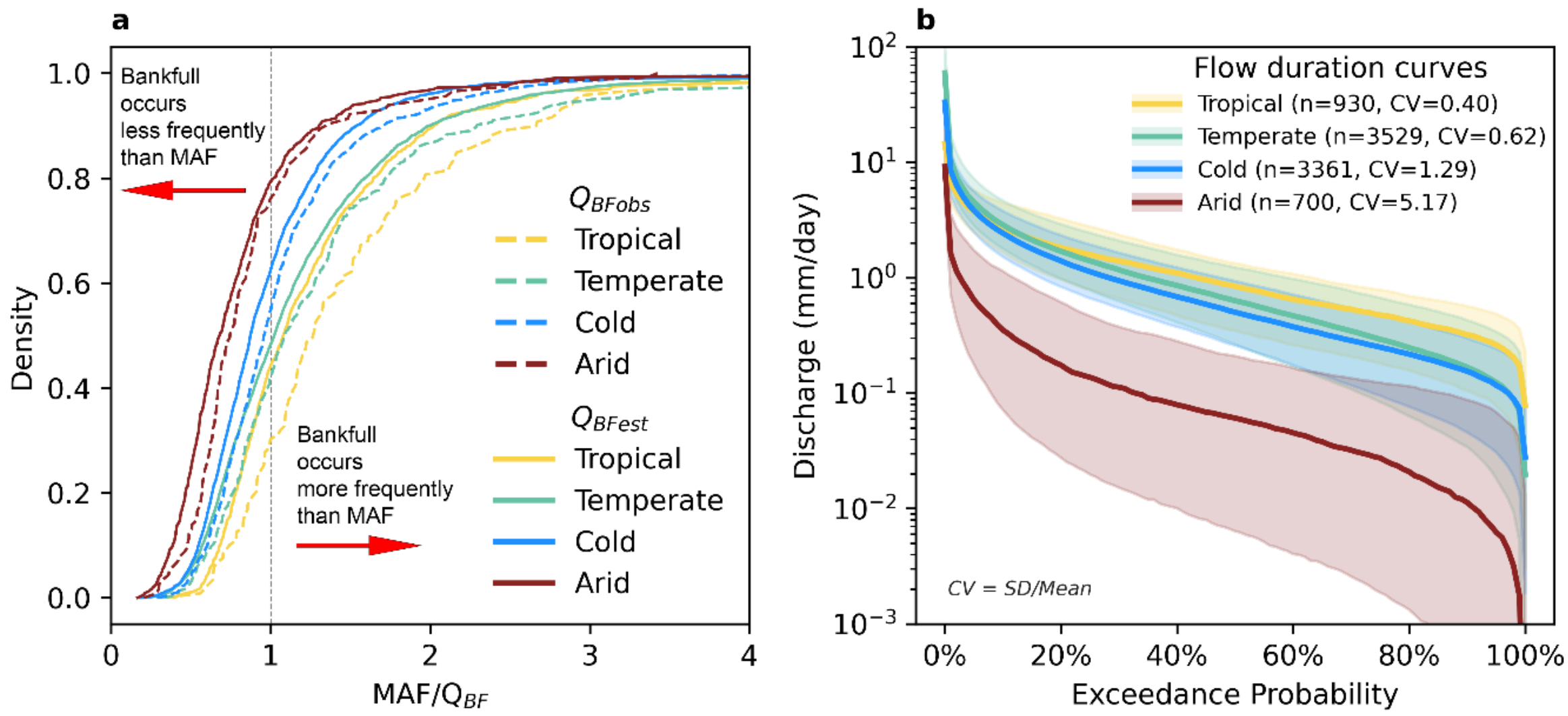

Reviewer #3 (Remarks to the Author):

Please find the attached document for my full review.

Review: "Global Estimation of Bankfull River Discharge Reveals Distinct Flood Recurrences Across Climate Zones"

By Liu et al., Submitted to Nature Communications

(review by J. A. Nittrouer, Texas Tech University, U.S.A.)

Primary Comments:

I found this study to be remarkably comprehensive, providing what I estimate to be the most thorough examination of bankfull flow conditions for rivers globally. It's certainly of high enough quality and original research to be published in Nature Communications, however throughout my read, I couldn't help thinking it would be a better fit for Nature Geoscience, where it could likely reach a more engaged/targeted audience. I recognize that such a decision is made by the discretion of the journal editors.

The primary contribution of this study is to quantify the return frequency of river floods as a function of climate regions. The main message is that the oft-cited two-year recurrence interval for bankfull flow events is unlikely across a range of system types. I'm certain most fluvial hydrologists and geomorphologists intuit this situation, but where this study makes such a unique contribution is using new methodologies (namely, AI) to produce a robust statistical quantification of how and where rivers deviate from the two-year bankfull assertion. The implications speak for themselves, as the authors discuss in detail how, in a world of freshwater scarcity, infrastructure protection, and changing discharge conditions tied to dynamic hydrological cycles, paying attention to flood conditions is ever more critical.

We are pleased that our work was well received by Prof Nittrouer and appreciate his suggestion regarding journal choice. We will certainly consider submitting future work on this topic to *Nature Geoscience*.

We also thank Prof Nittrouer for his thoughtful summary of our contributions and for the time he invested in reviewing our manuscript and providing constructive feedback.

In the revised manuscript, we have made substantial improvements to the figures and overall readability. All line numbers in this document refer to the clean revised manuscript.

The reason why I would recommend "major revisions" is because, while very well written, I think the text can be more thoroughly streamlined to improve the manuscript's punchiness.

To start, through numerous subfigures, the authors fit something like 13-15 figures into what amounts to be the space of four figures. I understand why they're doing this: because the

figure count is limited in this short-format journal. However, this shoehorning distracts from the message of the paper, while rendering the size of the figures sufficiently small so that it is very difficult (without a magnifying glass) to read the content. As such, I would recommend cutting at least half of them from the main text; in particular, the numerous global maps and plots, and placing them in Supplementary Materials. Additionally, this would lower the word count of the figure captions, which total (by my quick estimate) roughly 700 words across the four figures. Additionally, I think that some of the analytical details could be moved to SM as well; for example, lines 185-197. In short, revisions should aim to make the paper more concise, thereby improving its readability.
We thank Prof Nittrouer for his suggestions on improving the presentation of our work.

In the revisions, we have substantially improved all the main figures. Specifically, we moved some of the panels that contained secondary information in Figure 1 to the Supplementary Materials. We simplified Figure 2 and Figure 4 by removing some of the panels.

Following these revisions, we also simplified the figure captions for Figure 1, Figure 2, and Figure 4. We include the revised figures and their captions here. We also reorganized the descriptions in the main text to reflect these changes to all the figures.

Overall, we believe these changes have made the paper more concise and improved its readability.

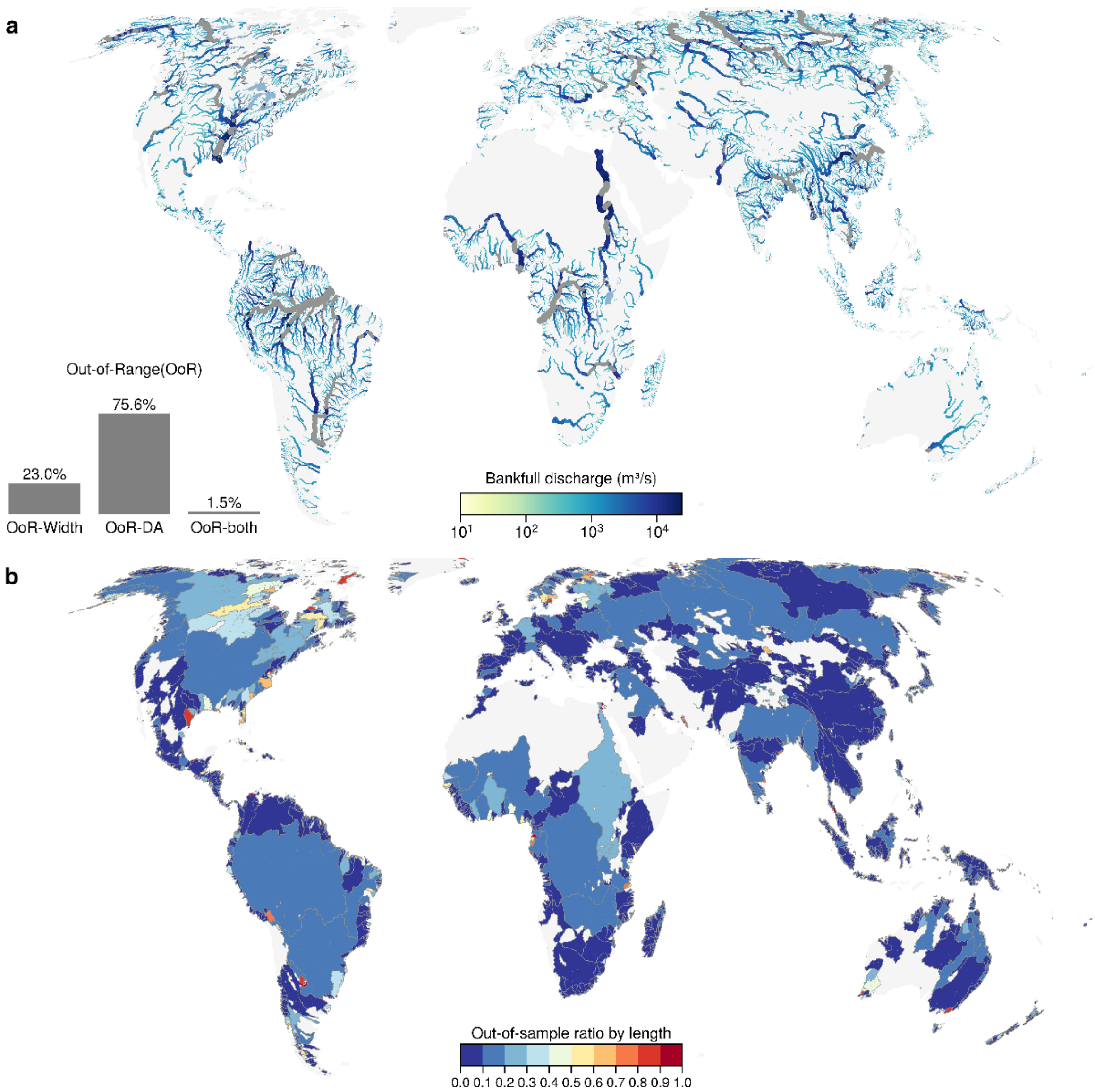

Fig. 1 | Bankfull river discharge estimation in ~2.87 million 1-km reaches. a, Bankfull river discharge along the GRIT global river network. Predictions are displayed at GRIT segment scale (aggregated median value of reaches) for visual clarity. Out-of-range estimations are removed (coloured in gray). The left inset indicates the length ratio of out-of-range data identified by river width, drainage area and both. b, Fraction of river-network length that is out-of-range within each GRIT basin, defined as the proportion of total river-network length where river width or drainage area falls outside the training-data range.

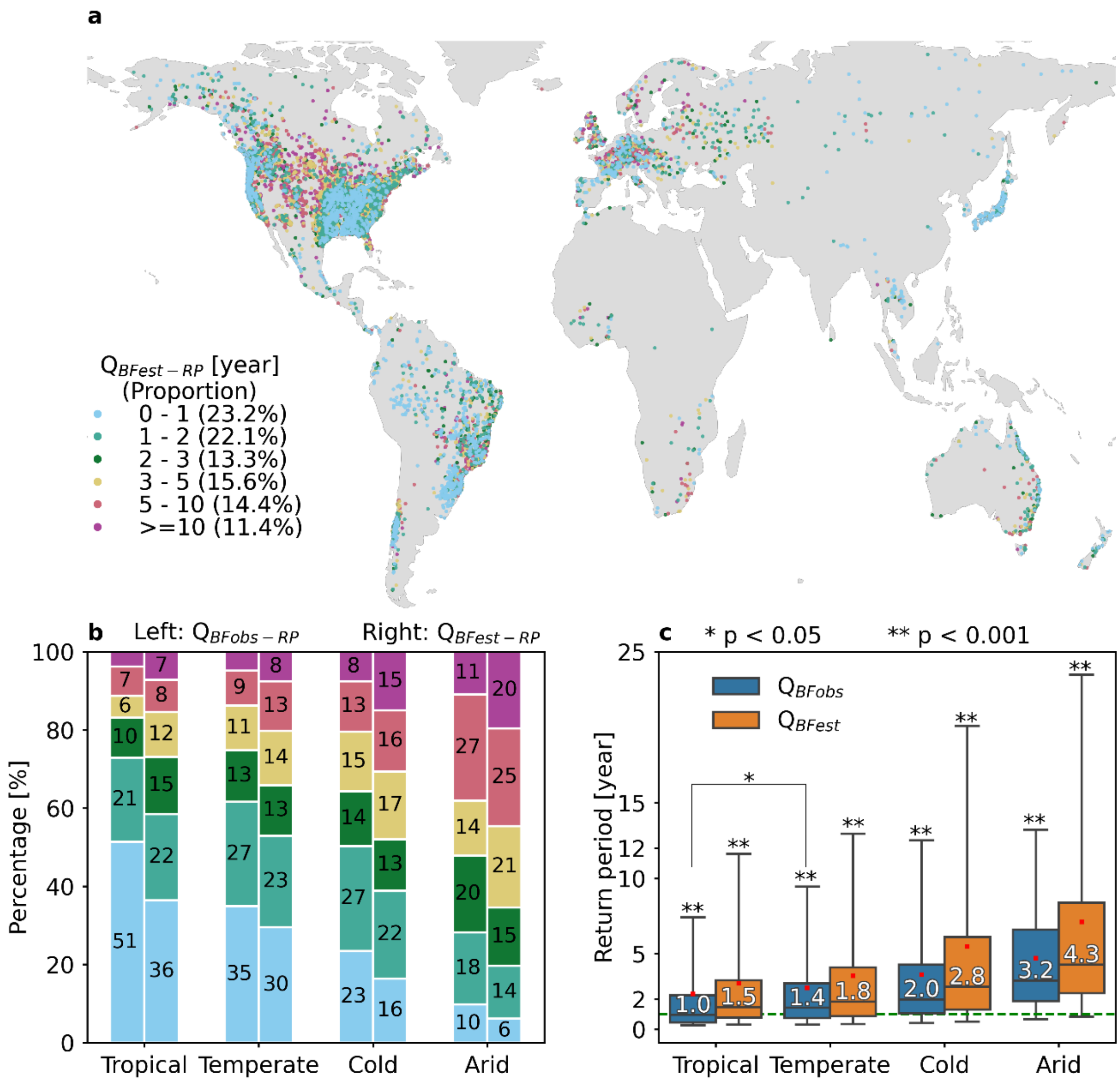

Fig. 2 | Variation of bankfull river discharge return periods (QBF-RP) across four climate regions: Tropical, Temperate, Cold, and Arid. a, Estimated bankfull discharge return periods ($Q_{BFest-RP}$), with colours indicating return period ranges (0-1, 1-2, 2-3, 3-5, 5-10, and ≥10 years); proportions are given in brackets in the inset legend. b, Proportion of gauging stations in each return period category (coloured, colour scale is the same as panel a), for each climate region, with observed values shown on the left bar and estimated values on the right bar. c, Distribution of observed and estimated return periods ($Q_{BFobs-RP}$, $Q_{BFest-RP}$) in each climate region. The boxplot limits (horizontal lines), from bottom to top, mark the 5th, 25th, 50th, 75th, and 95th percentiles, respectively; red squares mark the mean values; labels represent the median. Asterisks indicate the significance level of the observed (estimated) return period of bankfull discharge within a given climate region different from all the other three climates.

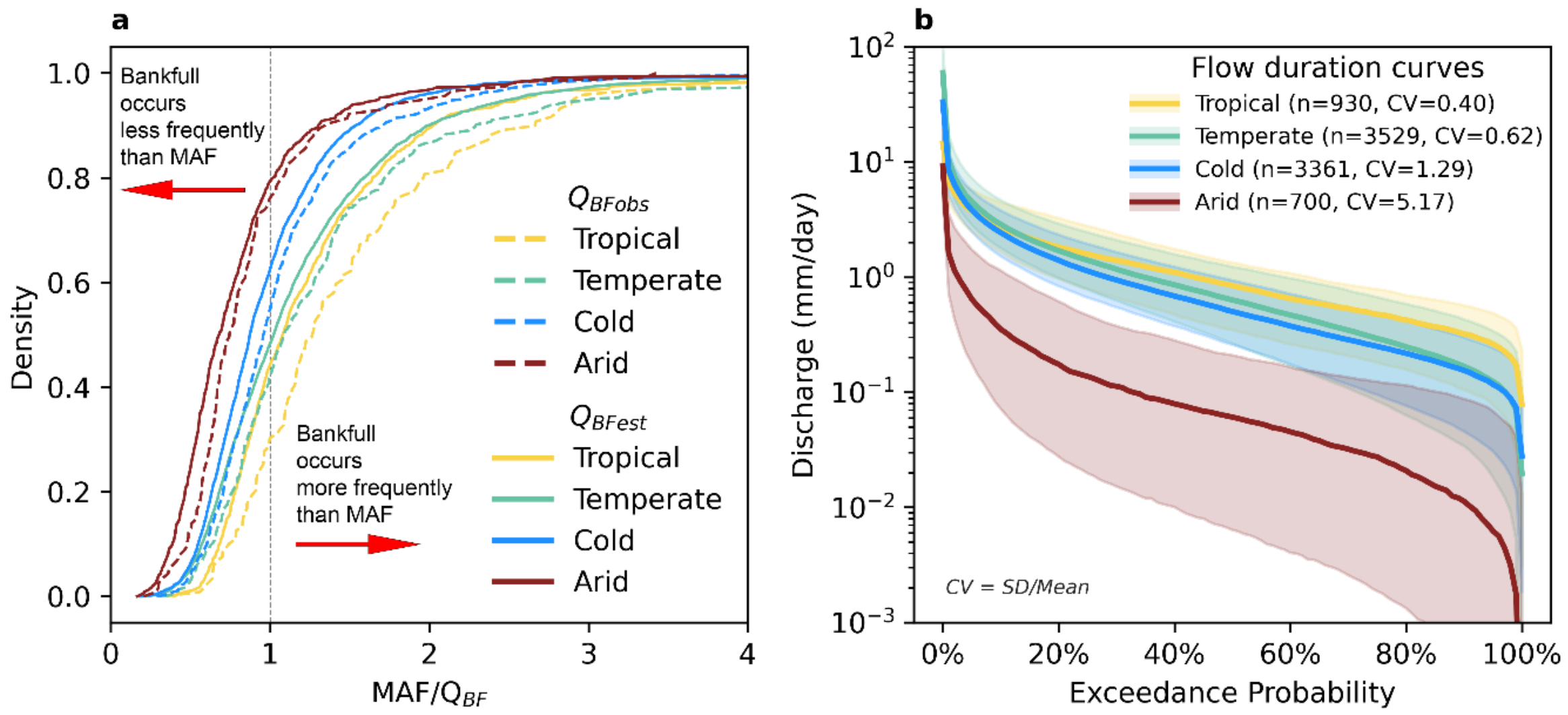

Fig. 3 | Variability in bankfull discharge and daily streamflow across four climate regions: Tropical, Temperate, Cold, and Arid. a, Probability density distribution of the ratio between Mean Annual Flood (MAF) and bankfull discharge, shown for both observed (Q_{BFobs}) and estimated (Q_{BFest}) values across climate regions. b, Cumulative flow duration curves showing daily discharge versus exceedance probability in four climate regions, based on 8,576 gauges. Solid lines indicate the median; transparent ribbons show the 25th-75th percentile across all stations within each climate. The number of stations and coefficient of variation (CV) of streamflow are shown in the legend.

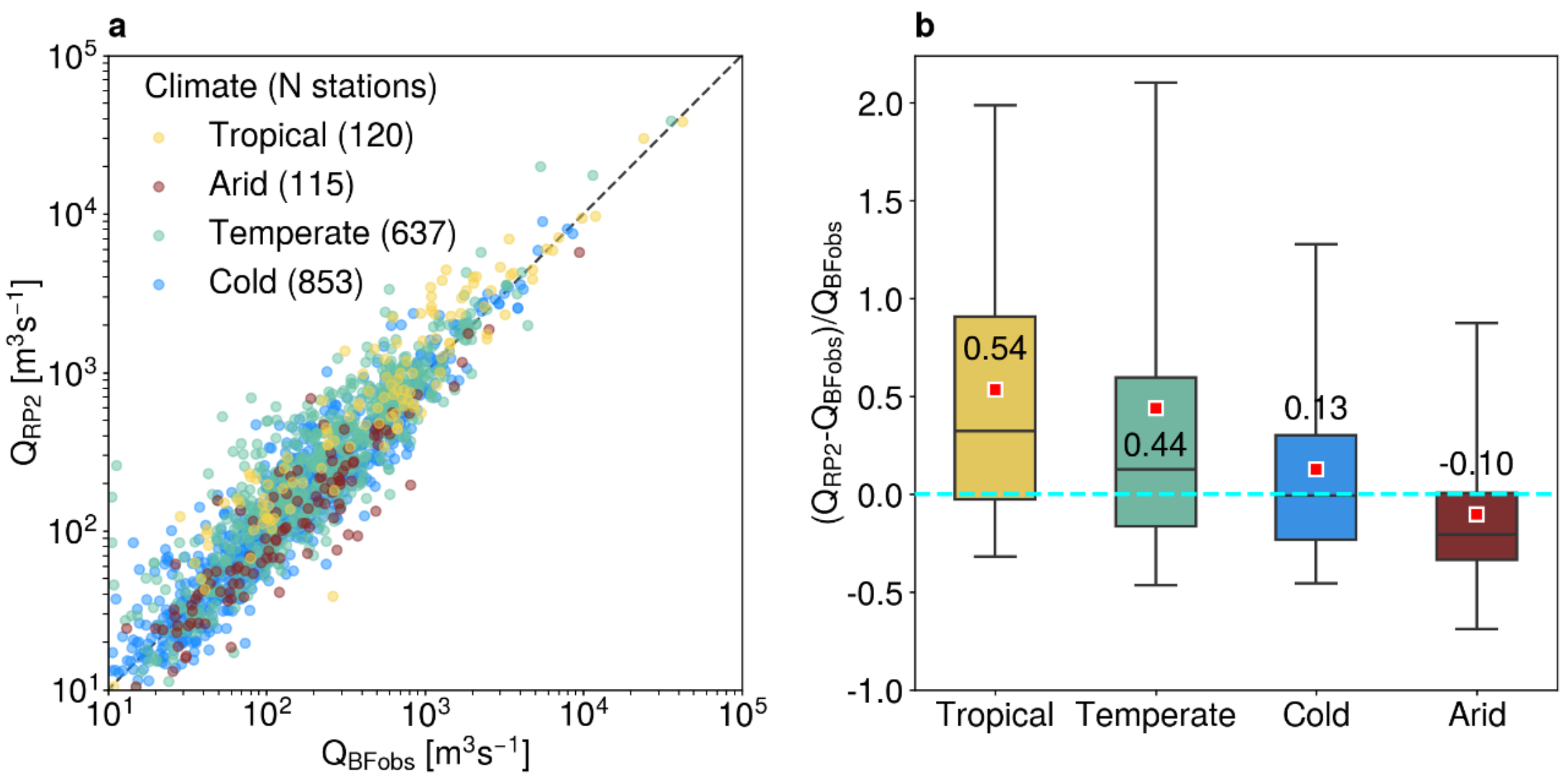

Fig. 4 | Biases of the widely used 2-year return period bankfull river discharge (Q_{RP2}) assumption across four climate regions: Tropical, Temperate, Cold, and Arid. a, Scatter plot of the 2-year return period discharge (Q_{RP2}; y-axis) and observed bankfull discharge (Q_{BFobs}; x-axis) in log scale. b, Distribution of the 2-year return period bias compared to bankfull discharge observations in each climate region. Horizontal boxplot lines from bottom to top mark the 5th, 25th, 50th, 75th, and 95th percentiles, respectively; red squares mark the mean values with numbers displayed. Horizontal cyan line marks the location of bias=0.

To be sure, this is not to say that I don't appreciate details. For example, I welcome the

author's discussion around model sensitivity and limits (lines 318-320, 327-333) and think that this type of recognition should stay in the primary document. Additionally, I'm sure a study such as this garners many reviewer comments about testing bankfull recurrence timing for other controls; as such, I am interested to know how factors such as basin size, sediment type, and anthropogenic modifications to rivers systems could impact flood return periods.

We thank Prof Nittrouer for these helpful suggestions and for highlighting the importance of clearly presenting model sensitivity, limitations, and potential additional controls on bankfull recurrence. We agree that these aspects improve transparency and have therefore moved the relevant discussion of model sensitivity and limitations into the main text (Lines 140–152).

Following the suggestion, we also examined the dependence of bankfull discharge recurrence on drainage area. A tendency toward shorter recurrence intervals with increasing drainage area is observed (Fig. R2 below). Because this study does not explicitly test the mechanisms controlling this pattern, and doing so would require additional analysis beyond the present scope, we do not further interpret this result, but we include it here for completeness.

With respect to other potential controls, such as sediment size and anthropogenic modifications, we agree that these factors may influence bankfull recurrence. However, for sediment characteristics, we were unable to conduct a quantitative analysis due to the lack of consistent sediment-type data co-located with both gauging records and bankfull discharge observations. A rigorous analysis of such controls would require a dedicated dataset and further mechanisms examinations and is therefore beyond the scope of the present study; we have added a brief statement acknowledging this limitation in the Discussion (Lines 275–278).

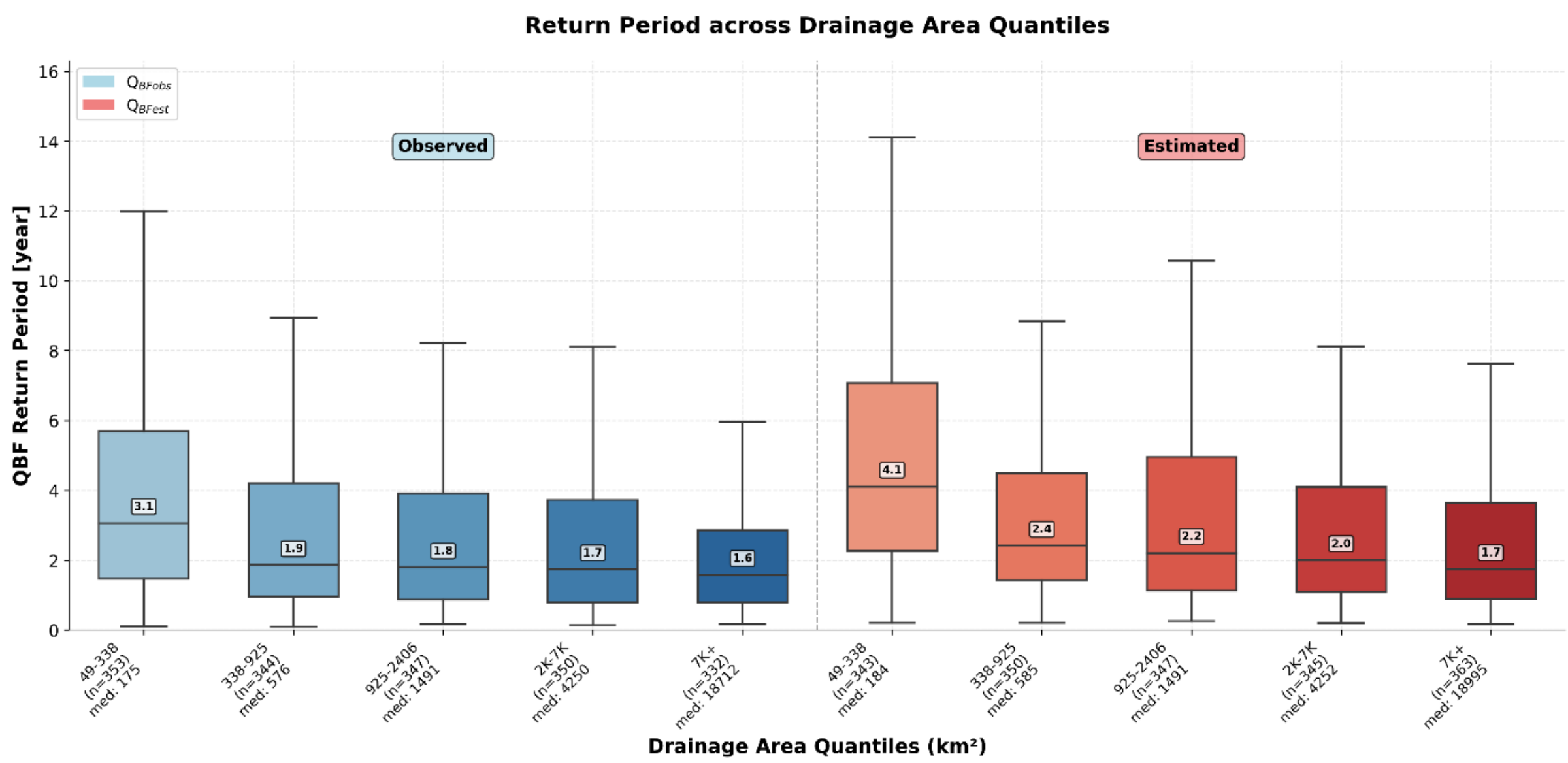

Figure R2. Bankfull discharge return period across drainage area quantiles for observed, and random forest estimated bankfull discharge values. Horizontal boxplot lines from bottom to top mark the 5th, 25th, 50th, 75th, and 95th percentiles, respectively; The median values are displayed.

In all, I appreciate the authors' efforts and recognize that this new AI methodology to capture global rivers stands to improve via refinement with future data sets coming online or soon to be available, for example integrating SWOT measurements. Their model serves as an important foundation that will likely only be improved upon in years to come.

We thank the reviewer for this kind comment. We agree that SWOT presents an exciting opportunity to help us improve future estimations at the global scale.

In the revised manuscript, we added the following citation as an example of recent work on the use of SWOT for quantifying global river bank shapes (Cerbelaud et al., 2026).

References:

Cerbelaud, A., Wade, J., David, C.H. et al. Wide-swath altimetry maps bank shapes and storage changes in global rivers. Nature 651, 666–671 (2026). https://doi.org/10.1038/s41586-026-10218-y

Short comments:

Line 153 and elsewhere: Please explain "out-of-distribution river reaches", which show up in a few locations and refers to river reaches for some of the biggest systems globally (i.e.,Mississippi, Yangtze, Amazon etc.). How/why are these systems "out of distribution"? To be sure, I looked through the main text and could not determine what this means.

We thank the reviewer for pointing this out.

In the revisions, we added a definition of out-of-range (we changed the term from out-of-distribution to out-of-range to be concise, following review2's suggestion). Additionally, we explained the reason for masking out the estimations in these out-of-range areas.

"*To limit uncertainty in bankfull discharge predictions arising from extrapolation, we flagged locations with predictor values beyond the training range, where model predictions are inherently unreliable*[38]*. We used two predictors, DEM-derived drainage area and observation-based river width to identify out-of-range locations, i.e., reaches with values outside 30 – 1,673 m for river width or outside 50.23 – 3,425,560 km*^{2} *for drainage area.*"

These are added to the main text (Line 160-164).

In the discussion, we also discussed some potential solutions to fill the gaps for these out-of-range areas (Line 307-316).

Line 228: I recommend changing the text of "…varies with climate across *our* sites" (my emphasis) to a word choice that do not include a plural first-person possessive pronoun, as this does not flow stylistically with the manuscript text.

We thank the reviewer for this comment. We revised the sentence accordingly (Line223-224).

"*The ratio between the bankfull discharge (Q_{BF}) and mean annual flood (MAF) reveals how bankfull frequency varies with climate across the study sites.*"

REVIEWERS' COMMENTS

Reviewer #1 (Remarks to the Author):

I think the authors have carefully addressed all my comments and the dataset is a good contribution to the community. I have no further comments on the main texts except for some minor issues regarding code availability.

Reviewer #1 (Remarks on code availability):

The authors have added more details regarding the code, but the results are still not fully reproducible because the training data cannot be released. In this case, can the authors consider giving some working examples? Currently what's in the /example directory is not workable examples. This reduces the reproducibility. More strategies to improve reproducibility is highly recommended.

We thank the reviewer for this comment. Following the reviewer's suggestion, we have added working examples, with the input data and reproducible outputs in the revised code repository. Detailed instructions for reproducing the analysis are provided in the README.md file, and the key reproducibility notes are also included below.

'3. How to run

Run the scripts in the following order:

1. *Train and evaluate model*

```
python scripts/RandomForest.py --station_qbf=RF/Input/observed_qbf.csv --
station_predictors=RF/Input/station_predictors.csv --
koppen_color=RF/Input/Koppenlabels_colors.csv --
output=RF/Output/trained_model.joblib --
output_performance=RF/Output/model_performance.csv --
ouput_csv=RF/Output/qbf_train_test.csv --n_run=1
```

2. *Analysis the return period of bankfull discharge and 2-year return period of floods*

```
R scripts/FFA_POT.R --station_info examples/FFA-
POT/Input/GRITv06_stations_QBFobs_USGS_05453100.csv --dir_path examples/FFA-
POT/Input/daily_streamflow --loess-span 0.5 --output Output/USGS_05453100.csv
'
```

'5. Reproducibility notes

Using the data provided in the examples/RF/Input with the code above (scripts/RandomForest.py) should reproduce the results in examples/RF/Output

Using the data provided in the examples/FFA-POT/Input with the code above (scripts/FFA_POT.R) should reproduce the results in the examples/FFA-POT/Output'

Reviewer #2 (Remarks to the Author):

I have now read the responses to the review comments and the revised manuscript. The authors did an exceptional job in thoughtfully revising the manuscript according to the reviewer comments. Notable improvements include a complete revision of the figures in the manuscript, expansion and clarification of the methods, and a detailed sensitivity analysis of the methodological choices used to estimate the return periods.

While there are limited instances where the authors disagreed with my comments, I fully accept their rebuttal and reasoning. Even in those instances, the authors thought carefully about the spirit of the comment and further strengthened the manuscript.

I have no further comments and recommend the manuscript be accepted. It was a delight to be part of the colleague review process and I look forward to seeing the manuscript published.

We are delighted that our revisions have been well received, and we are grateful to the reviewers for their constructive feedback, which has helped improve the manuscript.

Review: “Global Estimation of Bankfull River Discharge Reveals Distinct Flood Recurrences Across Climate Zones”
By Liu et al., Submitted to Nature Communications
(review by J. A. Nittrouer, Texas Tech University, U.S.A.)

Primary Comments:
I found this study to be remarkably comprehensive, providing what I estimate to be the most thorough examination of bankfull flow conditions for rivers globally. It’s certainly of high enough quality and original research to be published in Nature Communications, however throughout my read, I couldn’t help thinking it would be a better fit for Nature Geoscience, where it could likely reach a more engaged/targeted audience. I recognize that such a decision is made by the discretion of the journal editors.

The primary contribution of this study is to quantify the return frequency of river floods as a function of climate regions. The main message is that the oft-cited two-year recurrence interval for bankfull flow events is unlikely across a range of system types. I’m certain most fluvial hydrologists and geomorphologists intuit this situation, but where this study makes such a unique contribution is using new methodologies (namely, AI) to produce a robust statistical quantification of how and where rivers deviate from the two-year bankfull assertion. The implications speak for themselves, as the authors discuss in detail how, in a world of freshwater scarcity, infrastructure protection, and changing discharge conditions tied to dynamic hydrological cycles, paying attention to flood conditions is ever more critical.

The reason why I would recommend “major revisions” is because, while very well written, I think the text can be more thoroughly streamlined to improve the manuscript’s punchiness. To start, through numerous subfigures, the authors fit something like 13-15 figures into what amounts to be the space of four figures. I understand why they’re doing this: because the figure count is limited in this short-format journal. However, this shoehorning distracts from the message of the paper, while rendering the size of the figures sufficiently small so that it is very difficult (without a magnifying glass) to read the content. As such, I would recommend cutting at least half of them from the main text; in particular, the numerous global maps and plots, and placing them in Supplementary Materials. Additionally, this would lower the word count of the figure captions, which total (by my quick estimate) roughly 700 words across the four figures. Additionally, I think that some of the analytical details could be moved to SM as well; for example, lines 185-197. In short, revisions should aim to make the paper more concise, thereby improving its readability.

To be sure, this is not to say that I don't appreciate details. For example, I welcome the author's discussion around model sensitivity and limits (lines 318-320, 327-333) and think that this type of recognition should stay in the primary document. Additionally, I'm sure a study such as this garners many reviewer comments about testing bankfull recurrence timing for other controls; as such, I am interested to know how factors such as basin size, sediment type, and anthropogenic modifications to rivers systems could impact flood return periods.

In all, I appreciate the authors' efforts and recognize that this new AI methodology to capture global rivers stands to improve via refinement with future data sets coming online or soon to be available, for example integrating SWOT measurements. Their model serves as an important foundation that will likely only be improved upon in years to come.

Short comments:
Line 153 and elsewhere: Please explain "out-of-distribution river reaches", which show up in a few locations and refers to river reaches for some of the biggest systems globally (i.e., Mississippi, Yangtze, Amazon etc.). How/why are these systems "out of distribution"? To be sure, I looked through the main text and could not determine what this means.

Line 228: I recommend changing the text of "…varies with climate across *our* sites" (my emphasis) to a word choice that do not include a plural first-person possessive pronoun, as this does not flow stylistically with the manuscript text.
